# DualPhos: a versatile, chemoselective reagent for two-carbon aldehyde to latent (*E*)-alkenal homologation and application in the total synthesis of phomolide G

**DOI:** 10.1098/rsos.160374

**Published:** 2016-11-23

**Authors:** David McLeod, James McNulty

**Affiliations:** Department of Chemistry and Chemical Biology, McMaster University Faculty of Science, Hamilton, Ontario, CanadaL8S 4M1

**Keywords:** synthetic methodology, total synthesis, macrolactones

## Abstract

Advances on the use of the 2-pinacolacetal-tripropylphosphonium salt DualPhos as a general reagent for the two-carbon aldehyde to alkenal homologation and a chemoselective iron (III) chloride mediated deprotection are described. The strategy allows isolation of the latent alkenal intermediates or direct hydrolysis to (*E*)-alkenals. The robust chemical stability of the latent alkenals is demonstrated in a total synthesis of the macrolactone phomolide G.

## 1. Introduction

The two-carbon homologation reaction of aldehydes **1** to generate (*E*)-alkenals **3** (α,β-unsaturated aldehydes) is a strategic transformation often employed in fine chemical and pharmaceutical syntheses. The direct transformation **1** to **3** is problematic due to inherent functional group incompatibility and the high reactivity of the product, which often cannot be present during the reaction. A series of α-phosphono substituted acetaldehyde acetals **4** and **5** as well as the ylide **6** have been developed that allow one- or two-step aldehyde to alkenal conversions [1–17]. A general limitation of these processes is a lack of versatility as the alkenal must be formed (or is formed *in situ*) and reacted immediately (*vide infra*) due to the instability of the intermediate protected alkenal and/or product and is also the reason these conversions are often inefficient. As a result, in standard practice the transformation **1** to **3** is typically carried out indirectly through a three-step process involving homologation to the unsaturated ester by Horner–Wadsworth–Emmons (HWE) or related Wittig extension, DIBAL-H mediated reduction to the allylic alcohol followed by Swern or Dess–Martin periodinane-mediated oxidation. These steps must be conducted sequentially with dry solvents and use atom-uneconomical redox chemistry, cryogenics and column chromatography. The ready availability of many aldehydes and increasing utility of (*E*)-alkenals **3** as reactive substrates for aldol, cycloaddition, olefination and conjugate addition reactions and, more recently, as substrates in asymmetric organocatalytic reactions [18–21] have been driving factors in our search for efficient, versatile reagents to effect homologation reactions.

**Figure 1.**
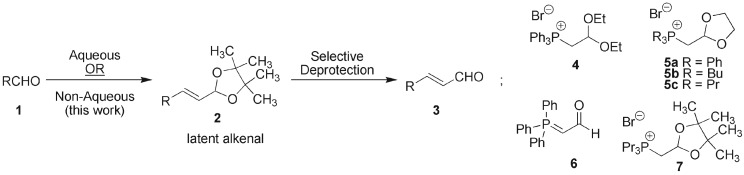
Aldehyde to alkenal homologation and select reagents **4**–**7** that have been employed.

We recently developed the tripropylphosphonium salt **7**, containing a chemically robust pinacolacetal functional group and demonstrated its use in *aqueous* Wittig reactions of aldehydes, generating stable latent alkenals **2** (figure 1) which could be readily isolated or converted to the free (*E*)-alkenals **3** by mild hydrolysis [22]. The use of ylides derived from short-chain trialkylphosphines in Wittig chemistry allows for high (*E*)-olefin stereoselectivity, the reactions may be conducted in aqueous media and permit simple removal of the water-soluble phosphine oxides [23–27]. (The acronym DualPhos was coined for reagent **7** in view of the ‘dual’ activity of the reagent as an effector of two-carbon homologation and the extended utility of retaining the latent alkenal until the deprotected α,β-unsaturated aldehyde is required. For earlier homologation strategies see [22–29].) The original process using salt **7** was carried out in water using bases such as NaOH and was shown to be efficient for non-enolizable aromatic aldehydes. We became acutely aware of the methodological limitations of reagents **4**–**6** during a recent synthesis of the natural nonenolide phomolide G, in which we utilized the functionalized salt **5c** for installation of the alkenal [30]. In this work, introduction of the reactive alkenal was followed immediately by an auxiliary directed aldol reaction yielding a bis-allylic alcohol, a sensitive functional group that must be protected and carried through the remainder of the synthesis. The methodology thus requires that the alkenal and subsequent manipulation be carried out ‘late’ in an overall sequence, a situation that limits the versatility of homologation reagents **4**–**6**. In this communication, we show that the salt **7**, now known by the acronym DualPhos [22], is a highly effective, versatile reagent for the homologation process using enolizable and sensitive chiral aldehydes under non-aqueous conditions. A range of synthetic transformations exemplifying the unprecedented stability and hence chemical versatility of latent alkenals derived from DualPhos and a novel chemoselective protocol for generating the free alkenals as required is also reported.

## 2. Results and discussion

We began by screening solvent, base and temperature employing salt **7** and *p*-chlorobenzaldehyde **8** to find the optimal conditions for olefination under non-aqueous conditions. Initial experimentation revealed that NaH and KO*^t^*Bu were both suitable bases and that the addition of a small amount of DMF (THF : DMF ratio 80 : 20 v/v) helped to solubilize the phosphonium salt, which improved the efficiency of ylide formation prior to addition of the aldehyde. The protected alkenal **9** was isolated in 88% yield as essentially the single (*E*)-stereoisomer (entry 1). With these optimized conditions on hand, we set about screening sensitive enolizable and α-chiral aldehydes. Reaction of dihydrocinnamaldehyde **10** was well tolerated (entry 2) yielding the latent alkenal in 79% yield. We previously showed that cinnamaldehyde derivatives could be converted to latent dienals under the original aqueous conditions [22]. We were delighted to find that the enolizable γ-methyl and methylene-containing α,β-unsaturated aldehyde citral **12** yielded the latent dienal **13** in good yield (entry 3). Olefination of the sensitive, chiral pool derived aldehydes **14a**, **14b**, **16** and **18** with a slight excess (1.6 equiv) of DualPhos **7** under these non-aqueous conditions afforded the unsaturated products **15a**, **15b**, **17** and **19** in high yield and good (*E*)-stereoselectivity (entries 4–7). In all cases, the water-soluble tripropylphosphine oxide was easily removed from the resulting latent alkenal and these products all proved stable and were purified by column chromatography to establish the isolated yields as shown (table 1). Thus, it was established that DualPhos is a general reagent for aldehyde to latent alkenal homologation with various aldehydes under aqueous conditions [22], and sensitive enolizable aldehydes under these newly developed non-aqueous conditions.

**Table 1.** Homologation of sensitive aldehydes using DualPhos **7** under non-aqueous conditions.

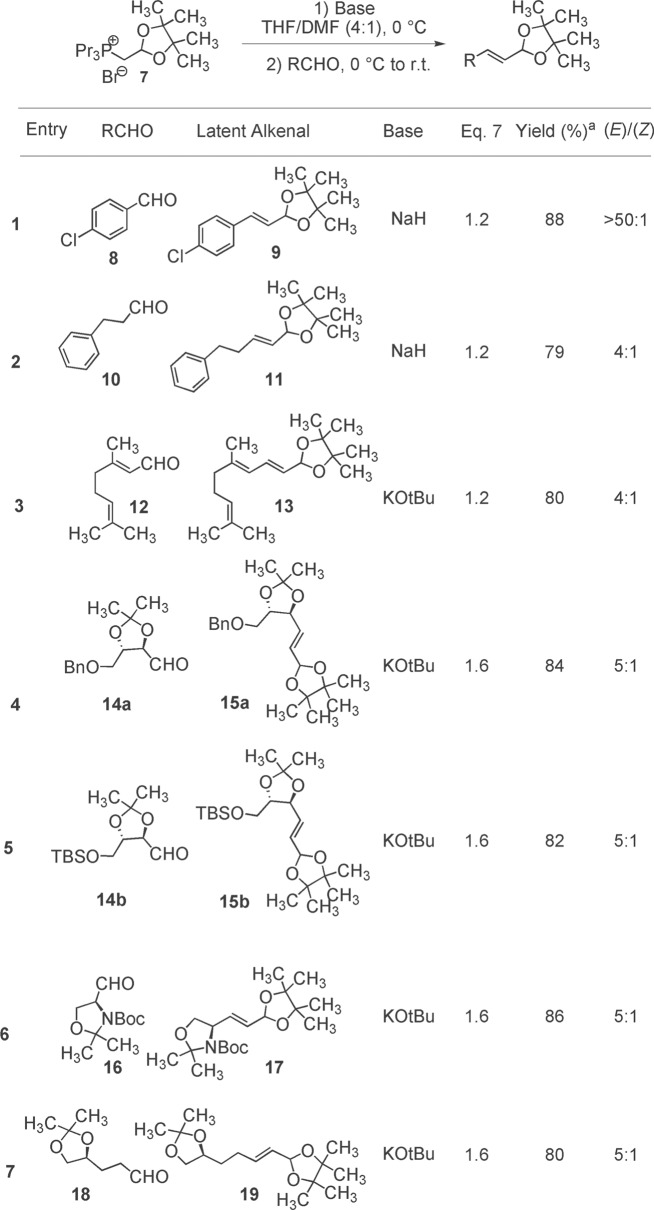

^a^isolated yields of the chromatographically pure latent alkenals.

Deprotection of the latent alkenals to yield α,β-unsaturated aldehydes was previously accomplished using either dilute phosphoric acid or Amberlite IR120 resin (acid form) [22], under which conditions intermediates **9** and **11** yielded their free alkenals in 95% and 82% isolated yields, respectively. While initial attempts to cleave intermediate **13** using IR120 were positive, cleavage of acetonide-containing intermediates such as **15a**, **17** and **19** resulted in significant degradation.

A wide variety of methods have been developed for acetal cleavage [31,32] and we initially surveyed the cleavage of the latent alkenal **19** with a variety of Brønsted acids (i.e. AcOH, CSA, TFA, PTSA, MeSO_3_H and 85% H_3_PO_4_) with marginal success.

**Scheme 1.**
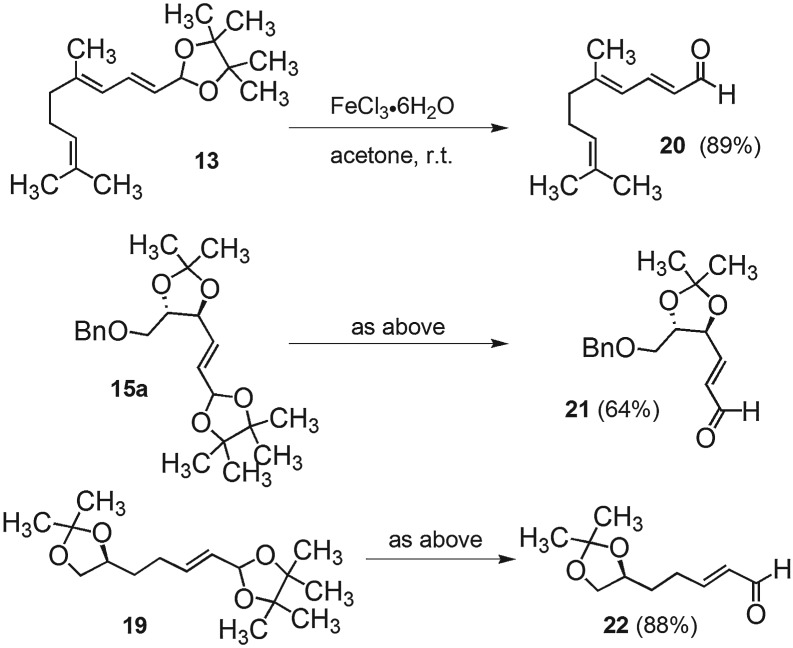
Deprotection of latent alkenals using the newly discovered iron (III) chloride catalysed protocol.

Milder protocols have also been reported using catalytic amounts of Lewis acids and even non-acidic reagents [33–42]. Attempted deprotection of **19** with a range of strong and weak Lewis acids also failed (BF_3_ or AlCl_3_: decomposition, MgBr_2_·Et_2_O, ZnBr_2_, CuI_2_, CuOAc_2_, Yb(OTf)_3_: no reaction). After many such attempts, we discovered that a solution of FeCl_3_·6H_2_O in acetone proved to be the Goldilocks reagent of intermediate reactivity. Under these conditions, ferric chloride hexahydrate dissolves completely in dry acetone to yield a yellow solution of the Lewis acid that effected chemoselective deprotection of **19** within 3 h at room temperature, a protocol that proved to be general (scheme 1).

**Scheme 2.**
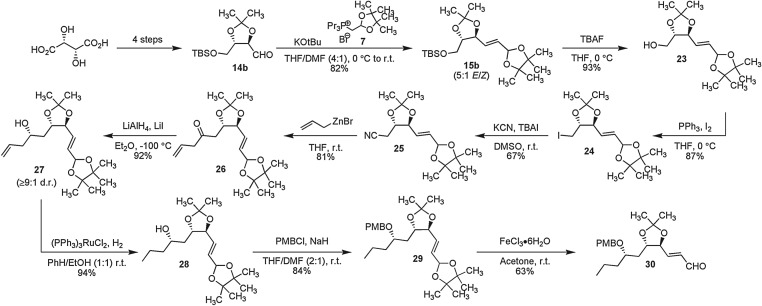
Introduction and diverse chemoselective manipulations effected in the presence of the latent alkenal.

Having developed conditions for the introduction of the latent alkenal functionality using DualPhos under both aqueous [22] and now non-aqueous conditions and a novel method for chemoselective cleavage, we now wished to probe the *stability* of the protected alkenal under a variety of conditions. Of general synthetic concern is the requirement of late introduction of a sensitive alkenal and/or product thereof (allylic alcohol) in a complex synthesis. In order to probe this reactivity, a total synthesis of the nonenolide natural product phomolide G [43–45] was undertaken from l-tartaric acid (scheme 2) involving *early* introduction of the latent alkenal. Phomolide G **38**, isolated recently from an endophytic fungus [43], is a member of an expanding family of 10-member macrolactones that exhibit diverse biological activities [44,45]. Tartaric acid was converted to the known aldehyde **14b** in four steps and 58% overall yield [46,47]. Homologation with DualPhos **7** worked equally well employing NaH or KOtBu as discussed (table 1, entry 5) yielding **15b**. With the latent alkenal installed, removal of the silyl-protecting group was carried out with TBAF giving **22** followed by an Appel reaction [48] to form the primary iodide **24**, nucleophilic substitution of which with KCN in DMSO gave the nitrile **25**.

With nitrile **25** in hand, we began to construct the side-chain and contiguous chiral secondary alcohol. Reaction of nitrile **26** with allylzinc bromide [49] allowed introduction of the required three-carbon unit retaining the olefin handle. The propensity of the resulting allyl ketone **26** to isomerization necessitated the immediate reduction of the ketone under chelation controlled conditions. Allyl ketone **26** was thus dissolved in diethyl ether with a 10-fold excess of LiI at −100°C prior to the addition of LAH. The reduction afforded homo-allyl alcohol **27** in excellent yield with good stereoselectivity (more than or equal to 9 : 1 d.r.). Chemoselective reduction of the terminal alkene was next carried out using (PPh_3_)_3_RuCl_2_ (1 mol%) in EtOH : benzene (1 : 1 v/v) under H_2(g)_ (1 atm) [50] to give alcohol **28** which was subsequently protected as the PMB-ether **29**. Finally, cleavage of the latent alkenal to form **30** was accomplished in a highly chemoselective manner using FeCl_3_·6H_2_O in acetone as described (*vide supra*).

The chemical stability of the latent alkenal carried through this multitude of reactions (scheme 2) is quite remarkable. Compound **15b** contains sensitive protected bis-allylic alcohol and acetal functionalities, yet survives intact through a range of nucleophilic displacements (fluoride, iodide and cyanide), dipolar media, oxidants (I_2_), reductions (including a catalytic hydrogenation and reaction with LiAlH_4_), organometallic reagents and reaction with strong base. These results advocate the high synthetic potential of DualPhos as a reagent for the early introduction of latent alkenals, contributing a novel, versatile strategy toward the design of synthetic sequences.

**Scheme 3.**
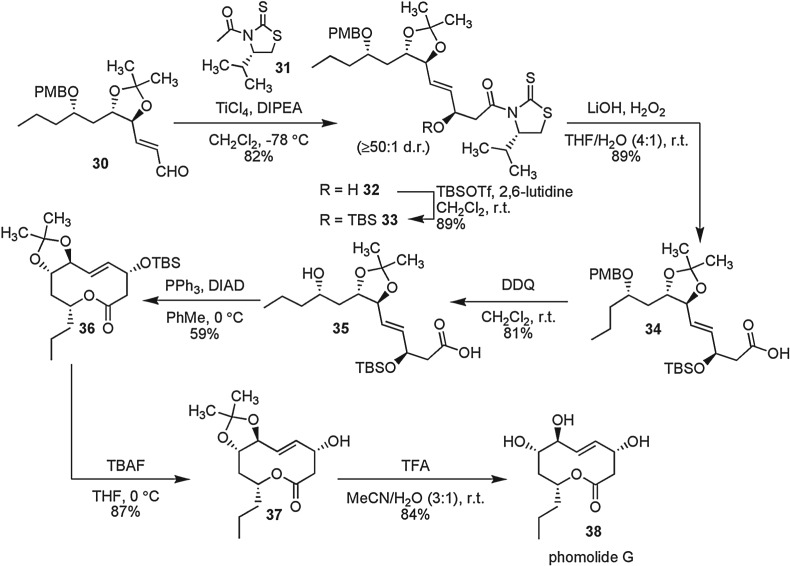
Completion of the synthesis of phomolide G.

The synthesis of phomolide G was completed as shown in scheme 3. A TiCl_4_-mediated [51] Nagao acetate aldol reaction [52] employing thiazolidinethione (+)-**31** on the alkenal **30** gave allylic alcohol **32** in 82% yield (more than or equal to 50 : 1 d.r.). Alcohol **31** was subsequently protected (TBSOTf, 2,6-lutidine in CH_2_Cl_2_ at 0°C) and the auxiliary imide was oxidatively cleaved to the corresponding carboxylic acid. Treatment of acid **34** with DDQ in CH_2_Cl_2_ allowed selective deprotection of the methoxybenzyl ether to give seco acid **35**. This compound was subjected to a standard Mitsunobu reaction [53–56] employing DIAD to yield the macrolactone **36**. Stepwise removal of the protecting groups was achieved by removal of the TBS group using TBAF to give **37**, followed by acidic hydrolysis using TFA in wet-acetonitrile to afford phomolide G **38** identical in all respects to that of the natural [43] corrected [30] structure.

## 3. Conclusion

In conclusion, we have demonstrated the use of DualPhos **7** as a general reagent for two-carbon homologation of aldehydes leading directly to latent (*E*)-alkenals. Olefination of sensitive, enolizable aldehydes can be conducted efficiently using **7** under non-aqueous conditions. A novel method for the cleavage of the protected latent alkenals using iron (III) chloride has also been developed. The intermediate latent alkenals may be cleaved immediately, revealing synthetically useful α,β-unsaturated aldehydes, or may be carried through a wide range of synthetic transformations intact. The robust nature of these latent alkenals contrasts sharply with dialkyl- and cyclic acetals obtained using standard reagents such as **4** and **5** [57,58], and permits a multitude of chemoselective transformations demonstrated in a new synthesis of the natural product macrolactone phomolide G. Further developments on the use of DualPhos and applications of the readily available alkenals in organocatalytic cascade sequences [59,60] are under active investigation in our laboratories.

## Supplementary Material

Supporting Information

## Data accessibility

The detailed experimental synthetic procedures and spectra of the final compounds are provided in the electronic supplementary material.
