# Supplementary material for: DualPhos: a versatile, chemoselective reagent for two-carbon aldehyde to latent (E)-alkenal homologation and application in the total synthesis of phomolide G
Source: R Soc Open Sci. 2016 Nov 23;3(11):160374. doi: 10.1098/rsos.160374 (PMC5180113; doi:10.1098/rsos.160374)
Supplement: Supporting Information [file rsos160374supp1.pdf]

Introduction of DualPhos as a versatile, chemoselective reagent for two-carbon aldehyde to latent (*E*)-alkenal homologation: Application in the total synthesis of Phomolide G

*David McLeod, James McNulty**

Department of Chemistry & Chemical Biology, McMaster University,

1280 Main Street West, Hamilton, Ontario, L8S 4M1, Canada

Supporting Information

Supporting Information

Part 1: Experimental Procedures and Characterization Data

Table of Contents

General Experimental... page SI-4
Scheme 1 .... page SI-5
Scheme 2 .... page SI-6
Scheme 3... page SI-7
Scheme 4... page SI-8
Scheme 5... page SI-9
Experimental Procedures and Characterization Data.. page SI-10~36
Compound **9**.. page SI-10
Compound **11**.. page SI-10
Compound **13**.. page SI-11
Compound **15a**.. page SI-12
Compound **17**.. page SI-14
Compound **19**.. page SI-15
Compound **20**.. page SI-16
Compound **21**.. page SI-16
Compound **22**.. page SI-17
Compound **15b**.. page SI-18
Compound **23**.. page SI-20
Compound **24**.. page SI-21
Compound **25**.. page SI-22
Compound **26**.. page SI-23
Compound **27**.. page SI-24
Compound **28**.. page SI-26
Compound **29**.. page SI-27
Compound **30**.. page SI-28
Compound **32**.. page SI-29
Compound **33**.. page SI-30
Compound **34**.. page SI-31
Compound **35**.. page SI-32
Compound **36**.. page SI-33

Compound **37**.. page SI-34
Compound **38**.. page SI-35
References.. page SI-36

General Considerations. Reactions were carried out under nitrogen or argon atmosphere with dry solvents using anhydrous conditions unless otherwise stated. Dry diethyl ether (Et_2O), tetrahydrofuran (THF), Toluene (PhMe) and triethylamine and N,N-diisopropylethylamine were distilled from Na^0 with benzophenone as an indicator. Dry dichloromethane (CH_2Cl_2) was distilled from calcium hydride while dry methanol (MeOH) was distilled from Mg^0. All fine chemicals were obtained from Sigma-Aldrich and used without further purification unless otherwise stated. Yields refer to chromatographically and spectroscopically (^{1}H-NMR) homogeneous materials, unless otherwise stated. CIMS were run on a Micromass Quattro Ultima spectrometer fitted with a direct injection probe (DIP) with ionization energy set at 70 eV. HRMS (EI) were performed with a Micromass Q-Tof Ultima spectrometer. NMR spectra were recorded on Bruker AV-600 and AV-700 spectrometers and calibrated using residual undeuterated solvent as an internal reference($CHCl_3$ @ δ 7.26 ppm ^{1}H NMR, δ 77.16 ppm ^{13}C NMR; Acetone-d_6 @ δ 2.05, ^{1}H NMR, δ 29.84 ppm ^{13}C NMR); ^{31}P spectra were calibrated using an external reference of 85% H_3PO_4. Signal assignments were accomplished *via* analysis of HMBC, HMQC, COSY, NOESY experiments where necessary. The (*E*) to (*Z*) ratios were determined from the relative integration of the ^{1}H spectra for the olefinic protons. All melting points are corrected. Preparative procedures and experimental data for compounds **31**[1], **7**[2], **SI-1**[3], **14a**[4], **SI-2**[5], **14b**[5], **16**[6] and **18**[7] have been previously reported.

Scheme 1. Initial Wittig reactions

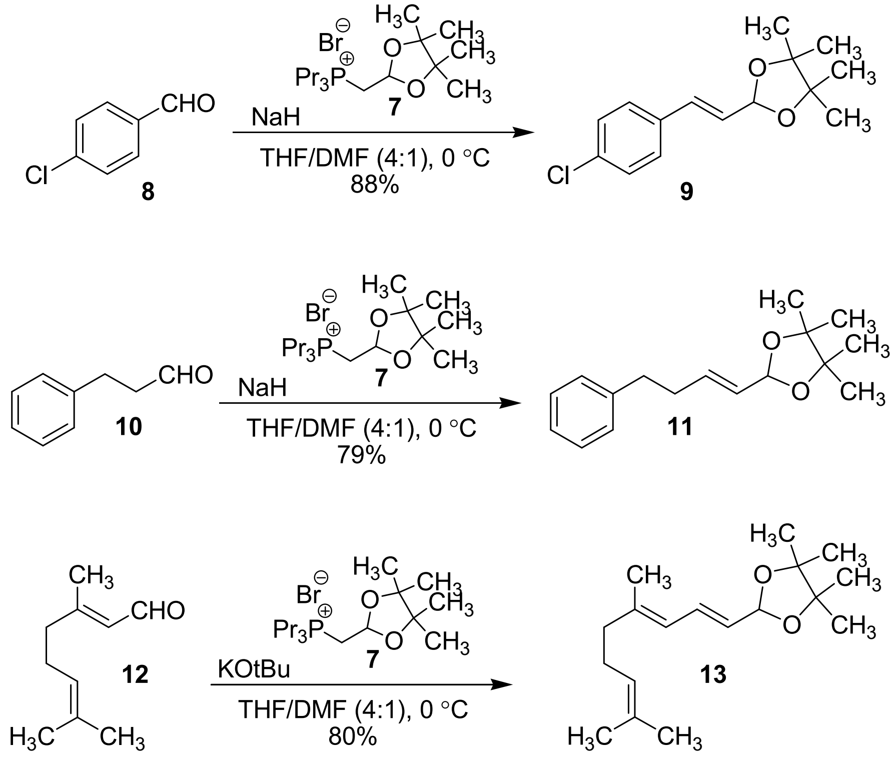

Scheme 2. Homologation of chiral aldehydes **14a, 16** and **18**

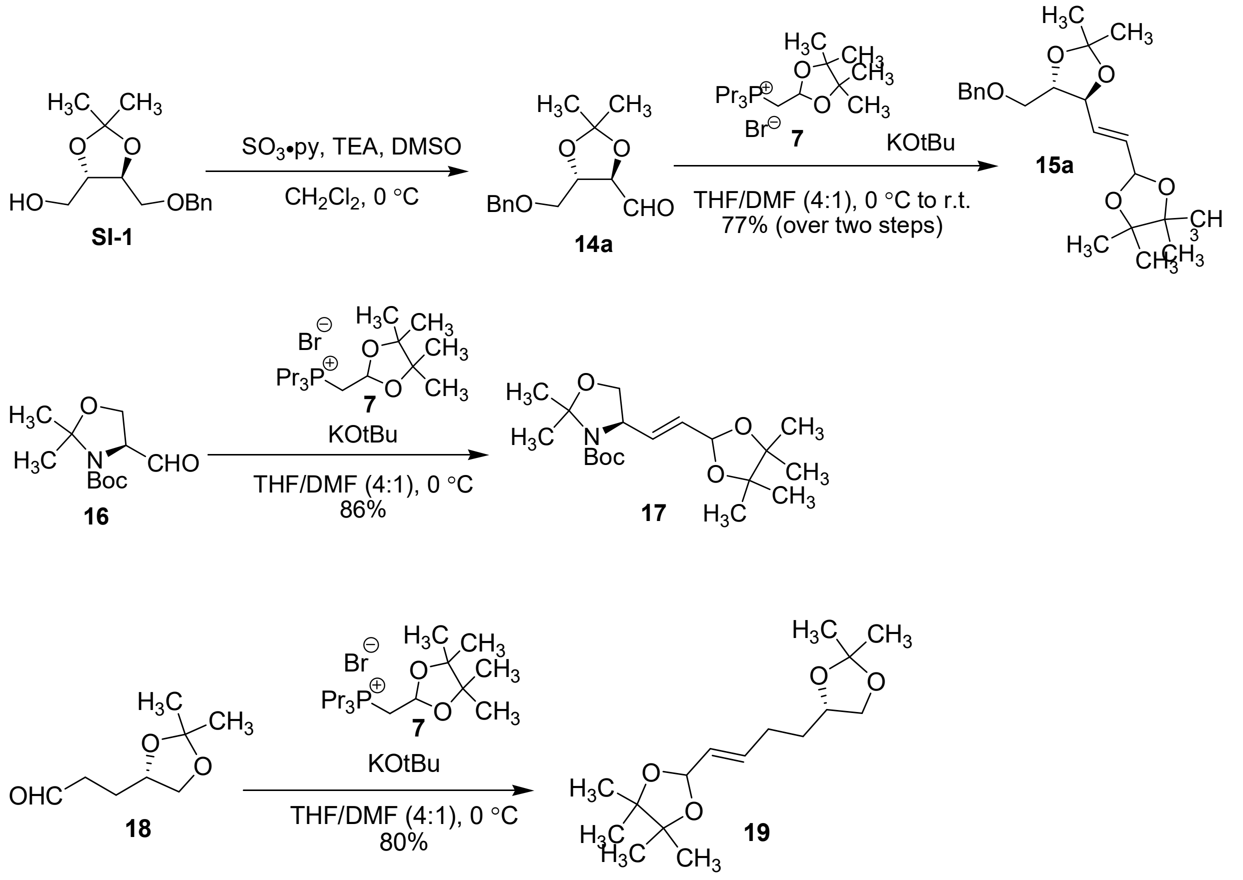

Scheme 3. Chemoselective deprotection of **13**, **15a** & **19**

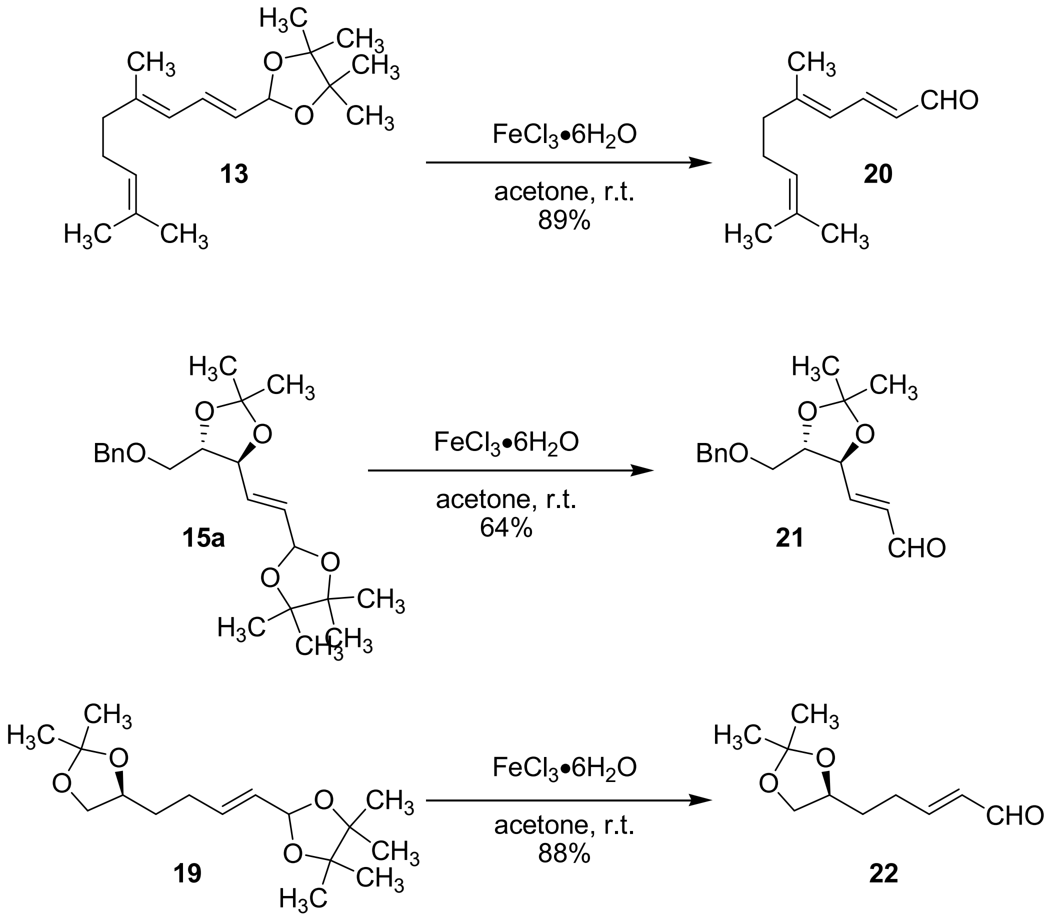

Scheme 4. Synthesis of **30**

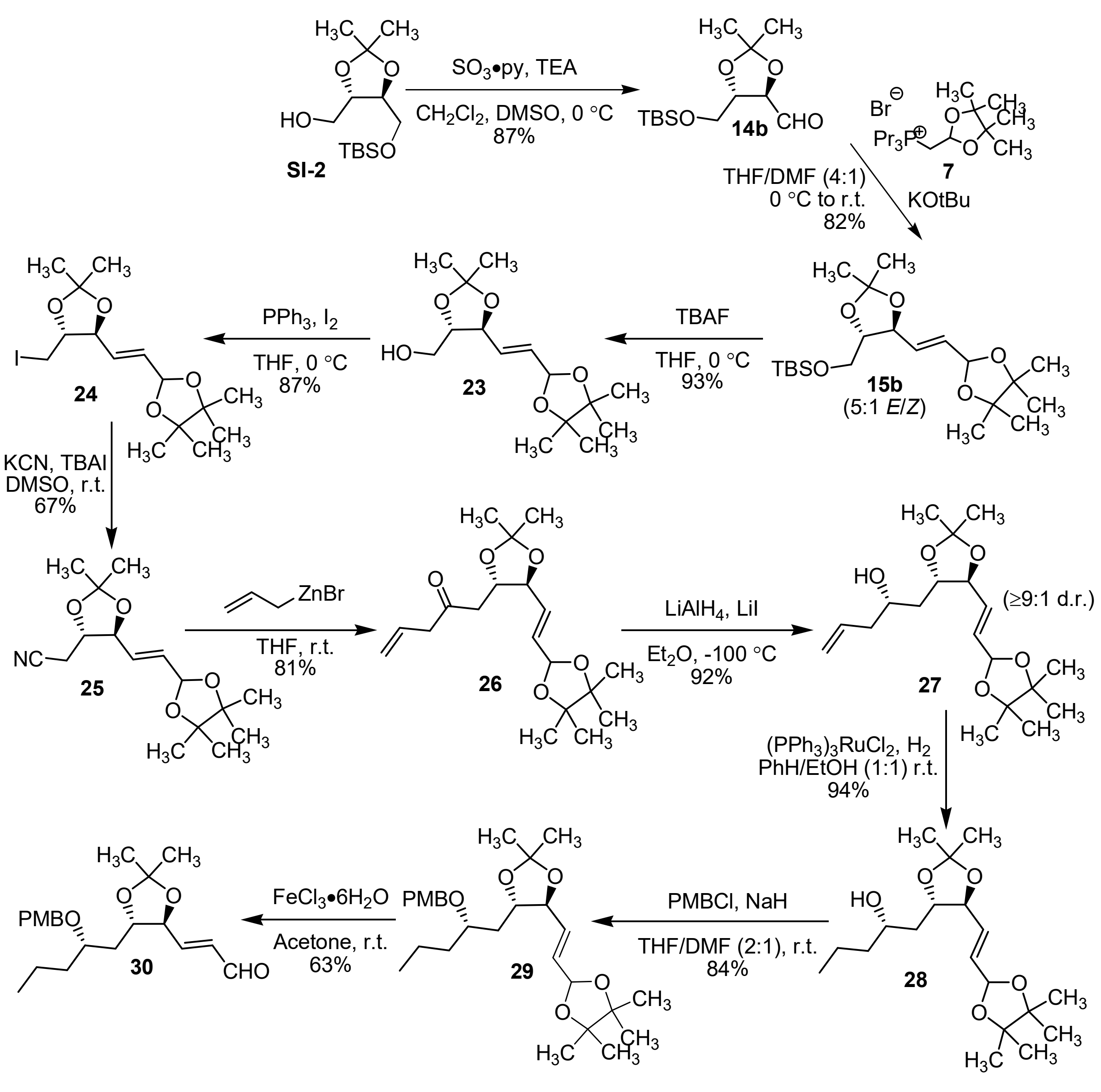

Scheme 5. Synthesis of **38**

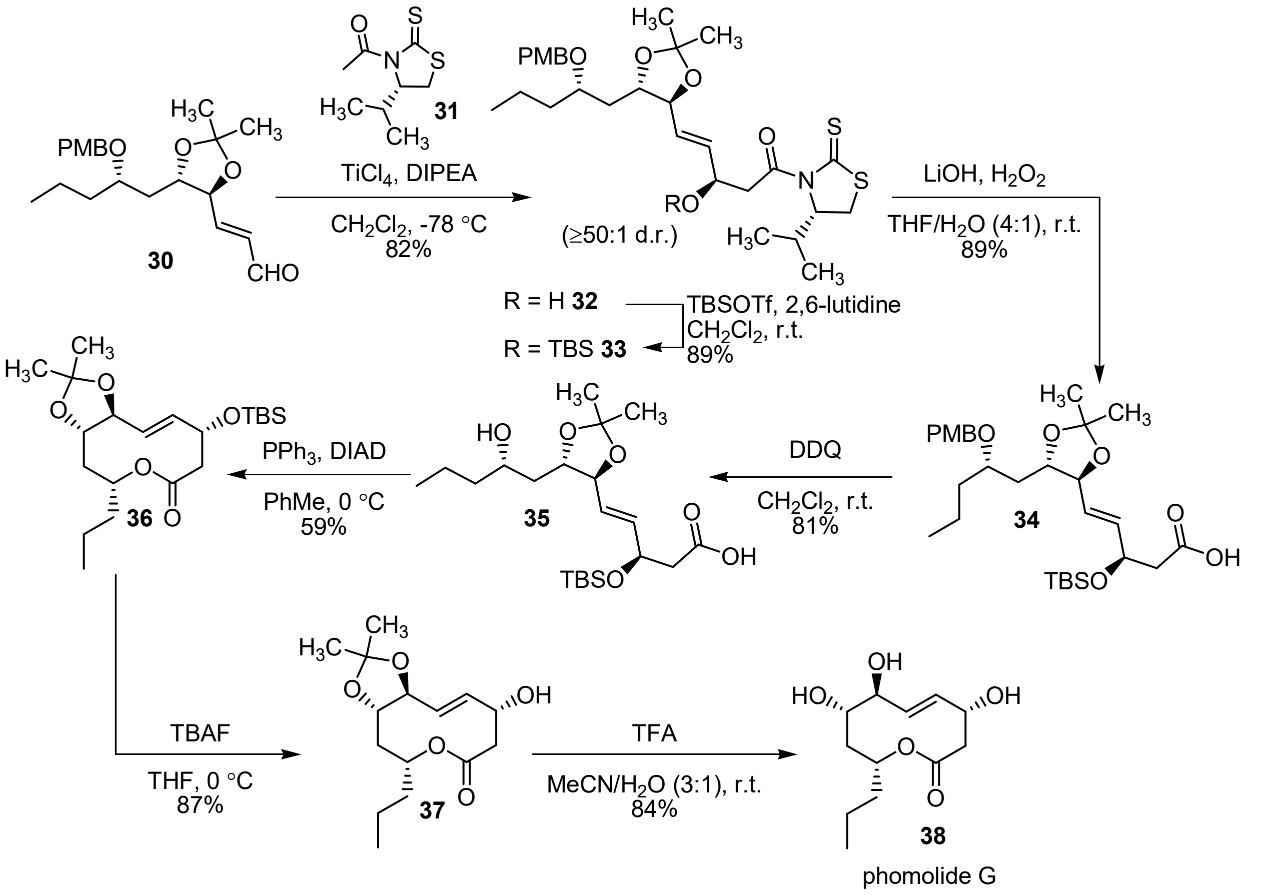

Experimental Procedures

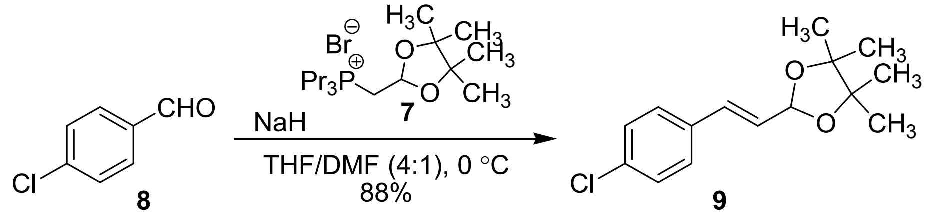

Synthesis of **9**: To phosphonium salt **7** (260 mg, 0.68 mmol) in THF (0.8 mL) and DMF (0.2 mL) at 0 °C was added KO^{t}Bu (76 mg, 0.68 mmol) to afford a light yellow mixture. The reaction was left to stir at 0 °C for 1 h before 4-chlorobenzaldehyde **8** (80 mg, 0.57 mmol) was added in one portion as a solid. The reaction mixture was allowed to warm to room temperature overnight before being quenched with a saturated NH_4Cl solution (10 mL). Standard extractive work-up with diethyl ether (3 x 10 mL) was followed by washing the combined organic fractions with brine (10 mL). The combined organic extracts were then dried over anhydrous $MgSO_4$ and concentrated under reduced pressure to give crude **9** which was chromatographed (silica gel, EtOAc:hexanes, 1:19 → 1:9 v/v) to give pure **9** (132 mg, 88%) as a light yellow oil.

Physical State: pale yellow oil

R_f = 0.40 (EtOAc/hexanes, 1:9 v/v; $KMnO_4$)

^{1}H NMR [$CDCl_3$, 600 MHz] δ: 7.15 (d, J = 8.5 Hz, 2H), 7.10 (d, J = 8.5 Hz, 2H), 6.49 (d, J = 15.9 Hz, 1H), 5.95 (dd, J = 15.9, 6.6 Hz, 1H), 5.34 (d, J = 6.6 Hz, 1H), 1.10 (s, 6H), 1.09 (s, 6H)

^{13}C NMR [$CDCl_3$, 150 MHz] δ: 134.6, 133.8, 133.0, 128.7, 128.4, 128.1, 100.4, 82.4, 24.0, 22.0.

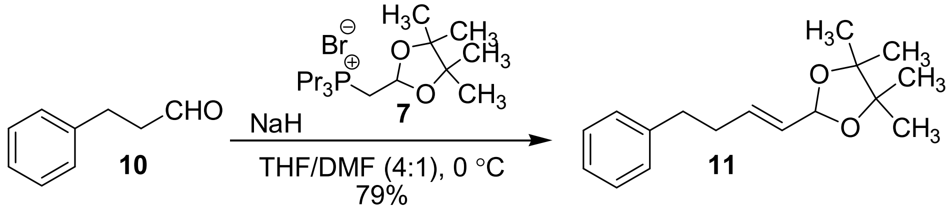

Synthesis of **11**: To phosphonium salt **7** (305 mg, 0.80 mmol) in THF (0.8 mL) and DMF (0.2 mL) at 0 °C was added KO^{t}Bu (89 mg, 0.80 mmol) to afford a light yellow mixture. The reaction was left to stir at 0 °C for 1 h before dihydrocinnamaldehyde **10** (89 mg, 0.66 mmol) was added

in one portion as a solid. The reaction mixture was allowed to warm to room temperature overnight before being quenched with a saturated NH_4Cl solution (10 mL). Standard extractive work-up with diethyl ether (3 x 10 mL) was followed by washing the combined organic fractions with brine (10 mL). The combined organic extracts were then dried over anhydrous $MgSO_4$ and concentrated under reduced pressure to give crude **11** which was chromatographed (silica gel, EtOAc:hexanes, 1:19 → 1:9 v/v) to give pure **11** (136 mg, 79%) as a light yellow oil.

Physical State: pale yellow oil

R_f = 0.68 (EtOAc/hexanes, 1:4 v/v; $KMnO_4$)

^{1}H NMR [$CDCl_3$, 600 MHz] δ: 7.17 (dt, J = 11.2, 3.7 Hz, 2H), 7.09 (dd, J = 12.6, 5.4 Hz, 3H), 5.82 (dt, J = 15.3, 6.6 Hz, 1H), 5.45 (dd, J = 15.4, 7.2 Hz, 1H), 5.23 (d, J = 7.2 Hz, 1H), 2.63 – 2.60 (m, 2H), 2.29 (td, J = 7.8, 1.1 Hz, 2H), 1.13 (s, 6H), 1.12 (s, 6H).

^{13}C NMR [$CDCl_3$, 150 MHz] δ: 141.7, 136.1, 129.3, 128.4, 125.9, 100.9, 82.1, 35.2, 33.9, 24.0, 22.0.

HREI MS (*m*/*z*): $(M)^+$ calcd. for $C_{17}H_{24}O_2$, 260.1776; found 260.1776

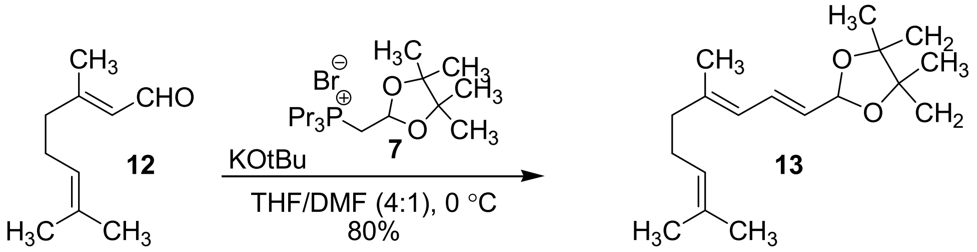

Synthesis of **13**: To phosphonium salt **7** (380 mg, 0.99 mmol) in THF (0.8 mL) and DMF (0.2 mL) at 0 °C was added KO^{t}Bu (110 mg, 0.99 mmol) to afford a light yellow mixture. The reaction was left to stir at 0 °C for 1 h before citral **12** (126 mg, 83 mmol, 6:4 *E*/*Z*) was added in one portion as a solid. The reaction mixture was allowed to warm to room temperature overnight before being quenched with a saturated NH_4Cl solution (10 mL). Standard extractive work-up with hexanes (3 x 10 mL) was followed by washing the combined organic fractions with brine (10 mL). The combined organic extracts were then dried over anhydrous $MgSO_4$ and concentrated under reduced pressure to give crude **13** which was chromatographed (silica gel,

EtOAc:hexanes, 1:49 → 1:19 v/v) to give pure **13** (183 mg, 80%, mixture of isomers, 4:1 *E*/*Z* ratio on the formed alkene) as a light yellow oil.

Physical State: colourless oil

***R**$_f$* = 0.39 (EtOAc/hexanes, 1:9 v/v; $KMnO_4$)

Spectroscopic data for (2E, 4E)**-13**

^{1}H NMR [$CDCl_3$, 600 MHz] δ: 6.59 (dd, *J* = 15.1, 10.9 Hz, 1H), 5.88 (d, *J* = 11.0 Hz, 1H), 5.54 (dd, *J* = 15.0, 7.1 Hz, 1H), 5.44 (d, *J* = 7.3 Hz, 1H), 5.14 – 5.07 (m, 1H), 2.15 – 2.07 (m, 4H), 1.76 (s, 3H), 1.69 (s, 3H), 1.61 (s, 3H), 1.25 (s, 12H).

^{13}C NMR [$CDCl_3$, 150 MHz] δ: 141.5, 131.7, 130.7, 128.2, 123.8, 123. 6, 101.0, 82.1, 39.9, 26.8, 25.7, 24.0, 23. 9, 22.0, 17.7, 16.7

Spectroscopic data for (2E, 4Z)**-13**

^{1}H NMR [$CDCl_3$, 600 MHz] δ: 6.59 (dd, *J* = 15.1, 10.9 Hz, 1H), 5.88 (d, *J* = 11.0 Hz, 1H), 5.54 (dd, *J* = 15.0, 7.1 Hz, 1H), 5.44 (d, *J* = 7.3 Hz, 1H), 5.14 – 5.07 (m, 1H), 2.15 – 2.07 (m, 4H), 1.82 (s, 3H), 1.69 (s, 3H), 1.61 (s, 3H), 1.25 (s, 12H).

^{13}C NMR [$CDCl_3$, 150 MHz] δ: 141.8, 132.0, 131.2, 128.3, 124.3, 123.8, 101.0, 82.1, 32.5, 26.4, 25.7, 24.1, 24.0, 22.0, 17.7

HREI MS (*m*/*z*): $(M)^+$ calcd. for $C_{18}H_{30}O_2$, 278.2246; found 278.2239.

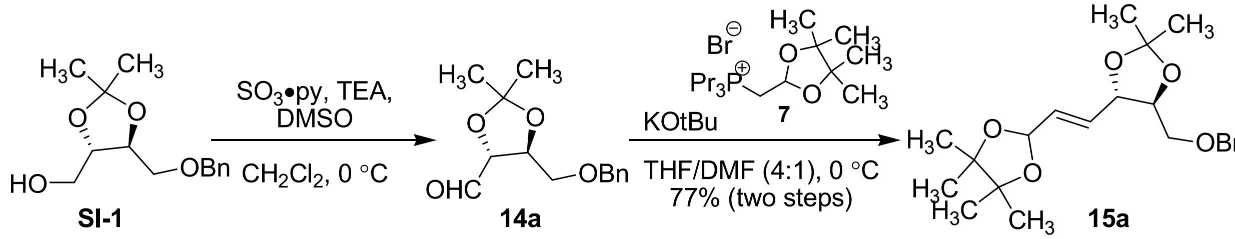

Synthesis of **15a**: To **SI-1** (1.01 g, 4.00 mmol) in CH_2Cl_2 (24 mL) at room temperature were added DMSO (2.0 mL) and TEA (6.0 mL). The solution was then cooled to 0 °C prior to the addition of a solution of SO_3·py (2.55 g, 16.0 mmol) in DMSO (8 mL) dropwise over 20 minutes and stirring continued for 4 h at this temperature. The reaction was quenched with a saturated

aqueous solution of NH_4Cl (50 mL) prior to extraction with Et_2O (3 x 50 mL). The combined organic fractions were dried over anhydrous $MgSO_4$, filtered, and concentrated under reduced pressure to afford a yellow oil. The crude aldehyde **14a** was placed under high vacuum for 2 h and carried on directly to the next step.

To phosphonium salt **7** (2.45 g, 6.40 mmol) was added THF (5.1 mL) and DMF (1.3 mL). The solution was cooled to 0 °C for 15 minutes prior to the addition of KO^tBu (718 mg, 6.40 mmol) portion-wise to afford a light yellow mixture. The mixture containing the base was left to stir at 0 °C for 1 h before the corresponding aldehyde **14a** in THF (3.0 mL) was subsequently added dropwise. The reaction mixture was allowed to warm to room temperature overnight before being quenched with a saturated NH_4Cl solution (20 mL). Standard extractive work-up with diethyl ether (3 x 20 mL) was followed by washing the combined organic fractions with brine (10 mL). The combined organic extracts were then dried over anhydrous $MgSO_4$ and concentrated under reduced pressure to give crude **15a** which was chromatographed (silica gel, EtOAc:hexanes, 1:19 → 1:9 v/v) to give pure **15a** (1.16 g, 77% two steps, 5:1 *E*/*Z*) as a light yellow oil.

Physical State: colourless oil

$\mathbf{R_f}$ = 0.25 (EtOAc:hexanes, 1:4 v/v; vanillin)

$[\alpha]_D$= −11.9° ($CHCl_3$, *c* 1.05)

^{1}H-NMR [$CDCl_3$, 600 MHz] δ: 7.29 – 7.24 (m, 5H), 5.80 (dd, *J* = 15.6, 6.3 Hz, 1H), 5.70 (ddd, *J* = 15.5, 6.1, 0.9 Hz, 1H), 5.30 (d, *J* = 6.1 Hz, 1H), 4.54 – 4.50 (m, 2H), 4.22 (ddd, *J* = 8.2, 6.3, 0.8 Hz, 1H), 3.88 – 3.82 (m, 1H), 3.52 (qd, *J* = 10.5, 4.6 Hz, 2H), 1.36 (s, 3H), 1.36 (s, 3H), 1.14 (s, 6H), 1.13 (s, 3H), 1.13 (s, 3H).

^{13}C-NMR [$CDCl_3$, 150 MHz] δ: 138.0, 132.2, 131.9, 128.4, 127.7, 127.7, 109.6, 99.5, 82.3, 80.0, 77.7, 73.6, 69.5, 27.0, 26.9, 23.9, 23.9, 22.0.

HRES MS (*m*/*z*): $(M+H)^+$ calcd. for $C_{22}H_{33}O_5$, 377.2328; found 377.2318.

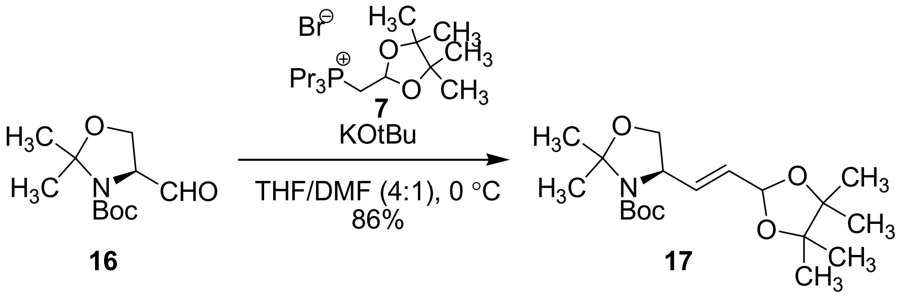

Synthesis of **17**: To phosphonium salt **7** (619 mg, 1.61 mmol) was added THF (1.6 mL) and DMF (0.4 mL). The solution was cooled to 0 °C for 15 minutes prior to the addition of KO^{t}Bu (182 mg, 1.61 mmol) portion-wise to afford a light yellow mixture. Slow addition of the base is required to prevent local heating which causes degradation of the phosphonium salt. The mixture containing the base was left to stir at 0 °C for 1 h before the Garner's aldehyde **16** (231 mg, 1.01 mmol) was subsequently added dropwise. The reaction mixture was allowed to warm to room temperature overnight before being quenched with a saturated NH_4Cl solution (10 mL). Standard extractive work-up with diethyl ether (3 x 5 mL) was followed by washing the combined organic fractions with brine (10 mL). The combined organic extracts were then dried over anhydrous $MgSO_4$ and concentrated under reduced pressure to give crude **17** which was flash chromatographed (silica gel, EtOAc:hexanes, 1:19 → 1:9 v/v) to give pure **17** (309 mg, 86%) as a colourless oil.

Physical State: colourless oil

$\boldsymbol{R_f}$ = 0.32 (EtOAc:hexanes, 1:4 v/v)

[α]$_D$= +6.8° ($CHCl_3$, *c* 1.15)

^{1}H NMR [Tol-d_8, 500 MHz, 70 °C] δ: 5.80 (dd, *J* = 15.3, 7.2 Hz, 1H), 5.72 (dd, *J* = 15.3, 4.5 Hz, 1H), 5.40 (d, *J* = 5.5 Hz, 1H), 4.16 (s, 1H), 3.72 (dd, *J* = 8.6, 6.3 Hz, 1H), 3.51 (dd, *J* = 8.8, 2.1 Hz, 1H), 1.69 (s, 3H), 1.54 (s, 3H), 1.47 (s, 9H), 1.13 (s, 6H), 1.09 (d, *J* = 2.0 Hz, 6H).

^{13}C NMR [Tol-d_8, 500 MHz, 70 °C] δ: 152.2, 133.7, 132.3, 100.5, 94.5, 82.3, 79.7, 68.5, 59.2, 28.8, 27.3, 24.4, 24.3, 22.4.

HREI MS (*m/z*): $(M–CH_3)^+$ calcd. for $C_{18}H_{30}NO_5$, 340.2124; found 340.2133.

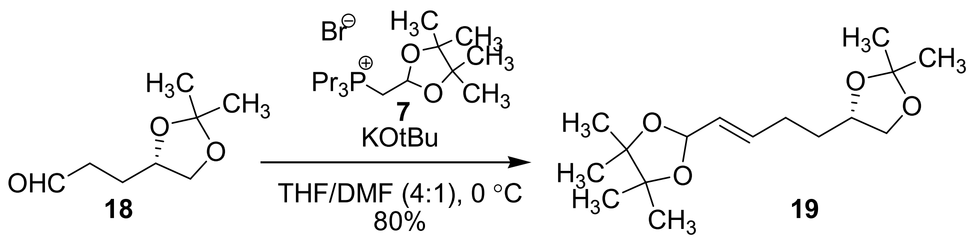

Synthesis of **19**: To phosphonium salt **7** (810 mg, 2.11 mmol) was added THF (0.8 mL) and DMF (0.2 mL). The solution was cooled to 0 °C for 15 minutes prior to the addition of KO^tBu (237 mg, 2.11 mmol) portion-wise to afford a light yellow mixture. Slow addition of the base is required to prevent local heating which causes degradation of the phosphonium salt. The mixture containing the base was left to stir at 0 °C for 1 h before the corresponding aldehyde **18** (209 mg, 1.32 mmol) in THF (0.2 mL) was subsequently added dropwise. The reaction mixture was allowed to warm to room temperature overnight before being quenched with a saturated NH_4Cl solution (20 mL). Standard extractive work-up with diethyl ether (3 x 20 mL) was followed by washing the combined organic fractions with brine (10 mL). The combined organic extracts were then dried over anhydrous $MgSO_4$ and concentrated under reduced pressure to give crude **19** which was chromatographed (silica gel, EtOAc:hexanes, 1:19 → 1:9 v/v) to give pure **19** (301 mg, 80%, 5:1 *E*/*Z*) as a colourless oil.

Physical State: colourless oil

$\boldsymbol{R_f}$ = 0.29 (EtOAc:hexanes, 1:9 v/v; vanillin)

$[\alpha]_D$= +9.5° ($CHCl_3$, *c* 0.93)

^{1}H NMR [$CDCl_3$, 600 MHz] δ: 5.88 (dt, *J* = 15.3, 6.6 Hz, 1H), 5.52 (ddt, *J* = 15.4, 7.2, 1.4 Hz, 1H), 5.34 (d, *J* = 7.2 Hz, 1H), 4.10 (ddd, *J* = 12.9, 7.0, 6.0 Hz, 1H), 4.04 (dd, *J* = 7.8, 6.0 Hz, 1H), 3.52 (t, *J* = 7.5 Hz, 1H), 2.26 – 2.18 (m, 1H), 2.16 – 2.08 (m, 1H), 1.75 (dddd, *J* = 12.8, 9.8, 7.0, 5.7 Hz, 1H), 1.62 (ddt, *J* = 13.5, 9.8, 5.8 Hz, 1H), 1.41 (s, 3H), 1.36 (s, 3H), 1.24 (s, 6H), 1.23 (s, 6H).

^{13}C NMR [$CDCl_3$, 150 MHz] δ: 135.8, 129.5, 108.8, 100.8, 82.1, 75.4, 69.3, 32.7, 28.2, 26.9, 25.7, 24.0, 22.0.

HRES MS (*m*/*z*): $(M+H)^+$ calcd. for $C_{16}H_{29}O_4$, 285.2066; found 285.2060.

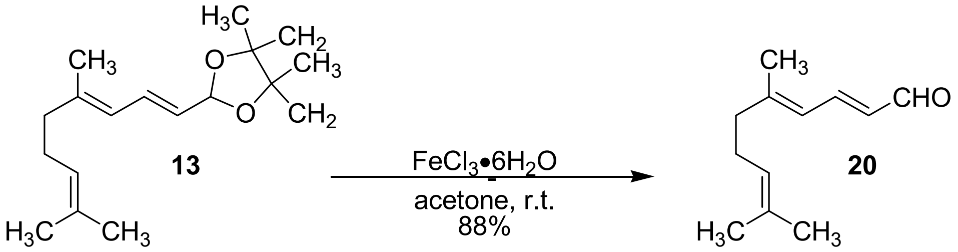

Synthesis of **20**: To **13** in acetone (24 mg, 0.087 mmol) in acetone (2.1 mL) at room temperature was added iron trichloride hexahydrate (1.2 mg, $4.3x10^{-3}$ mmol). The yellow solution was stirred for 16 h, at which point water (5 mL) was added and volatile organic compounds were removed under reduced pressure. The remaining aqueous mixture was extracted with hexanes (3 x 5 mL) and the combined organic extracts were dried over Na_2SO_4 and concentrated under reduced pressure to give **20** (14 mg, 88%, ~7:3 *E*/*Z*) as a yellow oil.

Physical State: yellow oil

$\boldsymbol{R_f}$ = 0.20 (EtOAc/hexanes, 1:9 v/v; 2,4-DNP)

^{1}H NMR [$CDCl_3$, 600 MHz] δ: 9.51 (d, *J* = 8.0 Hz, 1H), 7.33 (dd, *J* = 15.0, 11.5 Hz, 1H), 6.08 (d, *J* = 11.5 Hz, 1H), 6.02 (dd, *J* = 14.9, 8.0 Hz, 1H), 5.01 (t, *J* = 6.1 Hz, 1H), 2.14 – 2.09 (m, 4H), 1.88 (s, 3H), 1.62 (s, 3H), 1.55 (s, 3H)

^{13}C NMR [$CDCl_3$, 150 MHz] δ: 194.1, 153.0, 148.6, 132.6, 130.0, 123.8, 123.1, 40.5, 26.3, 25.7, 17.7, 17.6.

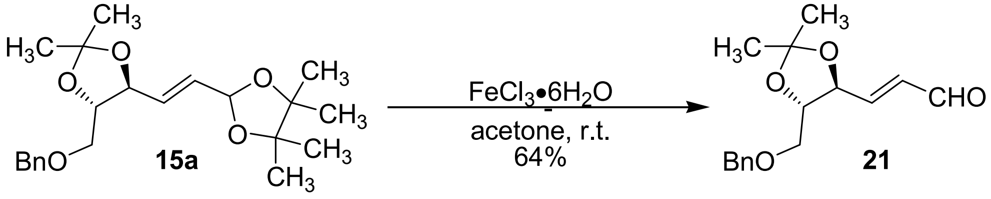

Synthesis of **21**: To **15a** (56 mg, 0.15 mmol) in acetone (5.0 mL) at room temperature was added iron trichloride hexahydrate (2.1 mg, $7.4x10^{-3}$ mmol). The yellow solution was stirred for 18 h, at which point water (5 mL) was added and volatile organic compounds were removed under

reduced pressure. The remaining aqueous mixture was extracted with diethyl ether (3 x 5 mL) and the combined organic extracts were dried over Na_2SO_4, concentrated under reduced pressure and chromatographed (silica gel, EtOAc:hexanes, 1:19 → 1:9) to give **21** (26 mg, 64%) as a colourless oil.

Physical State: colourless oil

***R**$_f$* = 0.21 (EtOAc/hexanes, 1:4 v/v; vanillin)

[α]$_D$= −14.5° ($CHCl_3$, *c* 0.40)

^{1}H NMR [$CDCl_3$, 600 MHz] δ: 9.51 (d, *J* = 7.9 Hz, 1H), 7.31 – 7.26 (m, 2H), 7.26 – 7.23 (m, 3H), 6.71 (dd, *J* = 15.7, 5.0 Hz, 1H), 6.28 (ddd, *J* = 15.7, 7.9, 1.5 Hz, 1H), 4.53 (d, *J* = 5.0 Hz, 2H), 4.49 (ddd, *J* = 8.3, 5.0, 1.5 Hz, 1H), 3.91 (dt, *J* = 8.3, 5.0 Hz, 1H), 3.63 (dd, *J* = 10.2, 4.8 Hz, 1H), 3.56 (dd, *J* = 10.2, 5.2 Hz, 1H), 1.40 (s, 3H), 1.37 (s, 3H).

^{13}C NMR [$CDCl_3$, 150 MHz] δ: 193.1, 152.6, 137.6, 132.3, 128.5, 128.0, 127.8, 110.5, 79.3, 78.0, 74.8, 69.5, 26.9, 26.6.

HRES MS (*m*/*z*): $(M+Na)^+$ calcd. for $C_{16}H_{20}O_4Na$, 299.1259; found 299.1261.

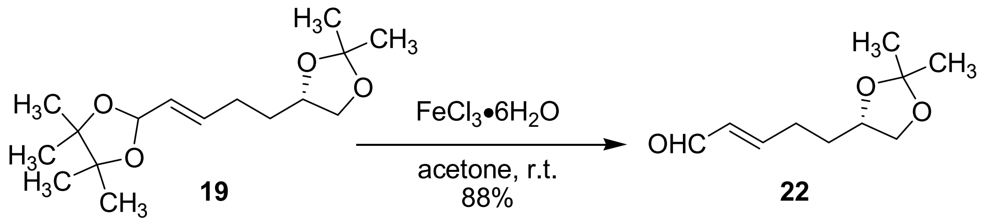

Synthesis of **22**: To **19** (31 mg, 0.11 mmol) in acetone (3.0 mL) at room temperature was added iron trichloride hexahydrate (1.5 mg, $5.5x10^{-3}$ mmol). The yellow solution was stirred for 6 h, at which point water (5 mL) was added and volatile organic compounds were removed under reduced pressure. The remaining aqueous mixture was extracted with hexanes (3 x 5 mL) and the combined organic extracts were dried over Na_2SO_4 and concentrated under reduced pressure to give **22** (17.7 mg, 88%) as a colourless oil.

Physical State: colourless oil

R_f = 0.18 (EtOAc:hexanes, 1:9 v/v; vanillin)

[α]D= –19.0° ($CHCl_3$, *c* 0.12)

^{1}H NMR [$CDCl_3$, 600 MHz] δ: 9.45 (d, *J* = 7.8 Hz, 1H), 6.80 (dt, *J* = 15.6, 6.7 Hz, 1H), 6.08 (ddt, *J* = 15.6, 7.8, 1.5 Hz, 1H), 4.06 (dt, *J* = 7.1, 6.0 Hz, 1H), 4.00 (dd, *J* = 7.9, 6.0 Hz, 1H), 3.49 (dd, *J* = 7.9, 7.0 Hz, 1H), 2.48 – 2.40 (m, 1H), 2.39 – 2.31 (m, 1H), 1.77 – 1.63 (m, 2H), 1.35 (s, 3H), 1.29 (s, 3H).

^{13}C NMR [$CDCl_3$, 150 MHz] δ: 193.9, 157.4, 133.2, 109.1, 75.1, 69.2, 31.8, 29.0, 27.0, 25.6

HRES MS (*m/z*): $(M+H)^+$ calcd. for $C_{10}H_{17}O_3$, 185.1178; found 185.1177.

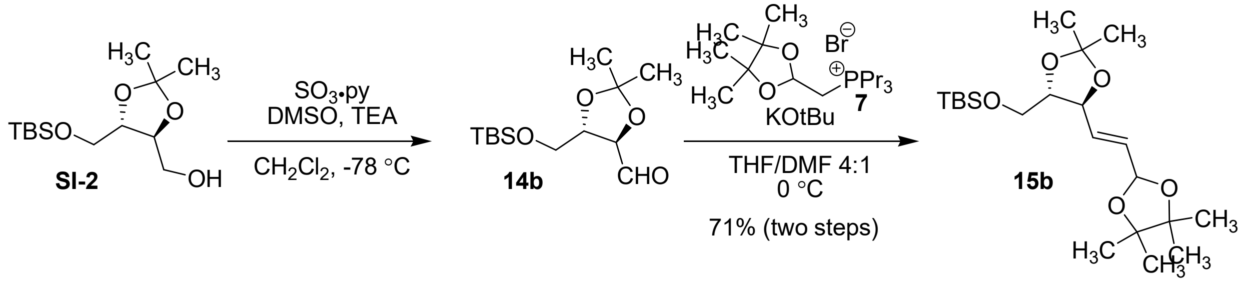

Synthesis of **15b**: To **SI-2** (1.86 g, 6.73 mmol) in CH_2Cl_2 (32 mL) at room temperature were added DMSO (3.0 mL) and TEA (8.1 mL). The solution was then cooled to 0 °C prior to the addition of a solution of SO_3·py (4.28 g, 26.9 mmol) in DMSO (13.0 mL) dropwise over 15 minutes and stirring continued for 4 h at this temperature. The reaction was quenched with a saturated aqueous solution of NH_4Cl (50 mL) prior to extraction with Et_2O (3 x 50 mL). The combined organic fractions were dried over anhydrous $MgSO_4$, filtered, and concentrated under reduced pressure to afford a yellow oil. The crude aldehyde **14b** was placed under high vacuum for 1 h and carried on directly to the next step.

To phosphonium salt **7** (3.73 g, 9.72 mmol) was added THF (8.0 mL) and DMF (2.0 mL). The solution was cooled to 0 °C for 15 minutes prior to the addition of KO^tBu (1.09 g, 9.72 mmol) portion-wise to afford a light yellow mixture. The mixture containing the base was left to stir at 0

°C for 1 h before the corresponding aldehyde **14b** in THF (5.0 mL) was subsequently added dropwise. The reaction mixture was allowed to warm to room temperature overnight before being quenched with a saturated NH_4Cl solution (50 mL). Standard extractive work-up with diethyl ether (4 x 20 mL) was followed by washing the combined organic fractions with brine (10 mL). The combined organic extracts were then dried over anhydrous $MgSO_4$ and concentrated under reduced pressure to give crude **15b** which was flash chromatographed (silica gel, EtOAc:hexanes, 1:19 → 1:9 v/v) to give pure **15b** (1.91 g, 71% two steps, 5:1 *E*/*Z*) as a light yellow oil.

Physical State: light yellow oil

$\mathbf{R_f}$ = 0.21 (EtOAc:hexanes, 1:9 v/v; vanillin)

$\mathbf{[\alpha]_D}$= −4.7 ° ($CHCl_3$, *c* 2.10)

^{1}**H-NMR** [$CDCl_3$, 600 MHz] δ: 5.90 (dd, *J* = 15.6, 5.9 Hz, 1H), 5.80 (ddd, *J* = 15.6, 6.2, 1.1 Hz, 1H), 5.38 (d, *J* = 6.2 Hz, 1H), 4.41 (t, *J* = 6.2 Hz, 1H), 3.77 – 3.70 (m, 3H), 1.41 (s, 3H), 1.40 (s, 3H), 1.22 (s, 9H), 1.21 (s, 3H), 0.90 (s, 9H), 0.07 (s, 3H), 0.06 (s, 3H).

^{13}**C-NMR** [$CDCl_3$, 150 MHz] δ: 132.7, 131.4, 109.4, 99.9, 81.4, 77.7, 62.6, 27.2, 27.1, 26.1, 24.1, 24.0, 22.1, -5.2, -5.3.

HREI MS (*m*/*z*): $(M)^+$ calcd. for $C_{21}H_{40}O_5Si$, 400.2645; found 400.2640.

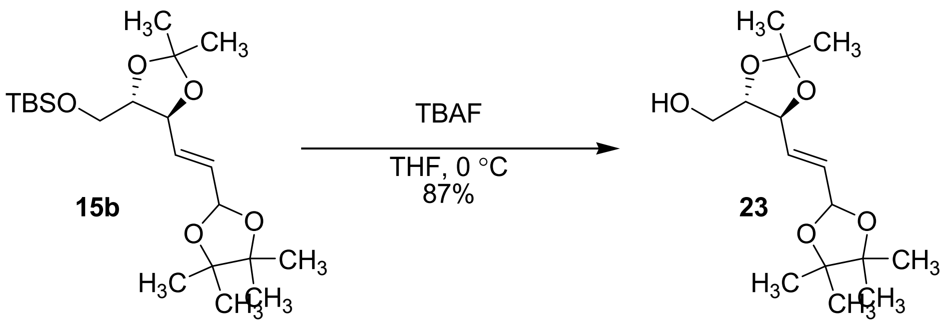

Synthesis of **23**: To **15b** (1.56 g, 3.89 mmol) in THF (15 mL) was added TBAF (1*M* in THF, 3.89 mL) at 0 °C. The mixture was stirred for 1 h at this temperature, concentrated *in vacuo* to remove the solvent and the residue was purified by column chromatography (silica gel, hexanes/ethyl acetate, 4:1 → 7:3 v/v) to afford **23** (0.972 g, 87%) as a colourless oil.

Spectroscopic data for (*E*)-**23**:

Physical State: colourless oil

$\mathbf{R}_f$ = 0.08 (EtOAc:hexanes, 3:7 v/v; vanillin)

$[\alpha]_D$ −5.7 ° ($CHCl_3$, *c* 1.02)

^{1}H-NMR [$CDCl_3$, 600 MHz] δ: 5.86 (dd, *J* = 15.5, 6.3 Hz, 1H), 5.81 (dd, *J* = 15.6, 5.5 Hz, 1H), 5.37 (d, *J* = 5.7 Hz, 1H), 4.38 (dd, *J* = 8.4, 6.2 Hz, 1H), 3.83 (dd, *J* = 12.1, 3.0 Hz, 1H), 3.81 – 3.77 (m, 1H), 3.60 (dd, *J* = 12.1, 3.8 Hz, 1H), 1.42 (s, 6H), 1.21 (s, 9H), 1.20 (s, 3H).

^{13}C-NMR [$CDCl_3$, 150 MHz] δ: 132.9, 131.8, 109.6, 99.6, 82.5, 81.2, 76.7, 60.9, 27.1, 27.1, 24.0, 24.0, 22.1.

HREI MS (*m/z*): $(M)^+$ calcd. for $C_{15}H_{26}O_5$, 286.1780; found 286.1770.

Spectroscopic data for (*Z*)-**23**:

Physical State: colourless oil

$\mathbf{R}_f$ = 0.18 (EtOAc:hexanes, 3:7 v/v; vanillin)

[α]D= −8.1 ° ($CHCl_3$, *c* 1.02)

^{1}H-NMR [$CDCl_3$, 600 MHz] δ: 5.77 – 5.73 (m, 2H), 5.73 – 5.66 (m, 1H), 4.70 (t, *J* = 8.6 Hz, 1H), 3.84 – 3.72 (m, 3H), 2.89 (dd, *J* = 8.5, 5.7 Hz, 1H), 1.43 (s, 6H), 1.23 (s, 6H), 1.22 (s, 3H), 1.19 (s, 3H).

^{13}C-NMR [$CDCl_3$, 150 MHz] δ: 133.8, 132.3, 109.4, 95.5, 83.0, 82.9, 80.9, 74.4, 61.2, 27.1, 27.1, 24.1, 23.8, 22.1, 22.0.

HREI MS (*m*/*z*): $(M)^+$ calcd. for $C_{15}H_{26}O_5$, 286.1780; found 286.1770.

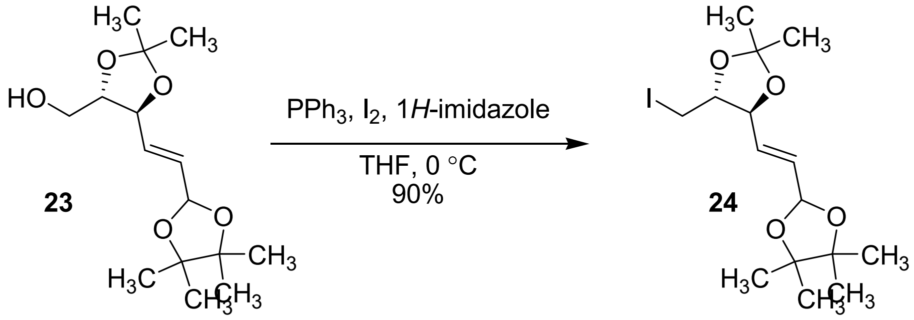

Synthesis of **24**: To a stirred solution of **23** (593 mg, 2.07 mmol) in dry THF (10 mL) were added successively, at 0 °C, 1*H*-imidazole (284 mg, 4.14 mmol), Ph_3P (652 mg, 2.48 mmol), and I_2 (628 mg, 2.48 mmol, in THF (2 mL)). The resulting mixture was stirred for 2 h at r.t. and then quenched by a 10% $Na_2S_2O_3$ solution (10 mL) and extracted with Et_2O (3 x 10 mL). The combined organic layers were then washed with brine and dried over anhydrous $MgSO_4$ prior to concentration under reduced pressure. The crude mixture was column chromatographed (silica gel, Et_2O/hexanes, 1:9 v/v) to afford iodide **24** (738 mg, 90%) as a colourless oil.

Physical State: colourless oil

R*f* = 0.32 (EtOAc:hexanes, 1:9 v/v; vanillin)

[α]D= −23.9° ($CHCl_3$, *c* 3.73)

^{1}H-NMR [$CDCl_3$, 600 MHz] δ: 5.83 (dd, J = 15.5, 6.2 Hz, 1H), 5.78 (dd, J = 15.6, 5.6 Hz, 1H), 5.32 (d, J = 5.7 Hz, 1H), 4.15 (dd, J = 7.5, 6.3 Hz, 1H), 3.57 (dt, J = 7.7, 4.9 Hz, 1H), 3.27 (dd, J = 10.8, 4.7 Hz, 1H), 3.16 (dd, J = 10.8, 5.2 Hz, 1H), 1.40 (s, 3H), 1.36 (s, 3H), 1.15 (s, 12H).

^{13}C-NMR [$CDCl_3$, 150 MHz] δ: 133.1, 131.5, 109.9, 99.5, 82.6, 81.3, 79.5, 27.4, 27.4, 24.1, 22.1, 5.0.

HREI MS (*m*/*z*): $(M–CH_3)^+$ calcd. for $C_{14}H_{22}IO_4$, 381.0563; found 381.0562.

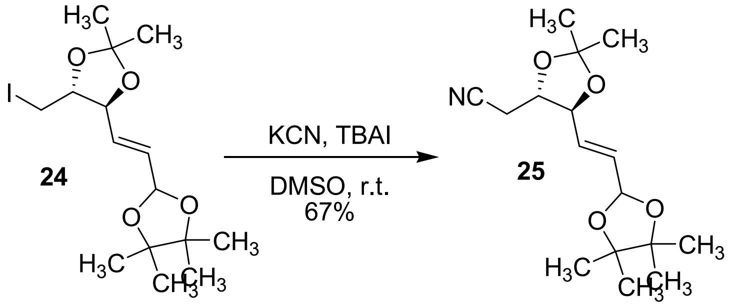

Synthesis of **25**: To iodide **24** (280 mg, 0.71 mmol) were added DMSO (0.5 mL), tetrabutylammonium iodide (26 mg, 0.07 mmol) and powdered KCN (92 mg, 1.41 mmol). The slightly yellow mixture was allowed to stir for 3 d at room temperature after which water (10 mL) was added to quench the reaction. Extraction with Et_2O (3 x 10 mL) followed by drying of the combined organic extracts with anhydrous $MgSO_4$ and concentration under reduced pressure gave the crude nitrile as a brown oil. Purification via column chromatography (silica gel, EtOAc/hexanes, 1:4 v/v) afforded nitrile **25** (136 mg, 67%) as a colourless oil.

Physical State: colourless oil

R_f = 0.24 (EtOAc:hexanes, 3:7 v/v; vanillin)

$[\alpha]_D$= −25.6 ° ($CHCl_3$, c 1.30)

^{1}H-NMR [$CDCl_3$, 600 MHz] δ: 5.92 (dd, J = 15.6, 5.7 Hz, 1H), 5.86 (dd, J = 15.6, 6.5 Hz, 1H), 5.41 (d, J = 5.6 Hz, 1H), 4.32 (dd, J = 8.2, 6.6 Hz, 1H), 3.87 (dt, J = 8.4, 4.7 Hz, 1H), 2.78 (dd, J = 17.1, 4.6 Hz, 1H), 2.60 (dd, J = 17.1, 4.9 Hz, 1H), 1.49 (s, 3H), 1.46 (s, 3H), 1.25 (s, 12H).

^{13}C-NMR [$CDCl_3$, 150 MHz] δ: 134.3, 129.7, 116.2, 110.3, 99.1, 82.5, 79.9, 75.4, 27.0, 26.9, 23.9, 21.9, 20.4.

HREI MS (*m*/*z*): $(M–CH_3)^+$ calcd. for $C_{15}H_{22}NO_4$, 280.1549; found 280.1555.

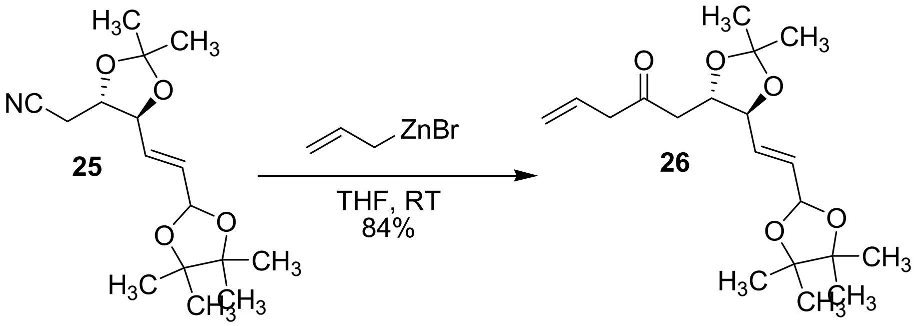

Synthesis of **26**: Allylzinc bromide was prepared using Knochel's procedure[8], which was slightly modified. In a flame-dried round-bottom flask fitted with magnetic bar and dropping funnel was placed zinc powder (0.78 g, 12 mmol), and the flask was flushed with dry nitrogen. Zinc powder was heated to 60–70 °C. 1,2-Dibromoethane (100 µL, 1.2 mmol) and THF (2 mL) was added, and the temperature was maintained for 10 min. The reaction mixture was then cooled to room temperature. Trimethylsilyl chloride (100 µL, 0.8 mmol) and THF (1 mL) was added. The mixture was stirred at room temperature for 15 min. After this step, allyl bromide (1.2 g, 10 mmol) in THF (5 mL) was added dropwise over 30 min, and the mixture was stirred for 2 min. The resulting opaque white solution was used immediately.

To a stirred solution of nitrile **25** (1.21 g, 4.10 mmol) in THF (3 mL) at room temperature was added allylzinc bromide (6.0 mL, 1.4 *M* in THF) dropwise. The reaction was observed to be complete after 1 h, as monitored by TLC, at which time a 10% aqueous solution of citric acid (30

mL) was added. The aqueous phase was extracted with Et_2O (4 x 10 mL) and the combined organic extracts dried over anhydrous magnesium sulphate prior to concentration under reduced pressure. The crude oil was then flash chromatographed (silica gel, Et_2O/hexanes, 1:4 v/v) to afford the allyl ketone **26** (1.16 g, 84%) as a colourless oil.

Physical State: colourless oil

$\mathbf{R}_f$ = 0.26 (EtOAc:hexanes, 1:4 v/v; vanillin)

$[\alpha]_D$= −17.3 ° ($CHCl_3$, *c* 2.12)

^{1}H-NMR [$CDCl_3$, 600 MHz] δ: 5.90 (ddt, *J* = 17.2, 10.2, 6.9 Hz, 1H), 5.83 (dd, *J* = 15.5, 5.6 Hz, 1H), 5.79 (dd, *J* = 15.5, 5.3 Hz, 1H), 5.35 (d, *J* = 5.3 Hz, 1H), 5.18 (ddd, *J* = 10.2, 2.8, 1.3 Hz, 1H), 5.13 (ddd, *J* = 17.2, 3.0, 1.5 Hz, 1H), 4.12 – 4.06 (m, 2H), 3.22 (dt, *J* = 6.9, 1.2 Hz, 2H), 2.68 (dd, *J* = 16.0, 7.7 Hz, 1H), 2.60 (dd, *J* = 16.0, 3.3 Hz, 1H), 1.39 (s, 3H), 1.37 (s, 3H), 1.20 – 1.19 (m, 12H).

^{13}C-NMR [$CDCl_3$, 150 MHz] δ: 205.7, 133.4, 131.1, 130.3, 119.3, 109.5, 99.5, 82.5, 82.5, 80.7, 76.3, 48.5, 44.3, 27.3, 27.1, 24.0, 24.0, 22.1.

HREI MS (*m*/*z*): $(M–CH_3)^+$ calcd. for $C_{18}H_{27}O_5$, 323.1858; found 323.1843.

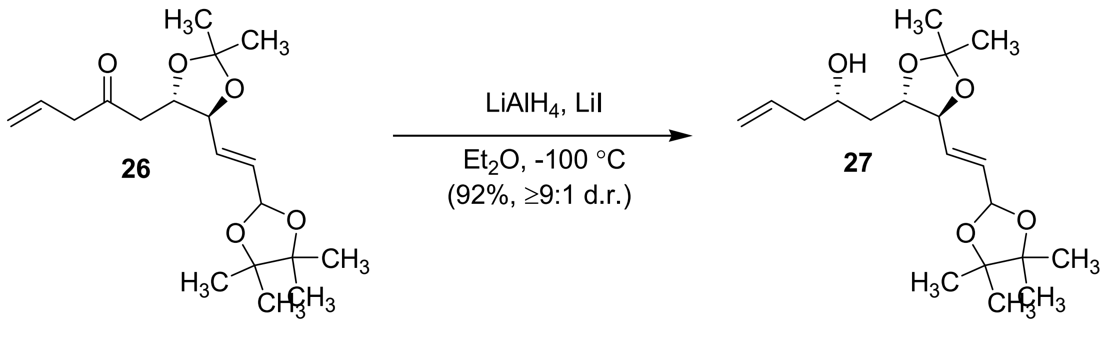

Synthesis of **27**: To a stirred solution of **26** (860 mg, 2.54 mmol) in ether (38 mL), LiI (2.20 g, 16.5 mmol) was added, and the resulting mixture was stirred at -40 °C for 10 minutes. The

reaction mixture was then cooled to -100 °C and $LiAlH_4$ (0.625 g, 16.5 mmol) was added to the reaction mixture in one portion. The reaction mixture was stirred at this temperature for 2 h and quenched with saturated aqueous sodium sulphate (1.5 mL). The crude suspension was then filtered through celite, water (50 mL) added and extracted with ether (3 x 20 ml). The combined organic layers were dried over anhydrous $MgSO_4$, concentrated under reduced pressure and the residue purified by column chromatography (silica gel, hexanes/EtOAc, 4:1 v/v) to afford alcohol **27** (798 mg, 92%, ≥9:1 d.r.) as an inseparable colourless oil.

Physical State: colourless oil

R_f = 0.17 (EtOAc:hexanes, 1:4 v/v; vanillin)

$[\alpha]_D$= −5.3 ° ($CHCl_3$, *c* 1.19)

^{1}H-NMR [$CDCl_3$, 600 MHz] δ: 5.89 – 5.77 (m, 3H), 5.38 (dd, *J* = 3.5, 1.8 Hz, 1H), 5.15 – 5.07 (m, 2H), 4.08 (ddd, *J* = 8.4, 3.7, 2.0 Hz, 1H), 3.86 (dddd, *J* = 9.0, 6.2, 5.9, 2.5 Hz, 1H), 3.81 (ddd, *J* = 9.8, 8.5, 2.8 Hz, 1H), 3.05 (bs, 1H), 2.32 – 2.18 (m, 2H), 1.77 (dt, *J* = 14.2, 2.7 Hz, 1H), 1.59 (dt, *J* = 14.2, 9.7 Hz, 1H), 1.41 (s, 6H), 1.22 (s, 12H).

^{13}C-NMR [$CDCl_3$, 150 MHz] δ: 134.7, 133.2, 131.2, 117.8, 109.7, 99.6, 82.6, 81.5, 80.6, 70.4, 41.9, 38.1, 27.3, 27.1, 24.1, 24.0, 22.1.

HREI MS (*m/z*): $(M)^+$ calcd. for $C_{19}H_{32}O_5$, 340.2250; found 340.2253.

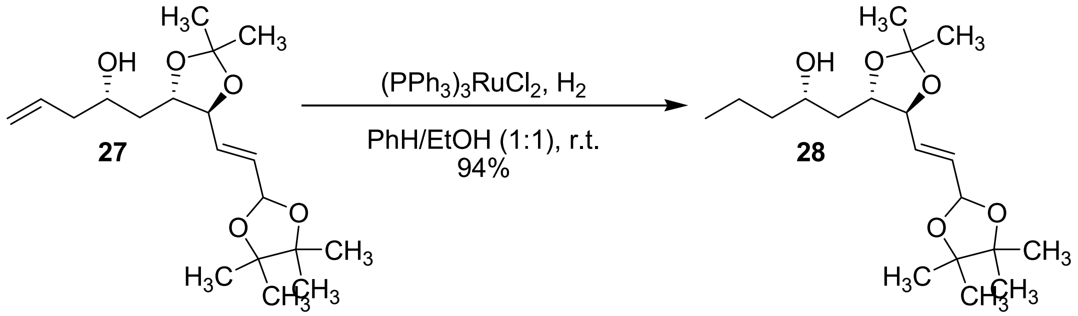

Synthesis of **28**: To **27** (1.21 g, 3.55 mmol) in benzene/ethanol (1:1 v/v, 20 mL) under an atmosphere of H_2 (1 atm) was added tris(triphenylphosphine)ruthenium dichloride (68 mg, 0.071 mmol). The violet reaction was stirred for 1 h at which point the hydrogen was allowed to dissipate and the reaction turned green, indicating deactivation of the catalytic species. The reaction was concentrated under reduced pressure and flash chromatographed (silica gel, EtOAc/hexanes, 1:4 v/v) to afford **28** (1.12 g, 94%) as a colourless oil.

Physical State: colourless oil

R_f = 0.19 (EtOAc:hexanes, 1:4 v/v; vanillin)

$[\alpha]_D$= −5.0 ° ($CHCl_3$, *c* 0.74)

^{1}H-NMR [$CDCl_3$, 600 MHz] δ: 5.85 – 5.77 (m, 2H), 5.38 (dd, *J* = 3.3, 2.0 Hz, 1H), 4.13 – 4.00 (m, 1H), 3.84 – 3.73 (m, 2H), 1.73 (dt, *J* = 14.2, 2.5 Hz, 1H), 1.56 (dt, *J* = 14.2, 9.8 Hz, 1H), 1.51 – 1.42 (m, 2H), 1.41 (s, 6H), 1.21 (s, 12H), 0.92 (t, *J* = 7.1 Hz, 3H).

^{13}C-NMR [$CDCl_3$, 150 MHz] δ: 133.2, 131.3, 109.6, 99.6, 82.5, 81.6, 80.9, 71.0, 39.7, 38.8, 27.3, 27.1, 24.04, 24.01, 22.1, 18.7, 14.2.

HRES MS (*m*/*z*): $(M+H)^+$ calcd. for $C_{19}H_{35}O_5$, 343.2484; found 343.2481.

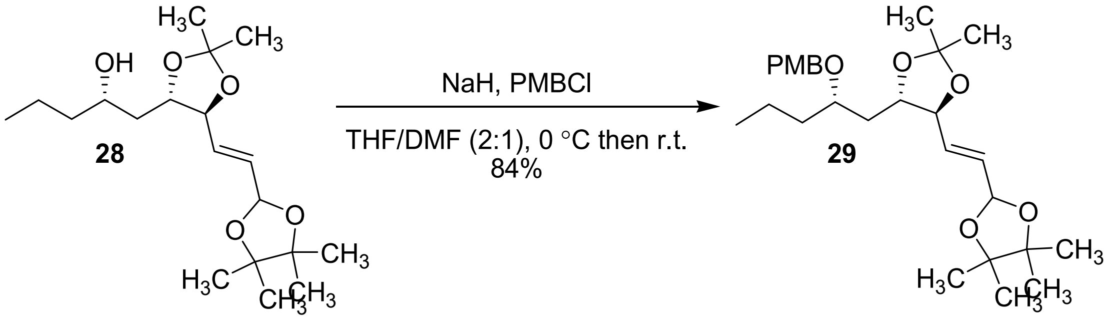

Synthesis of **29**: Sodium hydride (60% dispersion in mineral oil (5.6 mg, 0.14 mmol) was added to a stirred solution of alcohol **28** (40 mg, 0.12 mmol) and *p*-methoxybenzyl chloride (22 mg, 0.14 mmol) in THF (0.8 mL) and DMF (0.4 mL) at 0 °C, and stirring was continued for 16 h at room temperature. The reaction was quenched with saturated aqueous ammonium chloride (10 mL), and the mixture was diluted with diethyl ether (10 mL). The organic layer was washed with brine (5 mL), and then dried over Na_2SO_4. Concentration of the solvent under reduced pressure gave a residue, which was purified by column chromatography (silica gel, hexane/ethyl acetate, 9:1 v/v) to give **29** (56 mg, 84%) as a colourless oil.

Physical State: colourless oil

R_f = 0. (EtOAc:hexanes, 1:4 v/v; vanillin)

$[\alpha]_D$= −5.0 ° ($CHCl_3$, *c* 0.62)

^{1}H-NMR [CD_2Cl_2, 600 MHz] δ: 7.25 (d, *J* = 8.7 Hz, 2H), 6.85 (d, *J* = 8.7 Hz, 2H), 5.81 – 5.72 (m, 2H), 5.32 (m, 1H), 4.40 (d, *J* = 11.0 Hz, 1H), 4.38 (d, *J* = 11.0 Hz, 1H), 4.07 – 4.03 (m, 1H), 3.78 (s, 3H), 3.76 (td, *J* = 8.0, 4.0 Hz, 1H), 3.53 (p, *J* = 5.9 Hz, 1H), 1.84 (ddd, *J* = 13.8, 7.8, 5.8 Hz, 1H), 1.71 (ddd, *J* = 14.3, 6.1, 4.0 Hz, 1H), 1.54 – 1.49 (m, 2H), 1.43 – 1.33 (m, 8H), 1.19 (s, 3H), 1.19 (s, 3H), 1.18 (s, 3H), 1.17 (s, 3H), 0.91 (t, *J* = 7.3 Hz, 3H).

^{13}C-NMR [CD_2Cl_2, 150 MHz] δ: 159.2, 133.2, 131.6, 131.3, 129.4, 113.6, 108.6, 99.5, 82.2, 81.3, 77.9, 75.8, 70.2, 55.3, 36.1, 36.0, 27.1, 26.7, 23.8, 21.8, 18.4, 14.1.

HRES MS (*m*/*z*): $(M+Na)^+$ calcd. for $C_{27}H_{42}O_6Na$, 485.2887; found 485.2879.

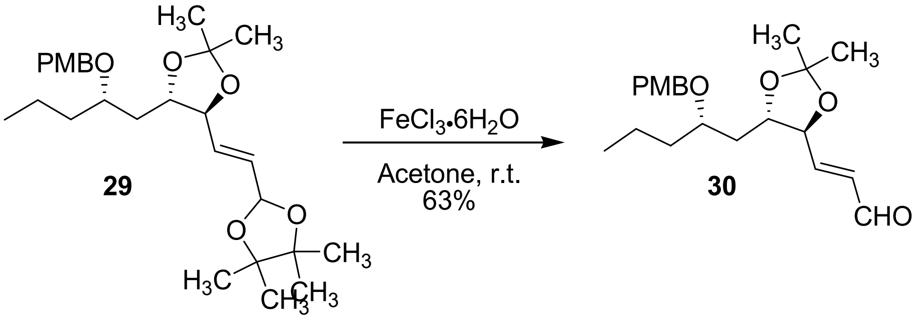

Synthesis of **30**: To acetal **29** (51 mg, 0.11 mmol) in acetone (5 mL) was added Iron(III) chloride hexahydrate (3.0 mg, 0.011 mmol). After stirring at room temperature for 10 h the reaction was quenched with a saturated $NaHCO_3$ solution. The reaction mixture was extracted with diethyl ether (3x 5 mL) and the combined organic extracts were dried over $MgSO_4$ and concentrated under reduced pressure to afford crude **30** (25 mg, 63%) which was used without further purification.

Physical State: light yellow oil

$\mathbf{R_f}$ = 0.24 (EtOAc:hexanes, 1:4 v/v; vanillin)

$\mathbf{[\alpha]_D}$= −9.6° ($CHCl_3$, *c* 2.60)

^{1}**H-NMR** [$CDCl_3$, 600 MHz] δ: 9.43 (d, *J* = 7.9 Hz, 1H), 7.16 (d, *J* = 8.7 Hz, 2H), 6.79 (d, *J* = 8.7 Hz, 2H), 6.63 (dd, *J* = 15.7, 5.4 Hz, 1H), 6.27 (ddd, *J* = 15.7, 7.9, 1.4 Hz, 1H), 4.40 (d, *J* = 11.2 Hz, 1H), 4.30 (d, *J* = 11.2 Hz, 1H), 4.27 (ddd, *J* = 8.3, 5.4, 1.3 Hz, 1H), 3.85 – 3.82 (m, 1H), 3.73 (s, 3H), 3.51 (p, *J* = 5.8 Hz, 1H), 1.90 (dt, *J* = 14.4, 6.4 Hz, 1H), 1.74 (dt, *J* = 14.4, 5.1 Hz, 1H), 1.52 – 1.44 (m, 2H), 1.38 (s, 3H), 1.35 – 1.31 (m, 5H), 0.85 (t, *J* = 7.3 Hz, 3H).

^{13}**C-NMR** [$CDCl_3$, 150 MHz] δ: 193.1, 159.2, 152.5, 132.7, 130.6, 129.3, 113.8, 109.6, 80.2, 77.7, 75.3, 70.3, 55.3, 36.1, 35.8, 27.3, 26.6, 18.4, 14.2.

HRES MS (*m/z*): $(M+Na)^+$ calcd. for $C_{21}H_{30}O_5Na$, 385.1986; found 385.1984.

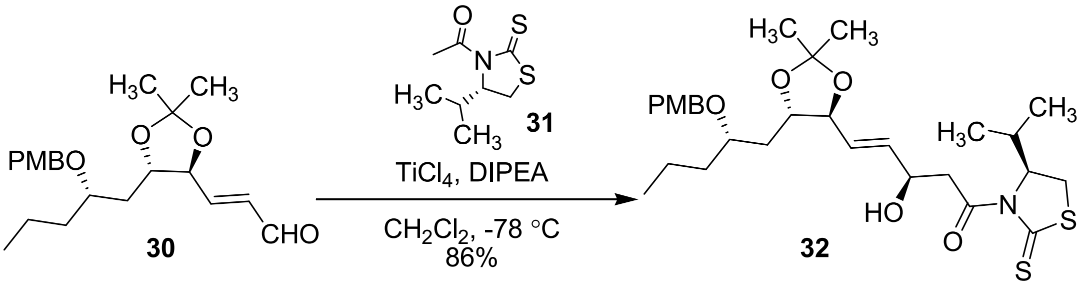

Synthesis of **32**: To a 0 °C solution of Nagao auxillary (+)-**31** (1.34 g, 5.91 mmol) in CH_2Cl_2 (45 mL) was added $TiCl_4$ (1.0 *M* in CH_2Cl_2, 6.60 mL, 6.60 mmol) and the resulting solution was stirred for 5 min. before cooling to –78 °C. DIPEA (*N,N*-Diisopropylethylamine) (1.15 mL, 6.60 mmol) was then added dropwise, and the resulting enolate solution was stirred for 2 h at –78 °C. Aldehyde **30** (1.26 g, 3.48 mmol) in CH_2Cl_2 (10 mL) was then added dropwise to the reaction mixture, and the resulting solution stirred at –78 °C for an additional 15 min. The reaction was quenched with 10 mL of saturated NH_4Cl solution, diluted with 20 mL CH_2Cl_2 and allowed to warm to room temperature. The layers were separated and the aqueous layer was extracted with CH_2Cl_2 (3 x 10 mL). The combined organic layers were then washed with brine (10 mL), dried over $MgSO_4$ and concentrated. Flash chromatography (silica gel, hexanes:EtOAc, 5:1 → 1:1) afforded allylic alcohol **32** (1.69 g, 86%, ≥50:1 d.r.) as a bright yellow oil.

Physical State: bright yellow oil

R_f = 0.10 (EtOAc:hexanes, 3:7 v/v; vanillin)

$[\alpha]_D$= +164.6° ($CHCl_3$, *c* 0.34)

^{1}H-NMR [$CDCl_3$, 600 MHz] δ: 7.27 (d, *J* = 7.5 Hz, 2H), 6.86 (d, *J* = 8.6 Hz, 2H), 5.87 (dd, *J* = 15.5, 5.2 Hz, 1H), 5.74 (ddd, *J* = 15.5, 7.4, 1.4 Hz, 1H), 5.16 – 5.11 (m, 1H), 4.69 – 4.65 (m, 1H), 4.45 – 4.39 (m, 2H), 4.05 (t, *J* = 7.9 Hz, 1H), 3.82 – 3.77 (m, 4H), 3.62 (dd, *J* = 17.6, 2.9 Hz, 1H), 3.58 – 3.50 (m, 2H), 3.28 (dd, *J* = 17.6, 8.9 Hz, 1H), 3.02 (d, *J* = 11.5 Hz, 1H), 2.36 (dq, *J* = 13.5, 6.8 Hz, 1H), 1.89 (ddd, *J* = 13.8, 7.4, 6.1 Hz, 1H), 1.71 (ddd, *J* = 14.2, 6.0, 4.2 Hz,

1H), 1.56 – 1.49 (m, 2H), 1.44 – 1.35 (m, 8H), 1.06 (d, *J* = 6.8 Hz, 3H), 0.98 (d, *J* = 6.9 Hz, 3H), 0.91 (t, *J* = 7.3 Hz, 3H).

^{13}C-NMR [$CDCl_3$, 150 MHz] δ: 203.1, 172.4, 159.2, 135.4, 131.1, 129.6, 129.5, 128.0, 113.9, 108.8, 82.0, 78.0, 75.9, 71.5, 70.4, 68.0, 55.5, 45.3, 36.2, 36.2, 31.0, 30.8, 27.5, 27.1, 19.2, 18.5, 18.0, 14.4.

HRES MS (*m/z*): $(M+H)^+$ calcd. for $C_{29}H_{44}NO_6S_2$, 566.2610; found 566.2600.

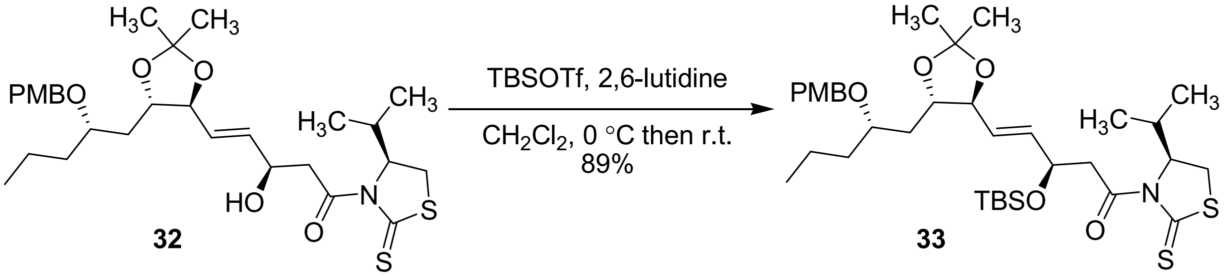

Synthesis of **33**: To a solution of alcohol **32** (1.36 g, 2.41 mmol) and 2,6-lutidine (0.56 mL, 4.81 mmol) in CH_2Cl_2 (10 mL) was added dropwise TBSOTf (0.52 mL, 2.89 mmol) at 0 °C. After stirring for 1 h at room temperature, saturated $NaHCO_3$ solution (20 mL) was added and the layers were separated. The aqueous layer was extracted with CH_2Cl_2 (3 × 10 mL). The combined organic layers were dried with $MgSO_4$, filtered, and concentrated under reduced pressure. The residue was purified by flash chromatography (petroleum ether/EtOAc, 9:1) to afford silyl ether **33** (1.45 g, 89%) as a yellow oil.

Physical State: bright yellow oil

R_f = 0.38 (EtOAc:hexanes, 1:9 v/v)

$[\alpha]_D$= +128.8° ($CHCl_3$, *c* 0.27)

^{1}H-NMR [CD_2Cl_2, 600 MHz] δ: 7.25 (d, *J* = 8.7 Hz, 2H), 6.85 (d, *J* = 8.7 Hz, 2H), 5.86 (ddd, *J* = 15.4, 6.0, 0.8 Hz, 1H), 5.65 (ddd, *J* = 15.4, 7.3, 1.2 Hz, 1H), 5.06 – 5.00 (m, 1H), 4.79 – 4.72

(m, 1H), 4.42 (d, J = 11.1 Hz, 1H), 4.38 (d, J = 11.1 Hz, 1H), 4.01 (t, J = 7.8 Hz, 1H), 3.79 (s, 3H), 3.70 (td, J = 8.3, 3.5 Hz, 1H), 3.63 (dd, J = 16.6, 8.1 Hz, 1H), 3.54 – 3.47 (m, 2H), 3.13 (dd, J = 16.6, 4.1 Hz, 1H), 3.04 (dd, J = 11.5, 0.9 Hz, 1H), 2.37 (dq, J = 13.6, 6.8 Hz, 1H), 1.82 (ddd, J = 13.9, 8.3, 5.4 Hz, 1H), 1.68 (ddd, J = 14.2, 6.7, 3.5 Hz, 1H), 1.53 – 1.48 (m, 2H), 1.45 – 1.39 (m, 1H), 1.39 (s, 3H), 1.37 (s, 3H), 1.35 – 1.30 (m, 1H), 1.04 (d, J = 6.8 Hz, 3H), 0.96 (d, J = 6.9 Hz, 3H), 0.90 (t, J = 7.3 Hz, 3H), 0.86 (s, 9H), 0.05 (s, 3H), 0.04 (s, 3H).

^{13}C-NMR [CD_2Cl_2, 150 MHz] δ: 203.6, 171.2, 159.5, 137.0, 131.7, 129.6, 127.8, 113.9, 108.9, 82.2, 78.3, 76.3, 72.2, 70.5, 69.9, 55.6, 46.7, 36.4, 36.2, 31.3, 27.5, 27.1, 26.0, 19.3, 18.8, 18.3, 17.9, 14.4, -4.0, -4.8.

HRES MS (*m*/*z*): $(M+H)^+$ calcd. for $C_{35}H_{58}NO_6SiS_2$, 680.3475; found 680.3499.

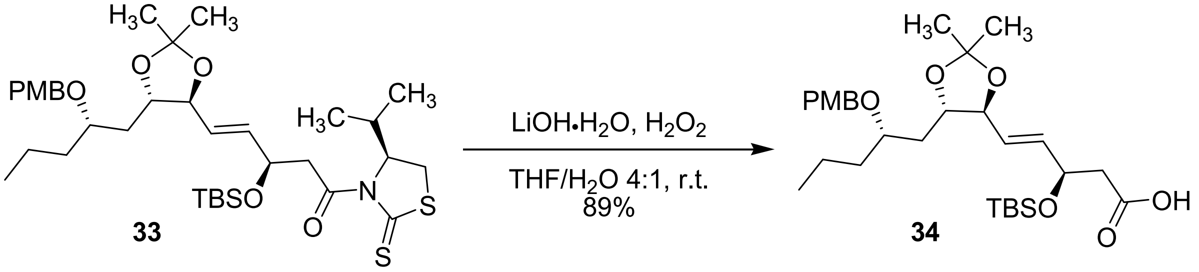

Synthesis of **34**: To a stirred solution of the above TBS ether **33** (1.56 g, 2.29 mmol) in THF:H_2O (4:1) (10 mL) at room temperature, LiOH·H_2O (295 mg, 7.02 mmol) and a 30% aqueous solution of H_2O_2 (0.70 mL) were added sequentially. The yellow color of the reaction mixture disappeared gradually. A TLC after 10 min indicated the completion of the reaction. The reaction mixture was then concentrated and the residue purified by flash column chromatography (silica gel, hexanes:EtOAc 4:1 v/v) to afford pure acid **34** (1.09 g, 89%) as a light yellow oil.

Physical State: light yellow oil

R_f = 0.41 (EtOAc:hexanes, 3:7 v/v)

$[\alpha]_D$= −7.5° ($CHCl_3$, *c* 0.38)

^{1}H-NMR [$CDCl_3$, 600 MHz] δ: 7.26 (d, *J* = 8.6 Hz, 2H), 6.87 (d, *J* = 8.6 Hz, 2H), 5.82 (dd, *J* = 15.5, 5.9 Hz, 1H), 5.68 (ddd, *J* = 15.4, 6.9, 1.1 Hz, 1H), 4.58 (dd, *J* = 11.6, 5.8 Hz, 1H), 4.04 (t, *J* = 7.6 Hz, 1H), 3.80 (s, 3H), 3.70 (td, *J* = 8.2, 3.4 Hz, 1H), 3.59 – 3.54 (m, 1H), 2.54 (dd, *J* = 15.2, 7.0 Hz, 1H), 2.50 (dd, *J* = 15.2, 5.2 Hz, 1H), 1.86 (ddd, *J* = 13.8, 8.1, 5.3 Hz, 1H), 1.69 (ddd, *J* = 14.3, 6.6, 3.4 Hz, 1H), 1.55 – 1.49 (m, 2H), 1.55 – 1.49 (m, 2H), 1.48 – 1.31 (m, 8H), 0.90 (t, *J* = 7.3 Hz, 3H), 0.88 (s, 9H), 0.07 (s, 3H), 0.04 (s, 3H).

^{13}C-NMR [$CDCl_3$, 150 MHz] δ: 173.8, 159.2, 135.3, 131.1, 129.5, 128.2, 113.9, 108.9, 81.5, 78.1, 75.9, 70.4, 69.6, 55.4, 42.9, 36.0, 35.9, 27.5, 27.0, 25.9, 18.5, 18.2, 14.4, -4.1, -5.0.

HRES MS (*m/z*): $(M+Na)^+$ calcd. for $C_{29}H_{48}NaO_7Si$, 559.3067; found 559.3060.

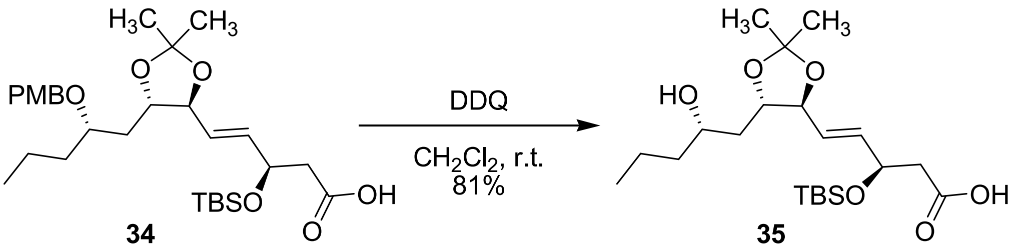

Synthesis of **35**: To **34** (102 mg, 0.190 mmol) in CH_2Cl_2 (8 mL) was added DDQ (47.4 mg, 0.209 mmol) in one portion at room temperature. The opaque mixture was allowed to stir for 1 h at which point TLC showed complete consumption of the starting material. The heterogeneous mixture was then concentrated under reduced pressure and the crude residue suspended in hexanes (10 mL) and filtered through sand to remove insoluble material. After exhaustive washing with hexanes (5 x 5 mL) the combined organic extracts where concentrated under reduced pressure the residue purified by column chromatography (silica gel, hexanes:EtOAc 9:1→ 4:1) to afford seco-acid **35** (64.1 mg, 81%) as a colourless oil.

Physical State: colourless oil

R$_f$ = 0.27 (EtOAc:hexanes, 3:7 v/v)

[α]$_D$= +2.4° ($CHCl_3$, *c* 0.92)

^{1}H-NMR [$CDCl_3$, 600 MHz] δ: 5.85 (dd, *J* = 15.5, 6.0 Hz, 1H), 5.67 (ddd, *J* = 15.5, 7.4, 1.1 Hz, 1H), 4.60 (q, *J* = 6.0 Hz, 1H), 4.02 (t, *J* = 7.9 Hz, 1H), 3.83 – 3.73 (m, 2H), 2.58 (dd, *J* = 14.9, 7.1 Hz, 1H), 2.50 (dd, *J* = 14.9, 5.8 Hz, 1H), 1.69 (dt, *J* = 14.2, 2.7 Hz, 1H), 1.58 (dt, *J* = 14.3, 9.4 Hz, 1H), 1.52 – 1.34 (m, 10H), 0.92 (t, *J* = 7.0 Hz, 3H), 0.88 (s, 9H), 0.07 (s, 3H), 0.05 (s, 3H).

^{13}C-NMR [$CDCl_3$, 150 MHz] δ: 175.2, 136.3, 127.6, 109.6, 82.0, 80.5, 70.9, 69.6, 43.4, 39.6, 38.5, 27.3, 27.1, 25.9, 18.8, 18.2, 14.2, -4.2, -5.0.

HRES MS (*m/z*): $(M+Na)^+$ calcd. for $C_{21}H_{40}NaO_6Si$, 439.2486; found 439.2484.

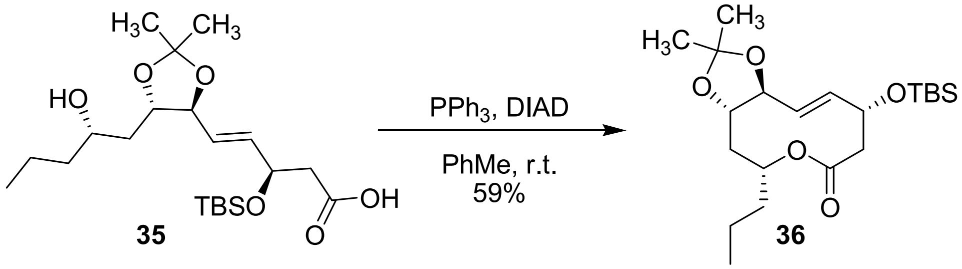

35
36

Synthesis of **36**: To a solution of triphenylphosphine (176 mg, 0.672 mmol) in PhMe (7 mL) at 0 °C was added diisopropyl azodicarboxylate (130 μL, 0.672 mmol) dropwise over five minutes. To this mixture was added **35** (28.1 mg, 0.067 mmol) in dry toluene (3 mL) over fifteen minutes. The resulting mixture was then allowed to stir at room temperature for 2 h. Removal of the solvent afforded a viscous yellow oil which was column chromatographed (silica gel, hexanes/EtOAc, 9:1) afforded lactone **36** (15.8 mg, 59%) as a colourless oil.

Physical State: colourless oil

R$_f$ = 0.28 (EtOAc:hexanes, 1:9 v/v)

[α]$_D$= −28.6° ($CHCl_3$, *c* 0.24)

^{1}H-NMR [$CDCl_3$, 600 MHz] δ: 5.84 (dd, *J* = 15.8, 9.1 Hz, 1H), 5.30 (dd, *J* = 15.8, 9.3 Hz, 1H), 5.02 (ddd, *J* = 10.2, 8.1, 5.6 Hz, 1H), 4.46 (td, *J* = 9.6, 6.1 Hz, 1H), 3.97 (t, *J* = 8.8 Hz, 1H), 3.60 (t, *J* = 8.8 Hz, 1H), 2.69 (dd, *J* = 10.7, 6.1 Hz, 1H), 2.39 (t, *J* = 10.4 Hz, 1H), 2.04 (d, *J* = 15.4 Hz, 1H), 1.89 (dt, *J* = 15.4, 10.0 Hz, 1H), 1.56 – 1.49 (m, 1H), 1.48 – 1.42 (m, 1H), 1.40 (s, 3H), 1.39 (s, 3H), 1.32 – 1.24 (m, 2H), 0.89 (t, *J* = 7.4 Hz, 3H), 0.87 (s, 9H), 0.06 (s, 3H), 0.05 (s, 3H).

^{13}C-NMR [$CDCl_3$, 150 MHz] δ: 170.1, 139.0, 124.4, 108.4, 83.6, 81.7, 72.4, 72.2, 46.8, 38.2, 37.0, 27.2, 27.0, 25.9, 18.6, 18.2, 14.0, -4.4, -4.8.

HRES MS (*m/z*): $(M+Na)^+$ calcd. for $C_{21}H_{38}O_5SiNa$, 421.2386; found 421.2395.

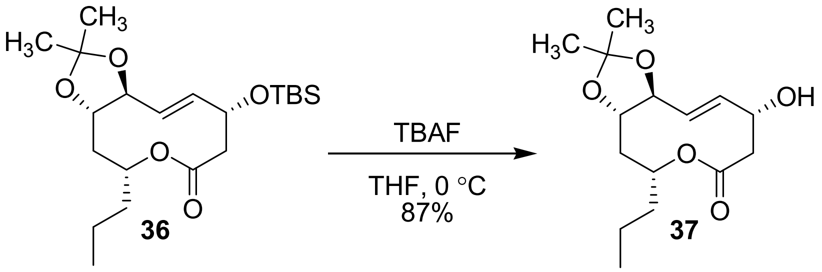

Synthesis of **37**: To lactone **36** (12.3 mg, 0.031 mmol) in THF (3 mL) was added TBAF (1 *M* in THF, 34 µL) at 0 °C and the resulting slightly yellow solution was stirred for 1 h. The reaction was quenched with acetic acid (20 µL) and concentrated under reduced pressure. The crude oil was then column chromatographed (silica gel, hexanes:EtOAc 6:4) furnished **37** (7.6 mg, 87%) as a colourless oil.

Physical State: colourless oil

R$_f$ = 0.18 (EtOAc:hexanes, 4:6 v/v)

[α]$_D$= −27.5° ($CHCl_3$, *c* 0.37)

^{1}H-NMR [$CDCl_3$, 600 MHz] δ: 5.86 (dd, J = 15.8, 9.2 Hz, 1H), 5.36 (dd, J = 15.8, 9.3 Hz, 1H), 5.02 (ddd, J = 10.2, 8.1, 5.7 Hz, 1H), 4.53 (td, J = 9.9, 6.0 Hz, 1H), 3.97 (t, J = 8.8 Hz, 1H), 3.60 (t, J = 8.9 Hz, 1H), 2.80 (dd, J = 10.5, 6.0 Hz, 1H), 2.39 (t, J = 10.5 Hz, 1H), 2.05 (d, J = 15.4 Hz, 1H), 1.91 (dt, J = 15.5, 10.0 Hz, 1H), 1.57 – 1.49 (m, 1H), 1.49 – 1.42 (m, 1H), 1.39 (s, 3H), 1.38 (s, 3H), 1.29 (dq, J = 14.8, 7.4 Hz, 2H), 0.89 (t, J = 7.4 Hz, 3H).

^{13}C-NMR [$CDCl_3$, 150 MHz] δ: 169.9, 137.8, 126.1, 108.6, 83.5, 81.7, 72.7, 71.5, 45.7, 38.2, 36.9, 27.2, 27.0, 18.6, 14.0.

HRES MS (*m/z*): $(M+Na)^+$ calcd. for $C_{15}H_{24}O_5Na$, 307.1521; found 307.1525.

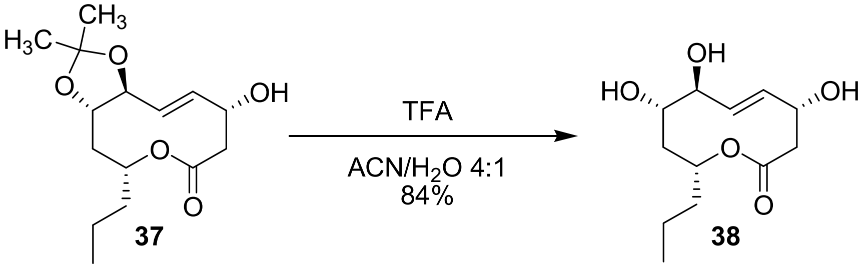

Synthesis of **38**: To a solution of **37** (6.2 mg, 0.02 mmol) in acetonitrile/water (4:1 v/v, 0.4 mL) was added TFA (25 µl) at room temperature. The reaction mixture was stirred at this temperature for 16 h, at which point $NaHCO_3$ (27 mg) was added and the reaction allowed to stir for 15 min. The solvent was removed under a stream of nitrogen, the residue taken up in EtOAc (1 mL) and the suspension filtered through Na_2SO_4 which was subsequently rinsed with EtOAc (2x 1 mL). The combined organic extracts were concentrated and the crude residue purified by column chromatography (silica gel, EtOAc → DCM/MeOH 19:1 v/v) to afford **38** (4.5 mg, 84%) as an amporphous white solid.

Physical State: white solid

R_f = 0.31 (DCM:MeOH, 9:1 v/v; vanillin)

$[\alpha]_D$ = −69.9° (MeOH, c 0.32) (lit.[9] $[\alpha]_D$ −10.4° (MeOH, c 0.24))

Corrected to Acetone

^{1}H-NMR [Acetone-d_6, 600 MHz] δ: 5.71 (dd, *J* = 15.8, 8.6 Hz, 1H), 5.16 (dd, *J* = 15.8, 9.4 Hz, 1H), 4.82 – 4.74 (m, 1H), 4.47 (br s, 1H), 4.32 (dd, *J* = 15.6, 9.3 Hz, 1H), 4.24 (br s, 1H), 3.84 (br s, 1H), 3.67 (t, *J* = 9.1 Hz, 1H), 3.38 – 3.27 (m, 1H), 2.61 (dd, *J* = 10.2, 5.8 Hz, 1H), 2.31 (t, *J* = 10.4 Hz, 1H), 1.88 – 1.82 (m, 2H), 1.55 – 1.43 (m, 2H), 1.33 – 1.25 (m, 2H), 0.89 (t, *J* = 7.4 Hz, 3H).

^{13}C-NMR [Acetone-d_6, 150 MHz] δ: 170.7, 137.3, 129.0, 79.0, 76.9, 73.4, 72.5, 46.1, 42.0, 39.9, 19.0, 14.1.

Corrected to the Natural Product:

^{1}H-NMR [Acetone-d_6, 600 MHz] δ: 5.61 (dd, *J* = 15.8, 8.6 Hz, 1H), 5.06 (dd, *J* = 15.8, 9.4 Hz, 1H), 4.72 – 4.64 (m, 1H), 4.37 (br s, 1H), 4.22 (dd, *J* = 15.6, 9.3 Hz, 1H), 4.14 (br s, 1H), 3.74 (br s, 1H), 3.57 (t, *J* = 9.1 Hz, 1H), 3.28 – 3.17 (m, 1H), 2.51 (dd, *J* = 10.2, 5.8 Hz, 1H), 2.21 (t, *J* = 10.4 Hz, 1H), 1.72 – 1.69 (m, 2H), 1.45 – 1.33 (m, 2H), 1.23 – 1.15 (m, 2H), 0.79 (t, *J* = 7.4 Hz, 3H).

^{13}C-NMR [Acetone-d_6, 150 MHz] δ: 169.9, 136.4, 128.1, 78.1, 76.0, 72.5, 71.6, 45.2, 41.1, 39.0, 18.1, 13.2.

HRES MS (*m*/*z*): $(M+Na)^+$ calcd. for $C_{12}H_{20}O_5Na$, 267.1208; found 267.1209.

---

[1] Nagao, Y.; Dai, W.-M.; Ochiai, M.; Shiro, M. *J. Org. Chem.* **1989**, *54*, 5211.
[2] McNulty, J., McLeod, D.; Jenkins, H. A. *Eur. J. Org. Chem.* **2016**, 688.
[3] Fernandes, R. A. *Eur. J. Org. Chem.* **2007**, 5064.
[4] Mukaiyama, T.; Suzuki, K.; Yamada, T.; Tabusa, F. *Tetrahedron*, **1990**, *46*, 265-276.
[5] Jana, N.; Nanda, S. *Eur. J. Org. Chem.* **2012**, 4313.
[6] Garner, P.; Park, J. M. *Org. Synth.,* 70, **1992**, 18.
[7] Smith III, A. B.; Rano, T. A.; Chida, N.; Sulikowski, G. A.; Wood, J. L. *J. Am. Chem. Soc.* **1992**, *114*, 8008.
[8] Knochel, P.; Yeh, M. C. P.; Berk, S. C.; Talbert, J. *J. Org. Chem.* **1988**, *53*, 2390.
[9] Li, Y.-Y.; Wang, M.-Z.; Huang, Y.-J.; Shen, Y.-M. *Mycologyy,* **2010**, *1*, 254.

SUPPORTING INFORMATION

Part 2: NMR Spectra

Table of Contents

Spectra for compounds.. page SI~39-92

Compound **9** ^{1}H-NMR.. page SI-39
Compound **9** ^{13}C-NMR.. page SI-40
Compound **11** ^{1}H-NMR.. page SI-41
Compound **11** ^{13}C-NMR.. page SI-42
Compound **13** ^{1}H-NMR.. page SI-43
Compound **13** ^{13}C-NMR.. page SI-44
Compound **15a** ^{1}H-NMR.. page SI-45
Compound **15a** ^{13}C-NMR.. page SI-46
Compound **17** ^{1}H-NMR.. page SI-47
Compound **17** ^{13}C-NMR.. page SI-48
Compound **19** ^{1}H-NMR.. page SI-49
Compound **19** ^{13}C-NMR.. page SI-50
Compound **20** ^{1}H-NMR.. page SI-51
Compound **20** ^{13}C-NMR.. page SI-52
Compound **21** ^{1}H-NMR.. page SI-53
Compound **21** ^{13}C-NMR.. page SI-54
Compound **22** ^{1}H-NMR.. page SI-55
Compound **22** ^{13}C-NMR.. page SI-56
Compound **15b** ^{1}H-NMR.. page SI-57
Compound **15b** ^{13}C-NMR.. page SI-58
Compound **(*E*)-23** ^{1}H-NMR.. page SI-59
Compound **(*E*)-23** ^{13}C-NMR.. page SI-60
Compound **(*Z*)-23** ^{1}H-NMR.. page SI-61
Compound **(*Z*)-23** ^{13}C-NMR.. page SI-62
Compound **24** ^{1}H-NMR.. page SI-63
Compound **24** ^{13}C-NMR.. page SI-64
Compound **25** ^{1}H-NMR.. page SI-65
Compound **25** ^{13}C-NMR.. page SI-66
Compound **26** ^{1}H-NMR.. page SI-67
Compound **26** ^{13}C-NMR.. page SI-68
Compound **27** ^{1}H-NMR.. page SI-69
Compound **27** ^{13}C-NMR.. page SI-70
Compound **28** ^{1}H-NMR.. page SI-71

Compound **28** ^{13}C-NMR.. page SI-72
Compound **29** ^{1}H-NMR.. page SI-73
Compound **29** ^{13}C-NMR.. page SI-74
Compound **30** ^{1}H-NMR... page SI-75
Compound **30** ^{13}C-NMR.. page SI-76
Compound **32** ^{1}H-NMR... page SI-77
Compound **32** ^{13}C-NMR.. page SI-78
Compound **33** ^{1}H-NMR... page SI-79
Compound **33** ^{13}C-NMR.. page SI-80
Compound **34** ^{1}H-NMR... page SI-81
Compound **34** ^{13}C-NMR.. page SI-82
Compound **35** ^{1}H-NMR... page SI-83
Compound **35** ^{13}C-NMR.. page SI-84
Compound **36** ^{1}H-NMR... page SI-85
Compound **36** ^{13}C-NMR.. page SI-86
Compound **37** ^{1}H-NMR... page SI-87
Compound **37** ^{13}C-NMR.. page SI-88
Compound **38** ^{1}H-NMR.. page SI-89
Compound **38** ^{13}C-NMR.. page SI-90
Compound **38** COSY.. page SI-91
Compound **38** HSQC.. page SI-92

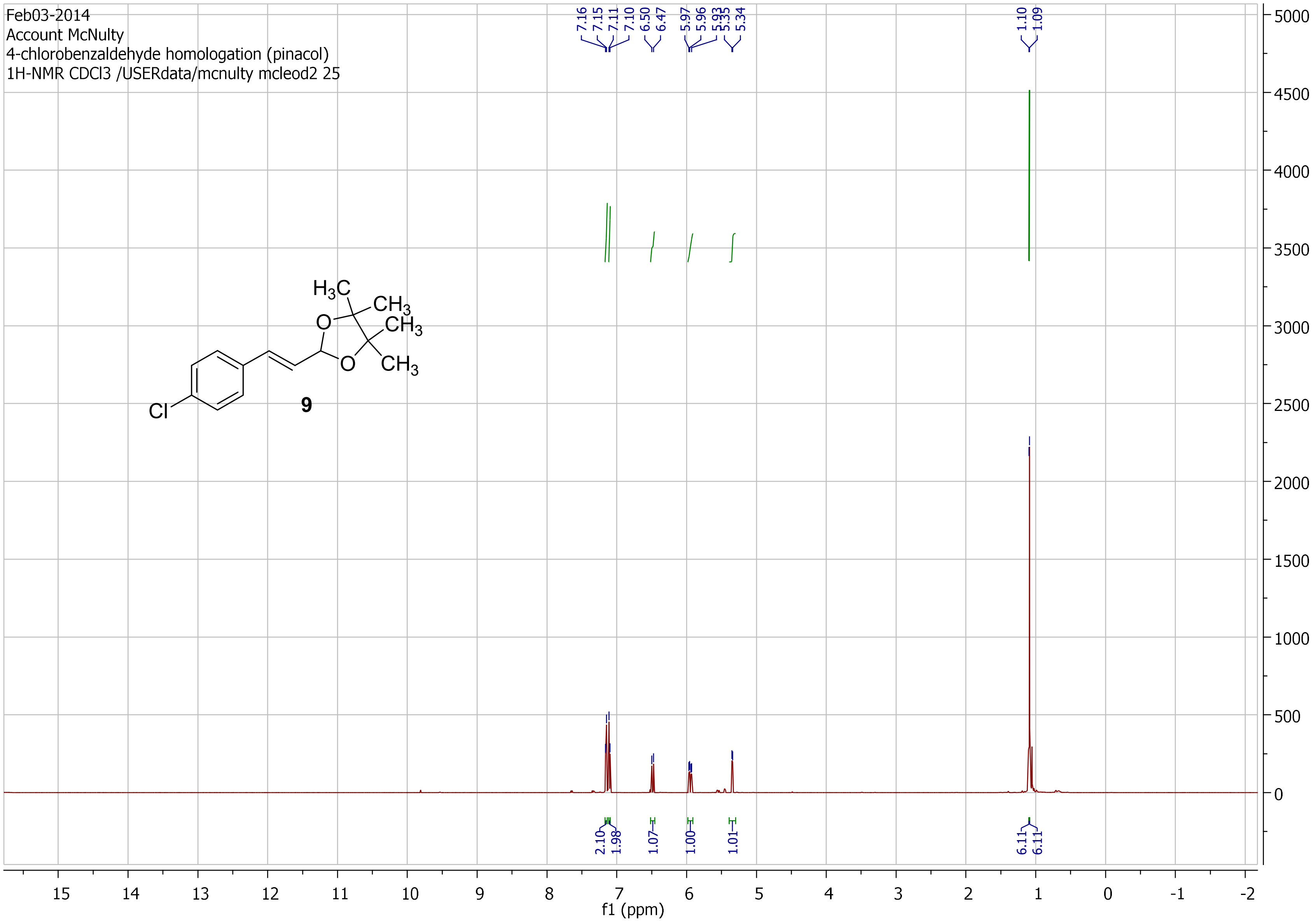
Feb03-2014
Account McNulty
4-chlorobenzaldehyde homologation (pinacol)
1H-NMR CDCl3 /USERdata/mcnulty mcleod2 25
7.16
7.15
7.11
7.10
6.50
6.47
5.97
5.96
5.93
5.35
5.34
1.10
1.09
9
2.10
1.98
1.07
1.00
1.01
6.11
6.11
f1 (ppm)

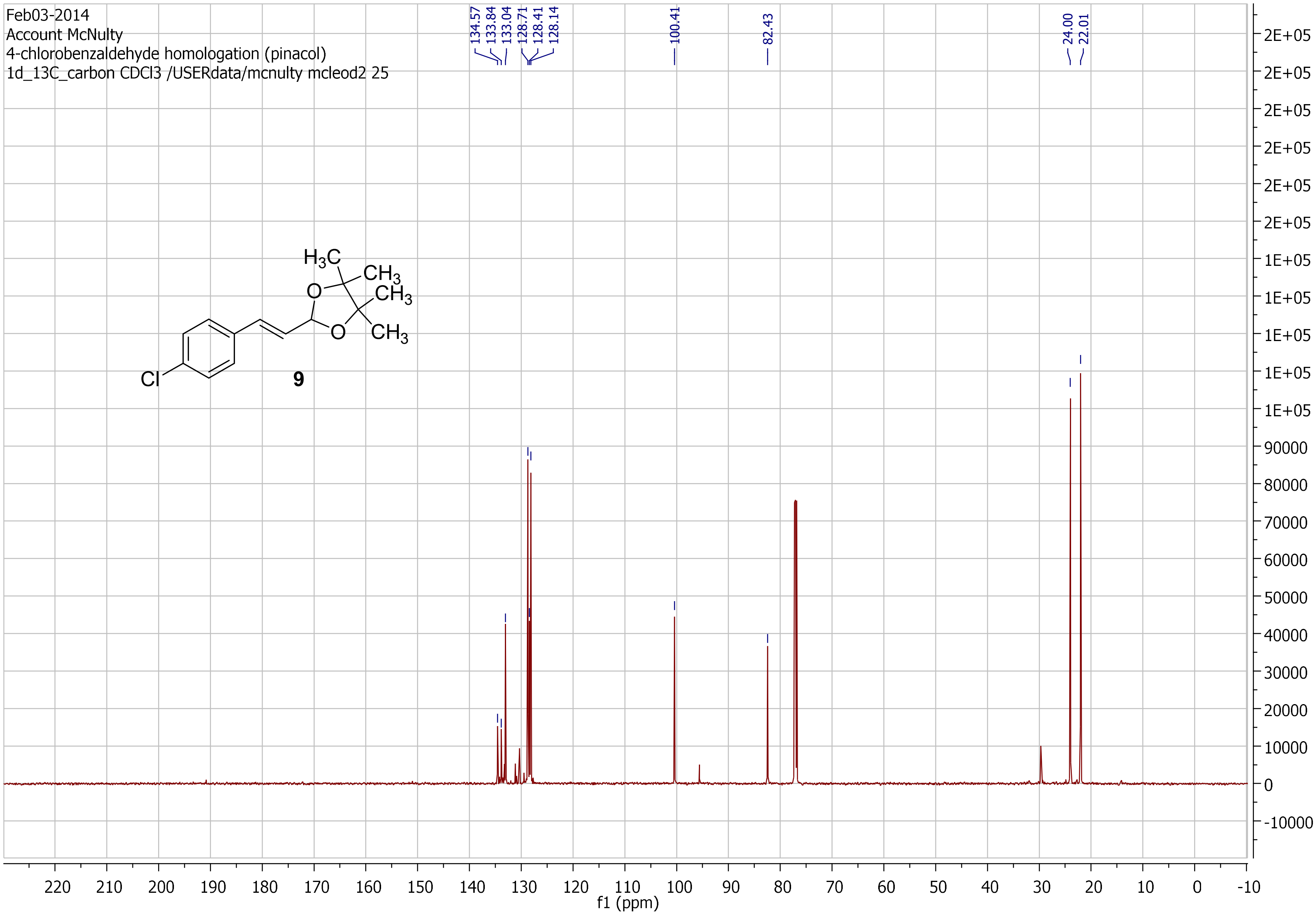
Feb03-2014
Account McNulty
4-chlorobenzaldehyde homologation (pinacol)
1d_13C_carbon CDCl3 /USERdata/mcnulty mcleod2 25
134.57
133.84
133.04
128.71
128.41
128.14
100.41
82.43
24.00
22.01
f1 (ppm)
9

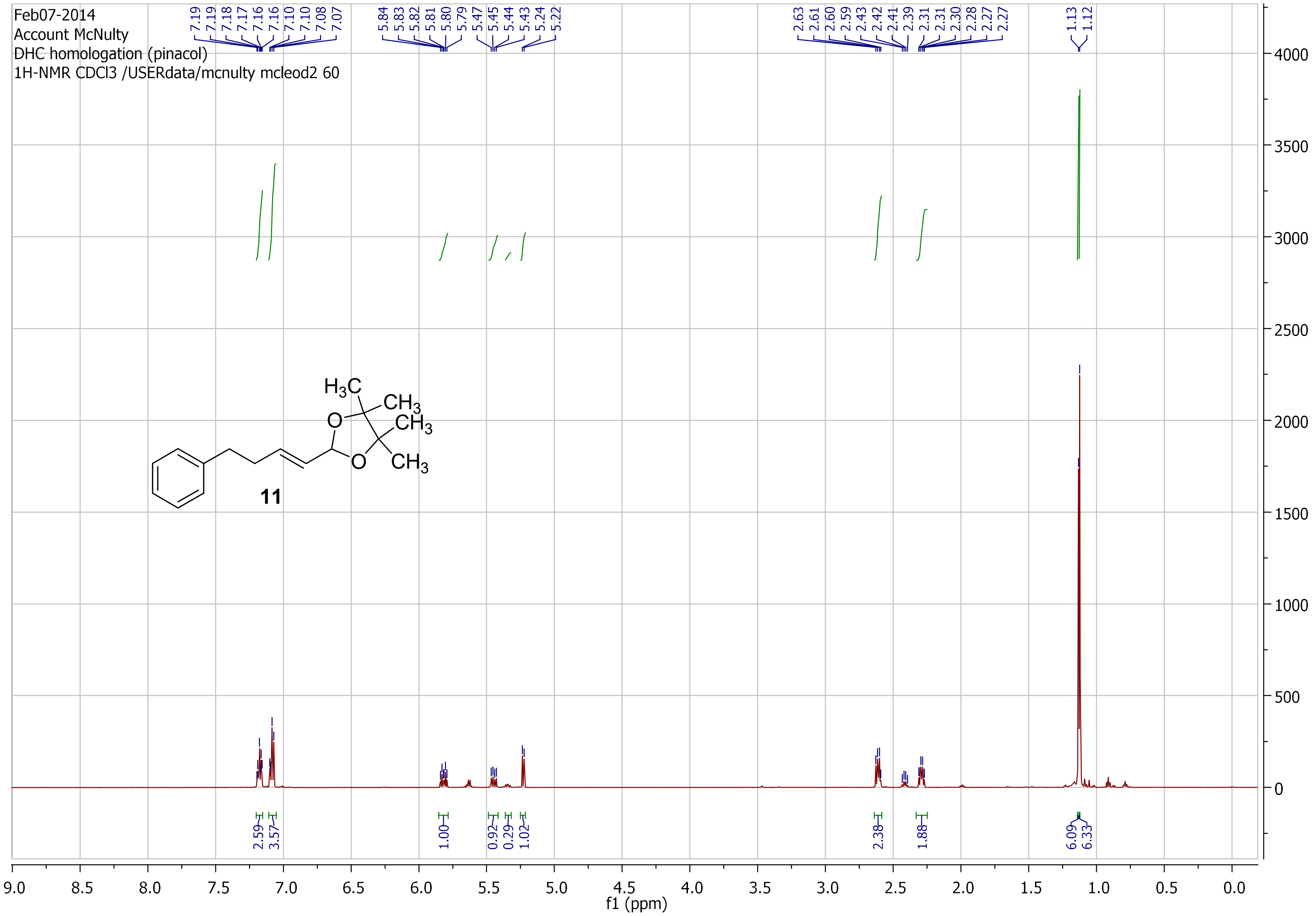

Feb07-2014
Account McNulty
DHC homologation (pinacol)
1H-NMR CDCl3 /USERdata/mcnulty mcleod2 60
7.19 7.19 7.18 7.17 7.16 7.16 7.10 7.10 7.08 7.07
5.84 5.83 5.82 5.81 5.80 5.79 5.47 5.45 5.44 5.43 5.24 5.22
2.63 2.61 2.60 2.59 2.43 2.42 2.41 2.39 2.31 2.31 2.30 2.28 2.27 2.27
1.13 1.12
11
2.59 3.57 1.00 0.92 0.29 1.02 2.38 1.88 6.09 6.33
f1 (ppm)

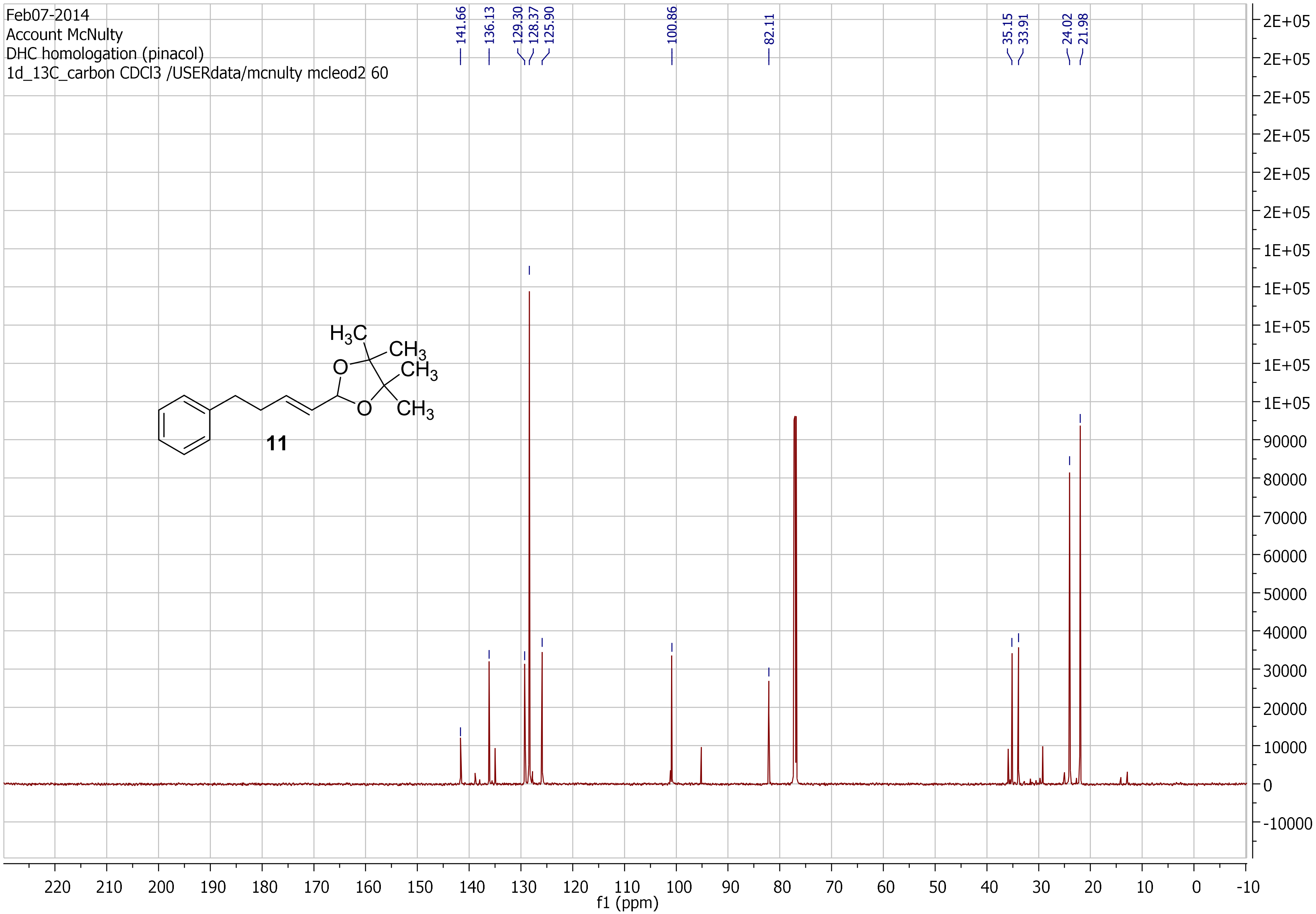
Feb07-2014
Account McNulty
DHC homologation (pinacol)
1d_13C_carbon CDCl3 /USERdata/mcnulty mcleod2 60
141.66
136.13
129.30
128.37
125.90
100.86
82.11
35.15
33.91
24.02
21.98
H3C
CH3
CH3
CH3
O
O
11
f1 (ppm)

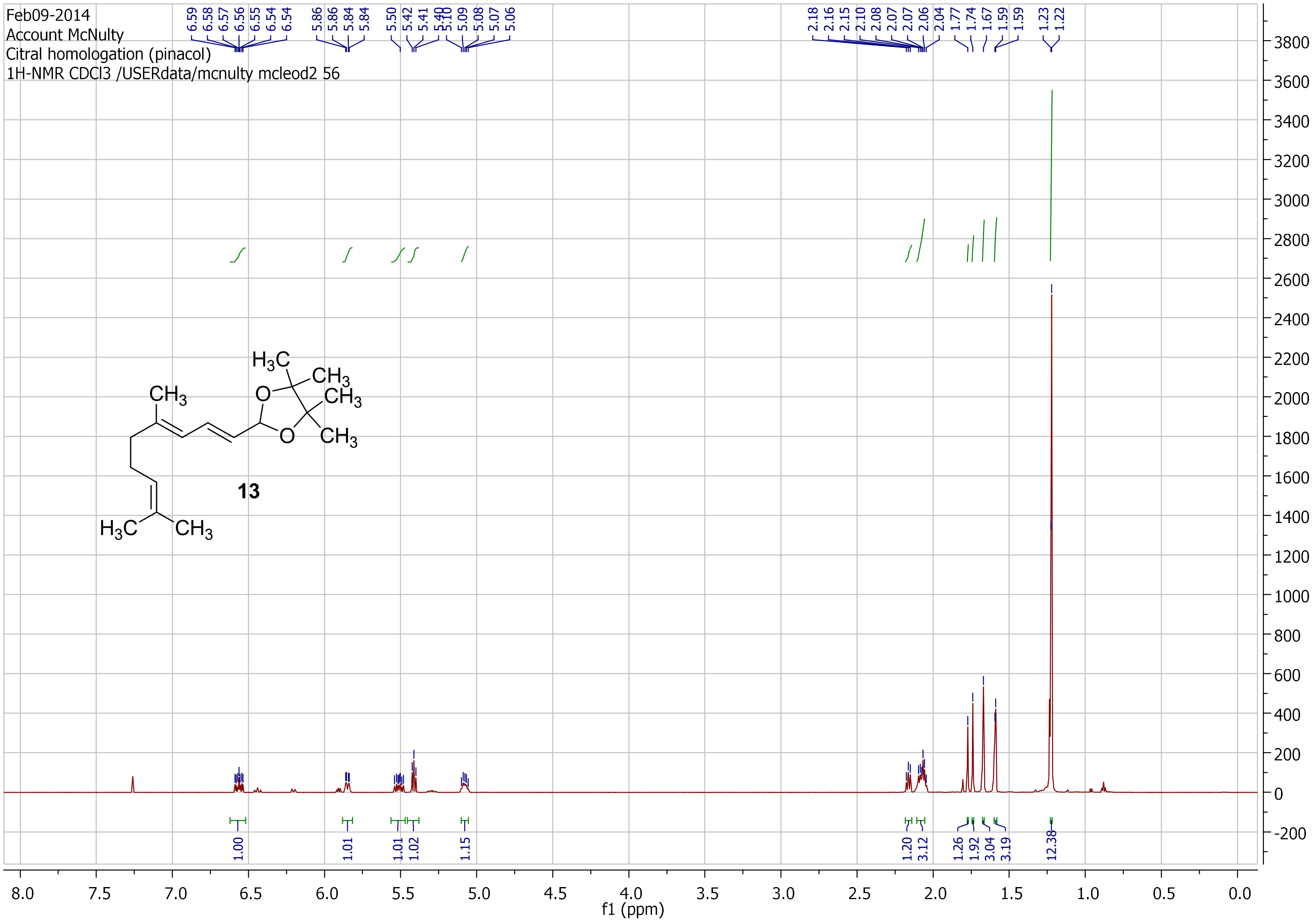
Feb09-2014
Account McNulty
Citral homologation (pinacol)
1H-NMR CDCl3 /USERdata/mcnulty mcleod2 56
13
f1 (ppm)

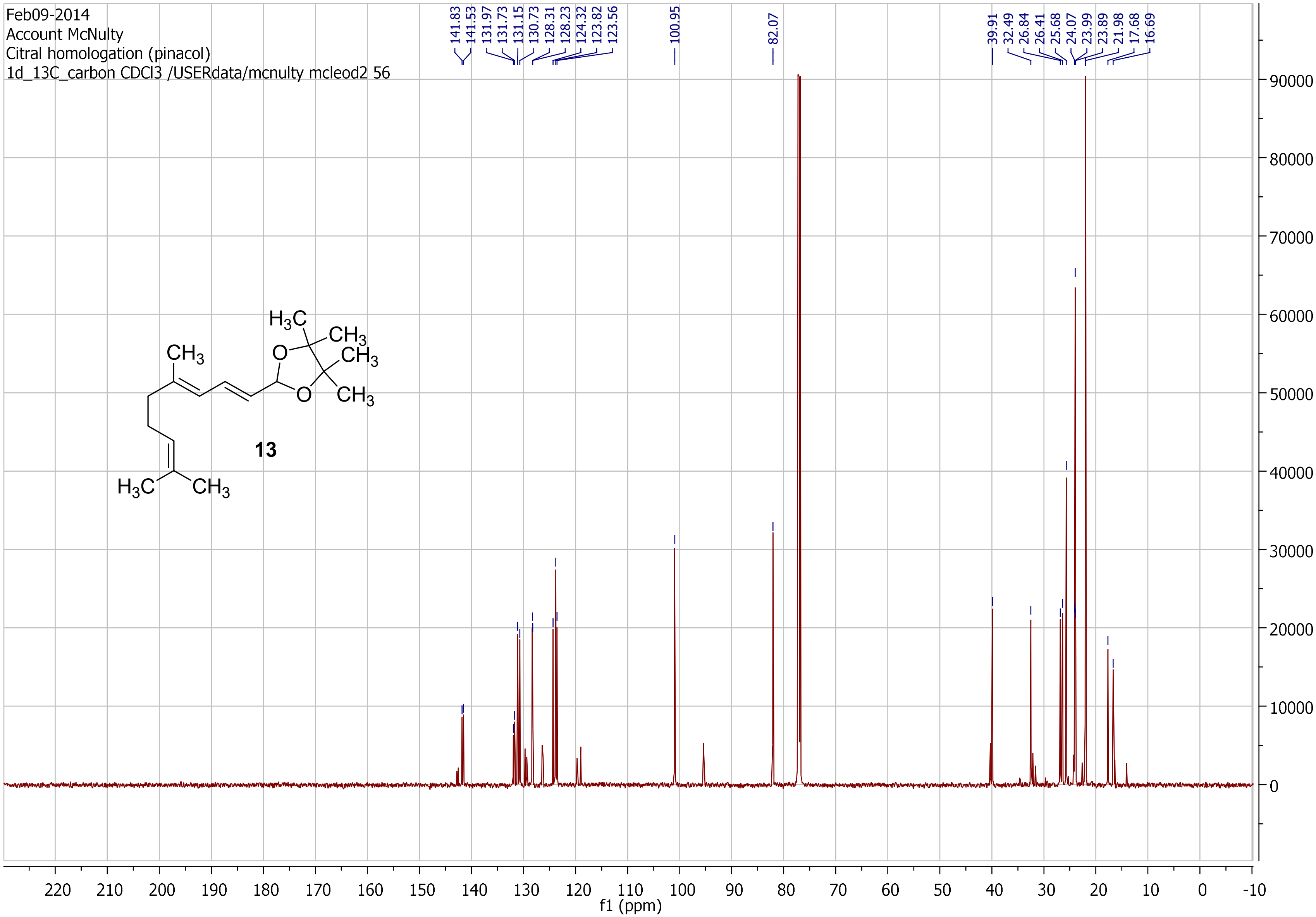

Feb09-2014
Account McNulty
Citral homologation (pinacol)
1d_13C_carbon CDCl3 /USERdata/mcnulty mcleod2 56
141.83
141.53
131.97
131.73
131.15
130.73
128.31
128.23
124.32
123.82
123.56
100.95
82.07
39.91
32.49
26.84
26.41
25.68
24.07
23.99
23.89
21.98
17.68
16.69
13
f1 (ppm)

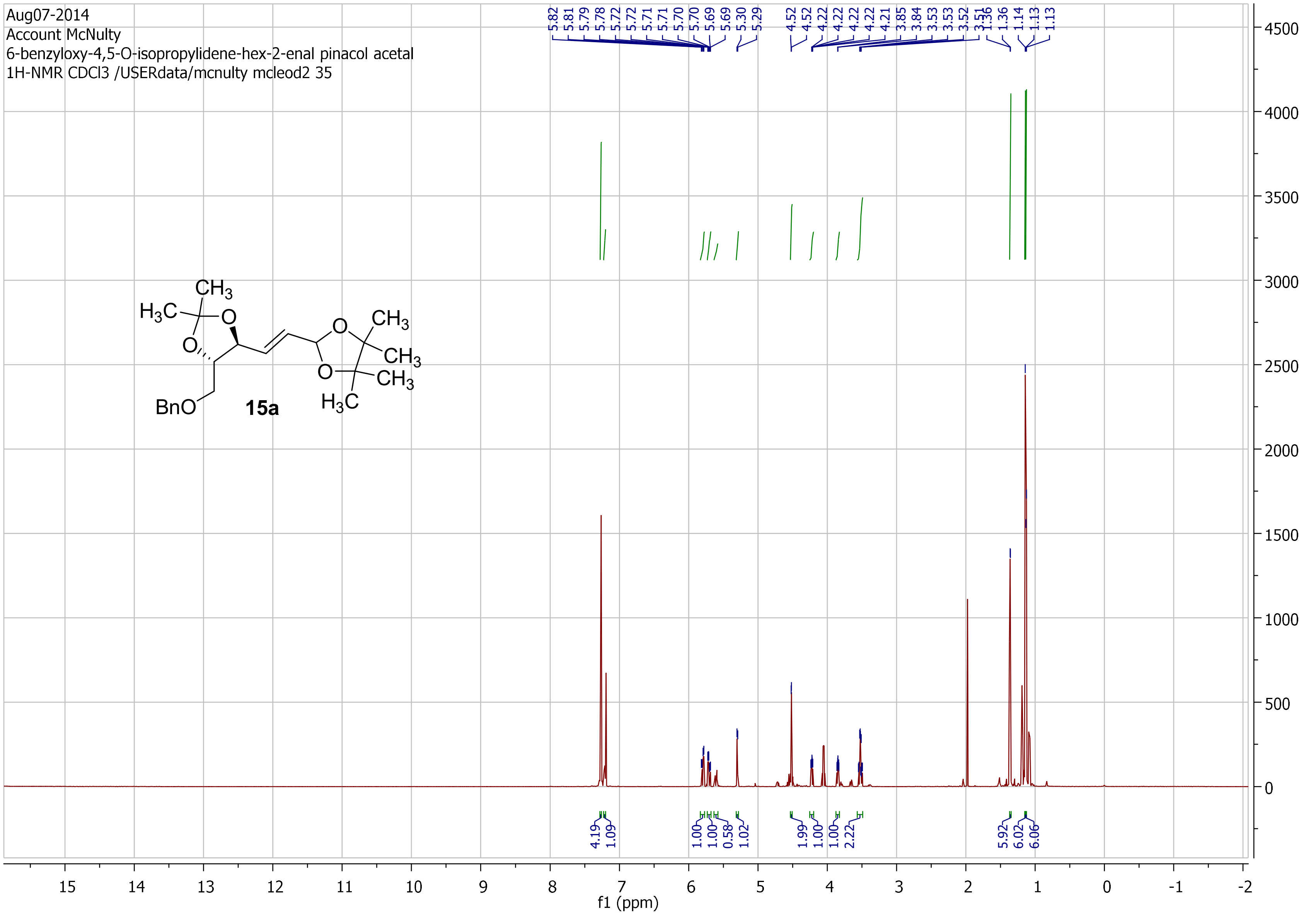
Aug07-2014
Account McNulty
6-benzyloxy-4,5-O-isopropylidene-hex-2-enal pinacol acetal
1H-NMR CDCl3 /USERdata/mcnulty mcleod2 35
5.82 5.81 5.79 5.78 5.72 5.72 5.71 5.71 5.70 5.70 5.69 5.69 5.30 5.29 4.52 4.52 4.22 4.22 4.22 4.22 4.21 3.85 3.84 3.53 3.53 3.52 3.51 1.36 1.14 1.13 1.13
BnO
15a
4.19 1.09 1.00 1.00 0.58 1.02 1.99 1.00 1.00 2.22 5.92 6.02 6.06
f1 (ppm)

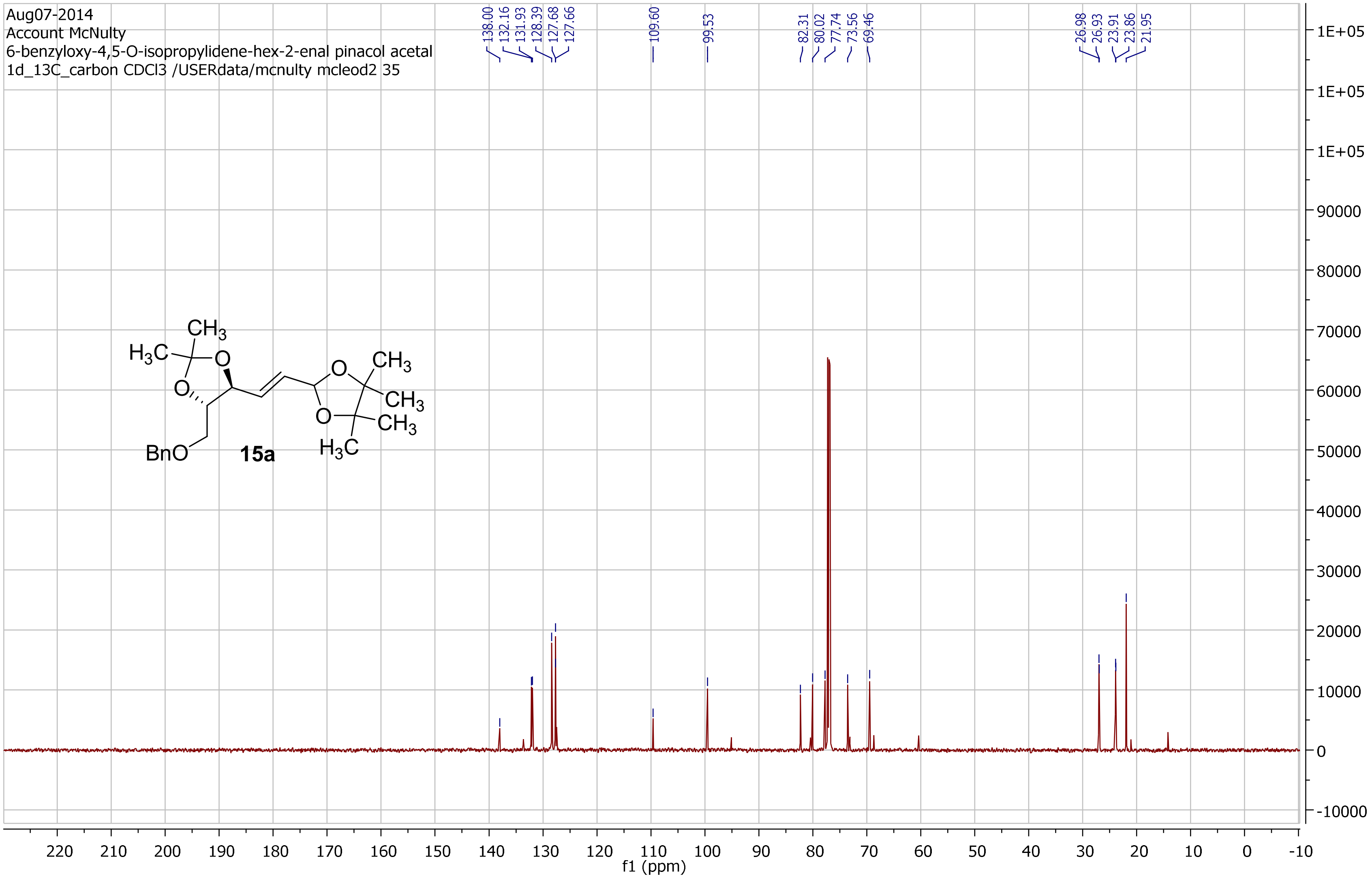

Aug07-2014
Account McNulty
6-benzyloxy-4,5-O-isopropylidene-hex-2-enal pinacol acetal
1d_13C_carbon CDCl3 /USERdata/mcnulty mcleod2 35
138.00
132.16
131.93
128.39
127.68
127.66
109.60
99.53
82.31
80.02
77.74
73.56
69.46
26.98
26.93
23.91
23.86
21.95
15a
f1 (ppm)

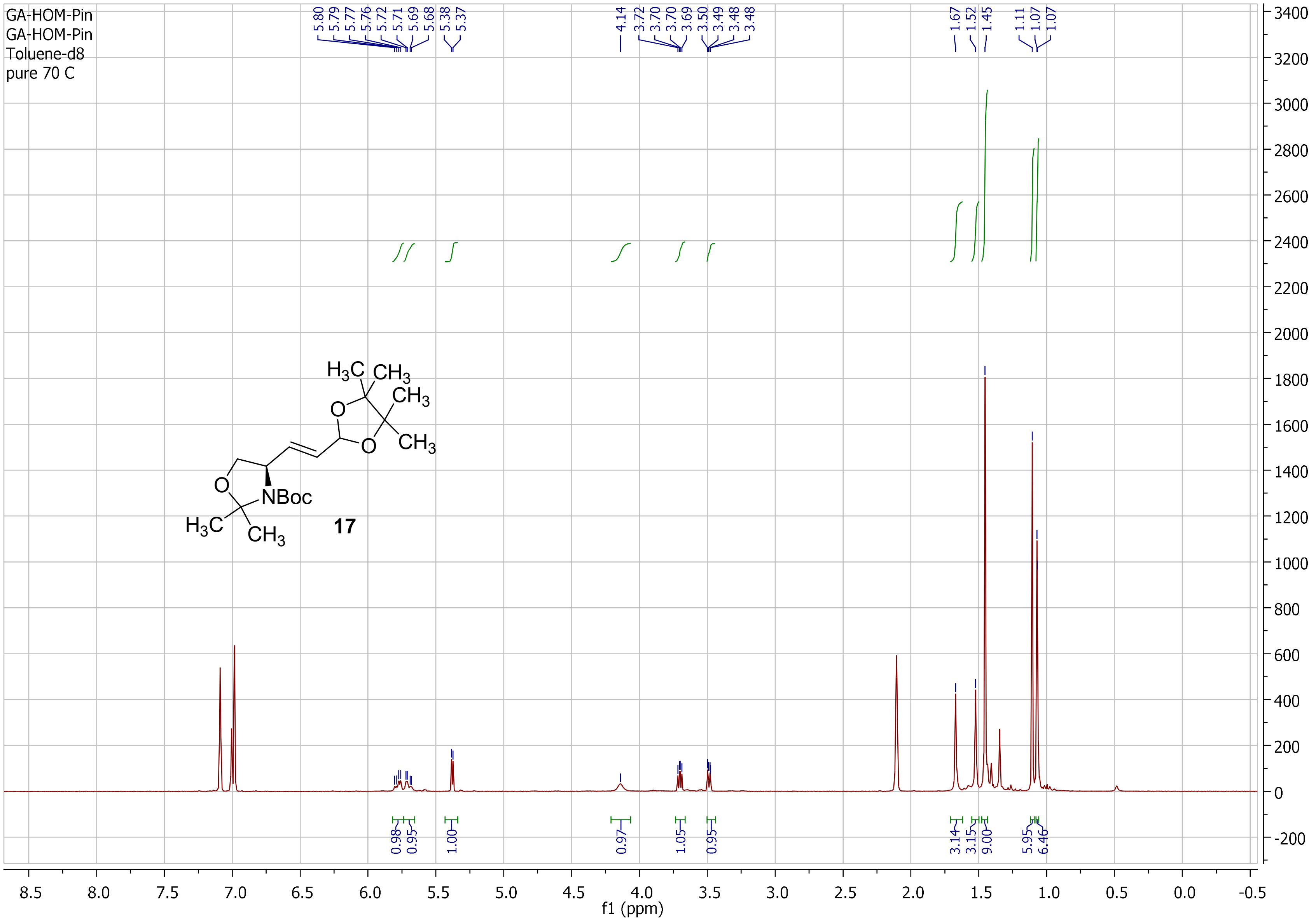
GA-HOM-Pin
GA-HOM-Pin
Toluene-d8
pure 70 C
5.80 5.79 5.77 5.76 5.72 5.71 5.69 5.68 5.38 5.37
4.14 3.72 3.70 3.70 3.69 3.50 3.49 3.48 3.48
1.67 1.52 1.45 1.11 1.07 1.07
0.98 0.95 1.00 0.97 1.05 0.95 3.14 3.15 9.00 5.95 6.46
NBoc
17
f1 (ppm)

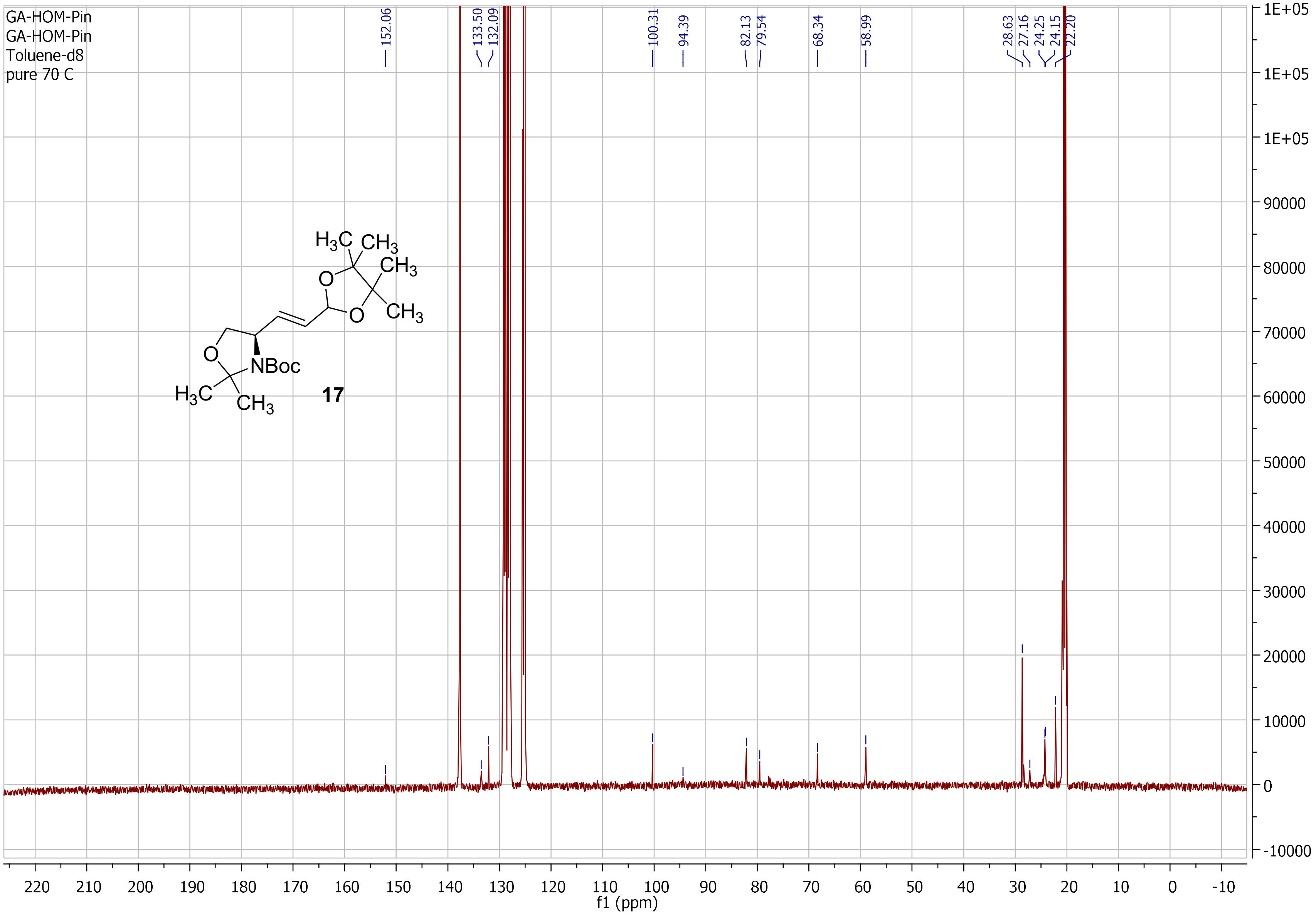

GA-HOM-Pin
GA-HOM-Pin
Toluene-d8
pure 70 C
152.06
133.50
132.09
100.31
94.39
82.13
79.54
68.34
58.99
28.63
27.16
24.25
24.15
22.20
H3C CH3
CH3
CH3
O
O
O
NBoc
H3C
CH3
17
f1 (ppm)

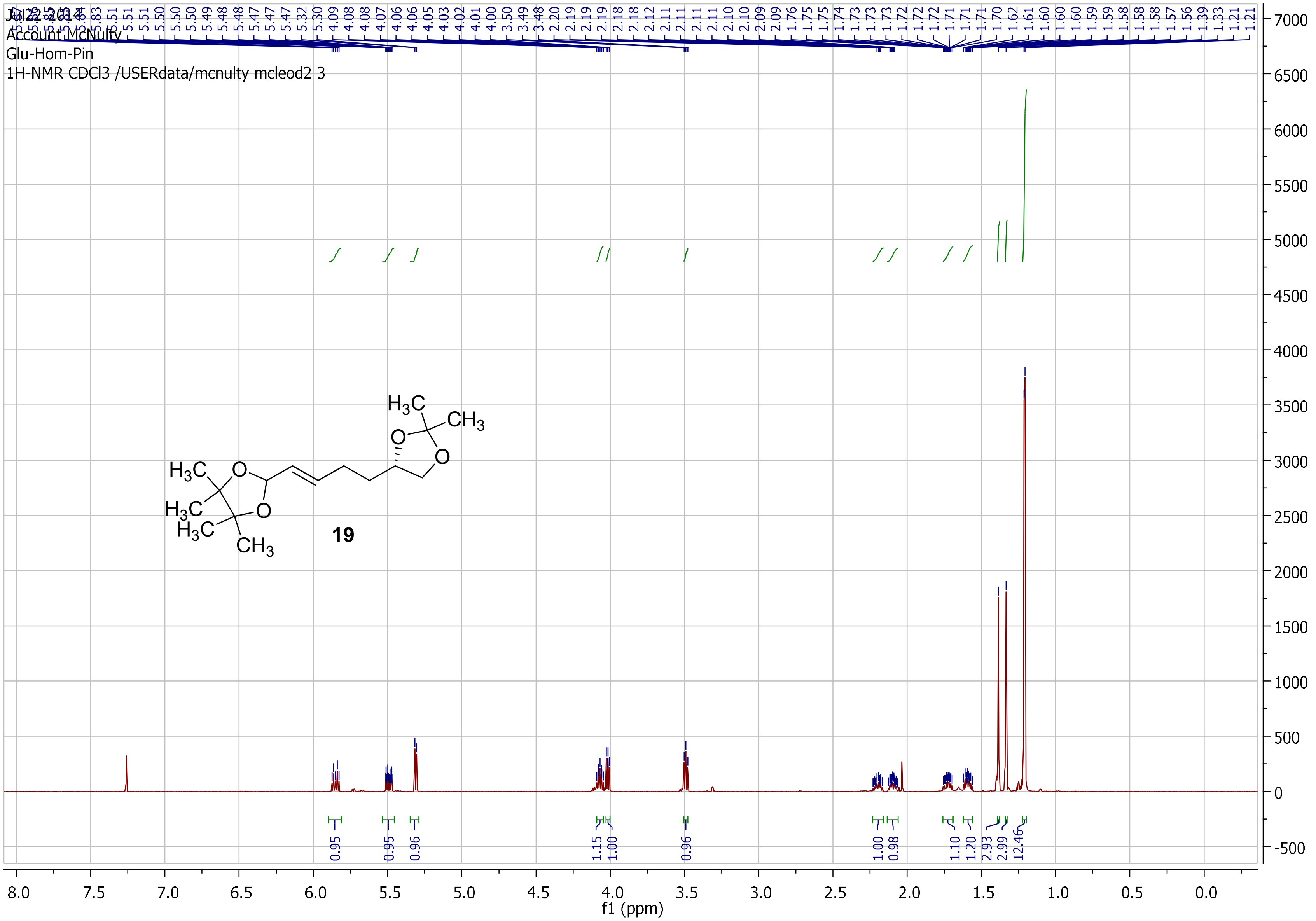

Jul22-2014
Account:McNulty
Glu-Hom-Pin
1H-NMR CDCl3 /USERdata/mcnulty mcleod2 3
19
f1 (ppm)

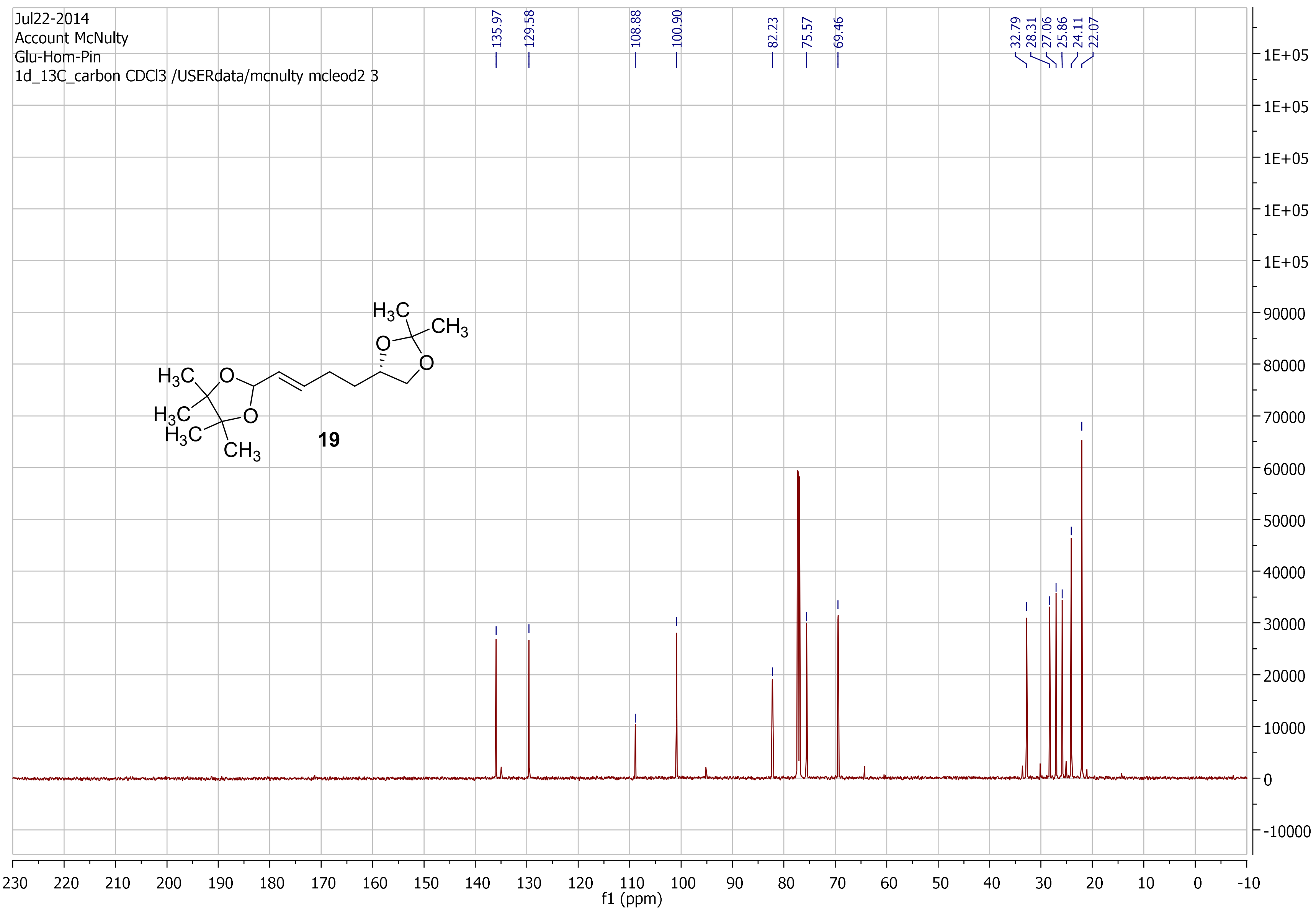
Jul22-2014
Account McNulty
Glu-Hom-Pin
1d_13C_carbon CDCl3 /USERdata/mcnulty mcleod2 3
135.97
129.58
108.88
100.90
82.23
75.57
69.46
32.79
28.31
27.06
25.86
24.11
22.07
19
f1 (ppm)

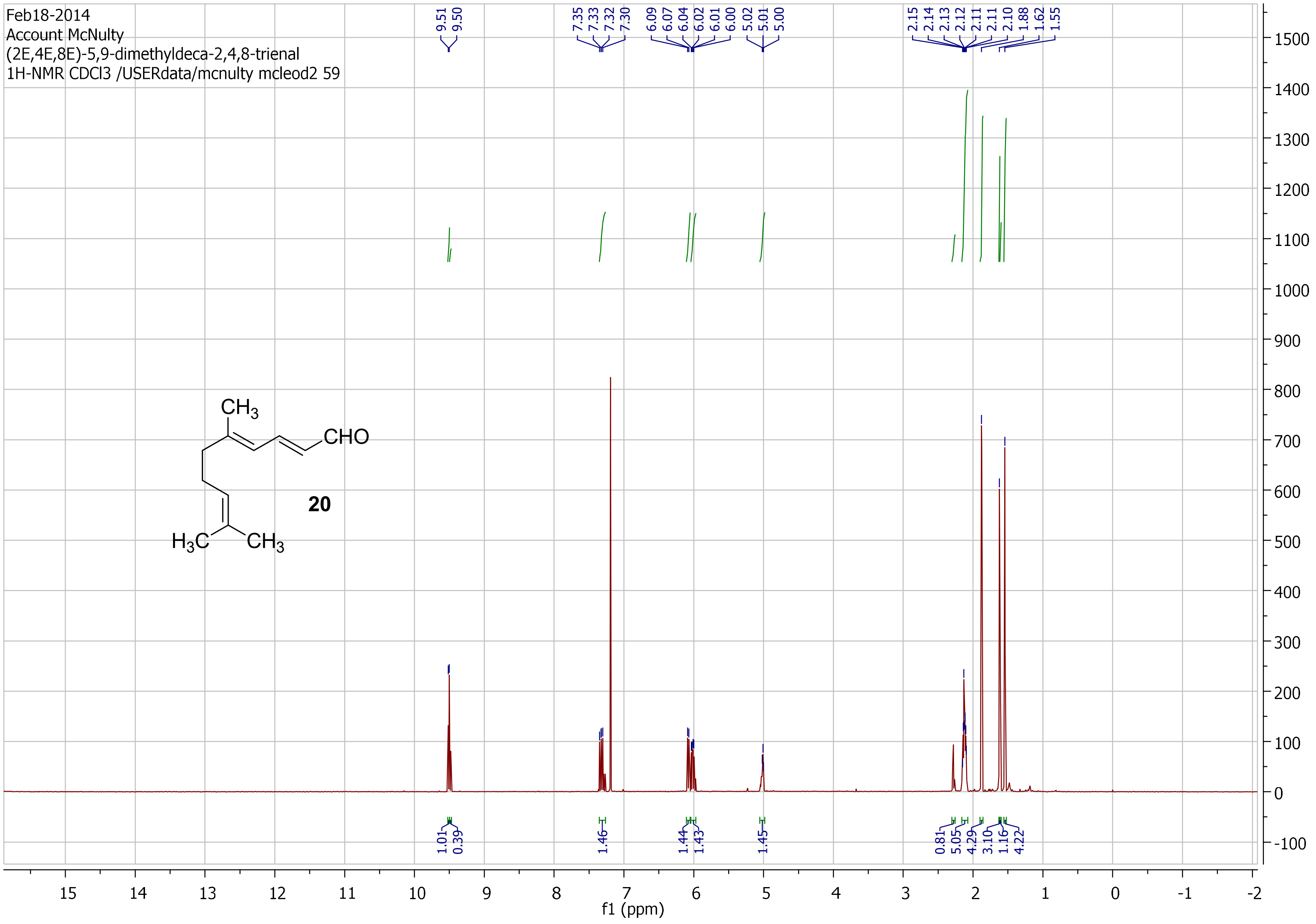
Feb18-2014
Account McNulty
(2E,4E,8E)-5,9-dimethyldeca-2,4,8-trienal
1H-NMR CDCl3 /USERdata/mcnulty mcleod2 59
9.51
9.50
7.35
7.33
7.32
7.30
6.09
6.07
6.04
6.02
6.01
6.00
5.02
5.01
5.00
2.15
2.14
2.13
2.12
2.11
2.11
2.10
1.88
1.62
1.55
CH3
CHO
20
H3C
CH3
1.01
0.39
1.46
1.44
1.43
1.45
0.81
5.05
4.29
3.10
1.16
4.22
f1 (ppm)

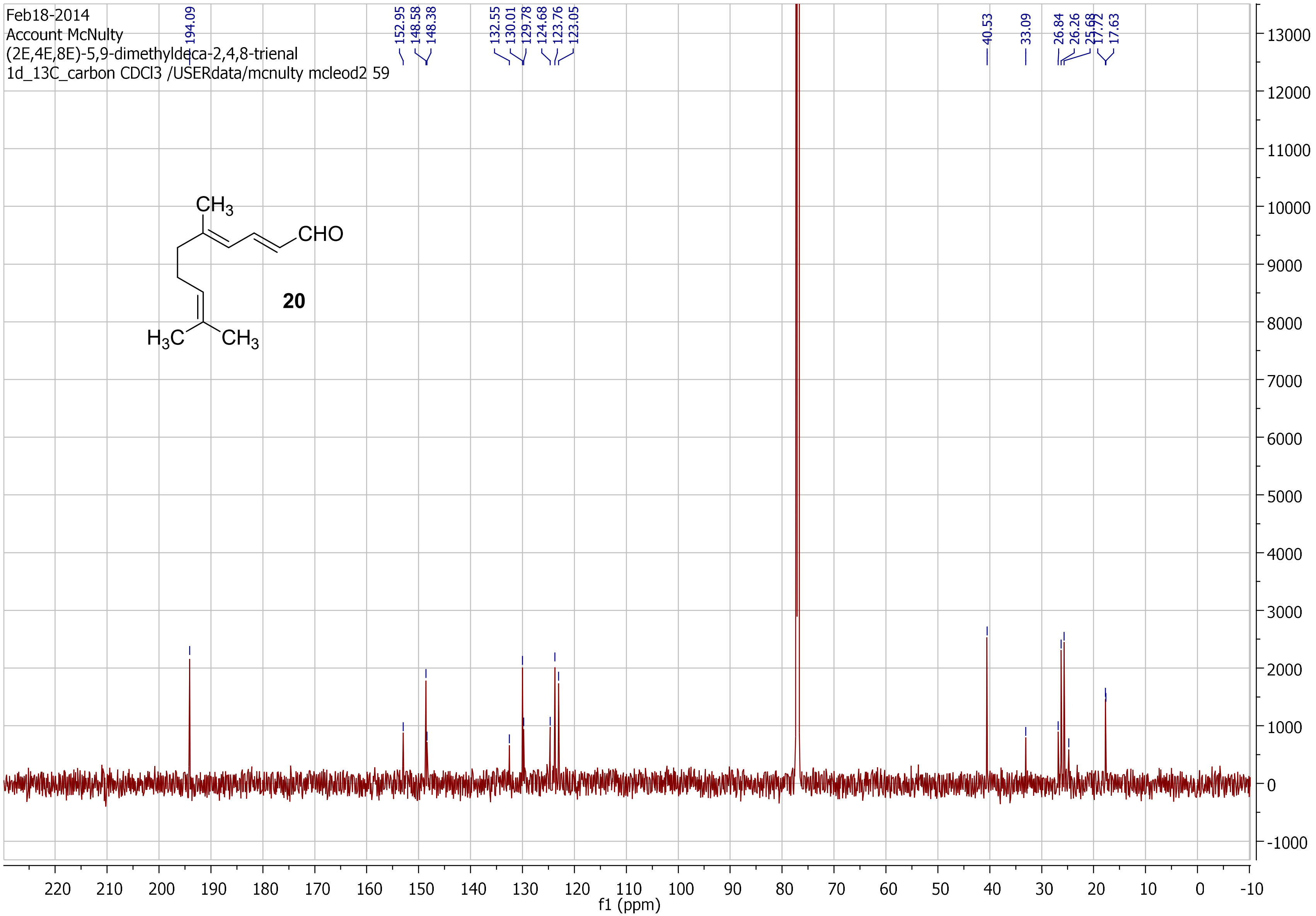
Feb18-2014
Account McNulty
(2E,4E,8E)-5,9-dimethyldeca-2,4,8-trienal
1d_13C_carbon CDCl3 /USERdata/mcnulty mcleod2 59
194.09
152.95
148.58
148.38
132.55
130.01
129.78
124.68
123.76
123.05
40.53
33.09
26.84
26.26
25.68
17.72
17.63
CH3
CHO
20
H3C
CH3
f1 (ppm)

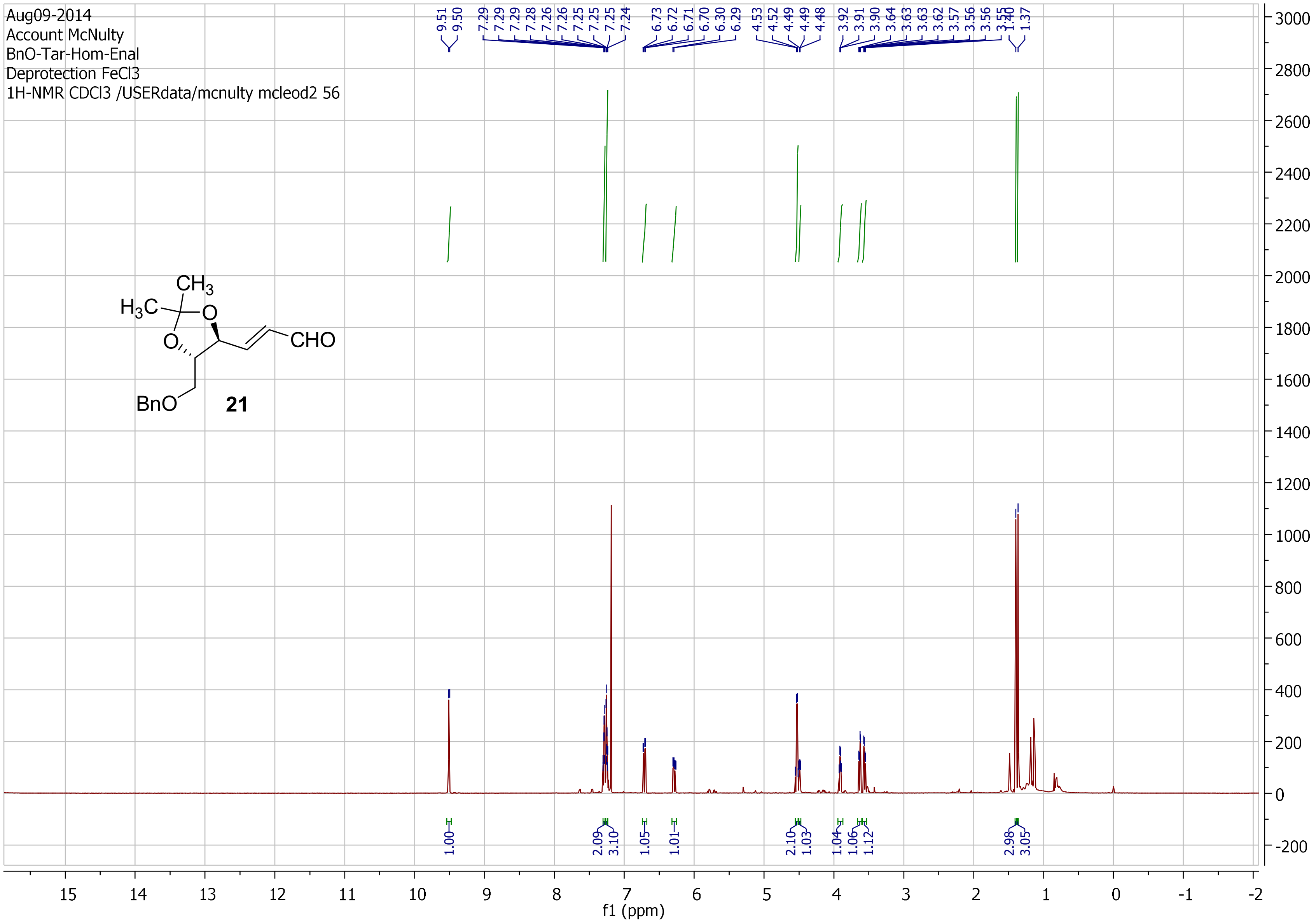

Aug09-2014
Account McNulty
BnO-Tar-Hom-Enal
Deprotection FeCl3
1H-NMR CDCl3 /USERdata/mcnulty mcleod2 56
CHO
BnO
21
f1 (ppm)

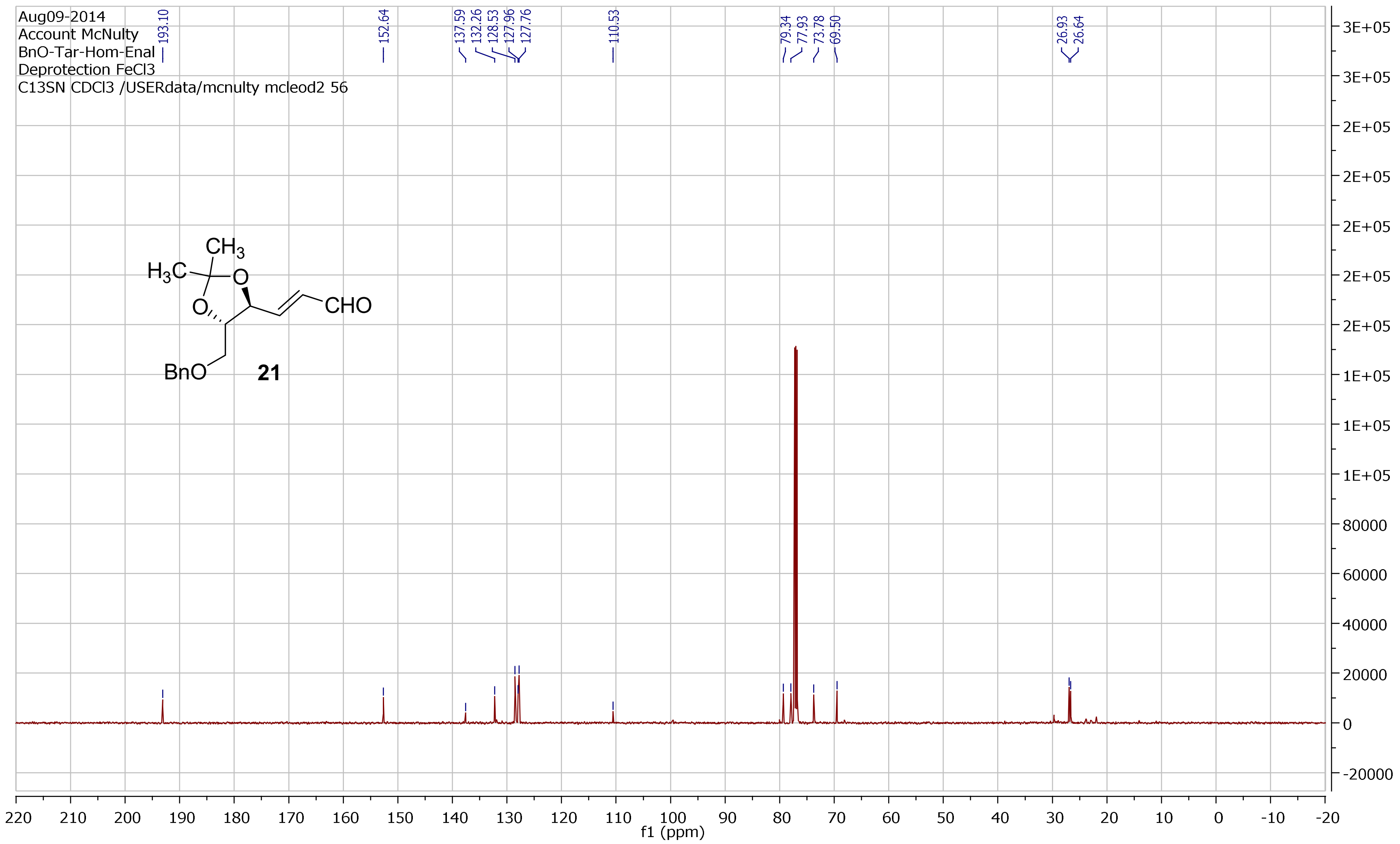
Aug09-2014
Account McNulty
BnO-Tar-Hom-Enal
Deprotection FeCl3
C13SN CDCl3 /USERdata/mcnulty mcleod2 56
193.10
152.64
137.59
132.26
128.53
127.96
127.76
110.53
79.34
77.93
73.78
69.50
26.93
26.64
CH3
H3C
O
O
CHO
BnO
21
f1 (ppm)

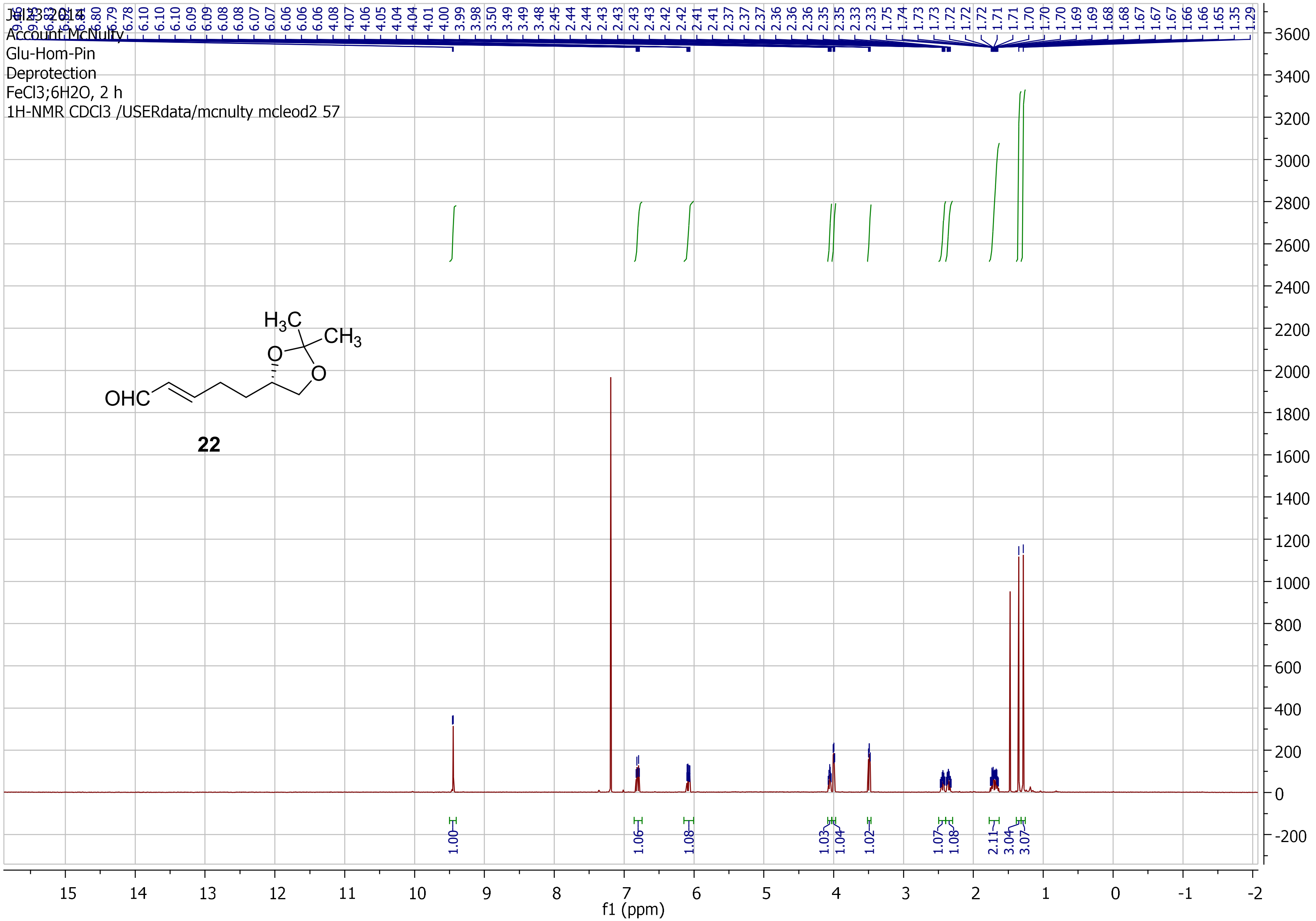
Account:McNulty
Glu-Hom-Pin
Deprotection
FeCl3;6H2O, 2 h
1H-NMR CDCl3 /USERdata/mcnulty mcleod2 57
H3C
CH3
O
O
OHC
22
f1 (ppm)

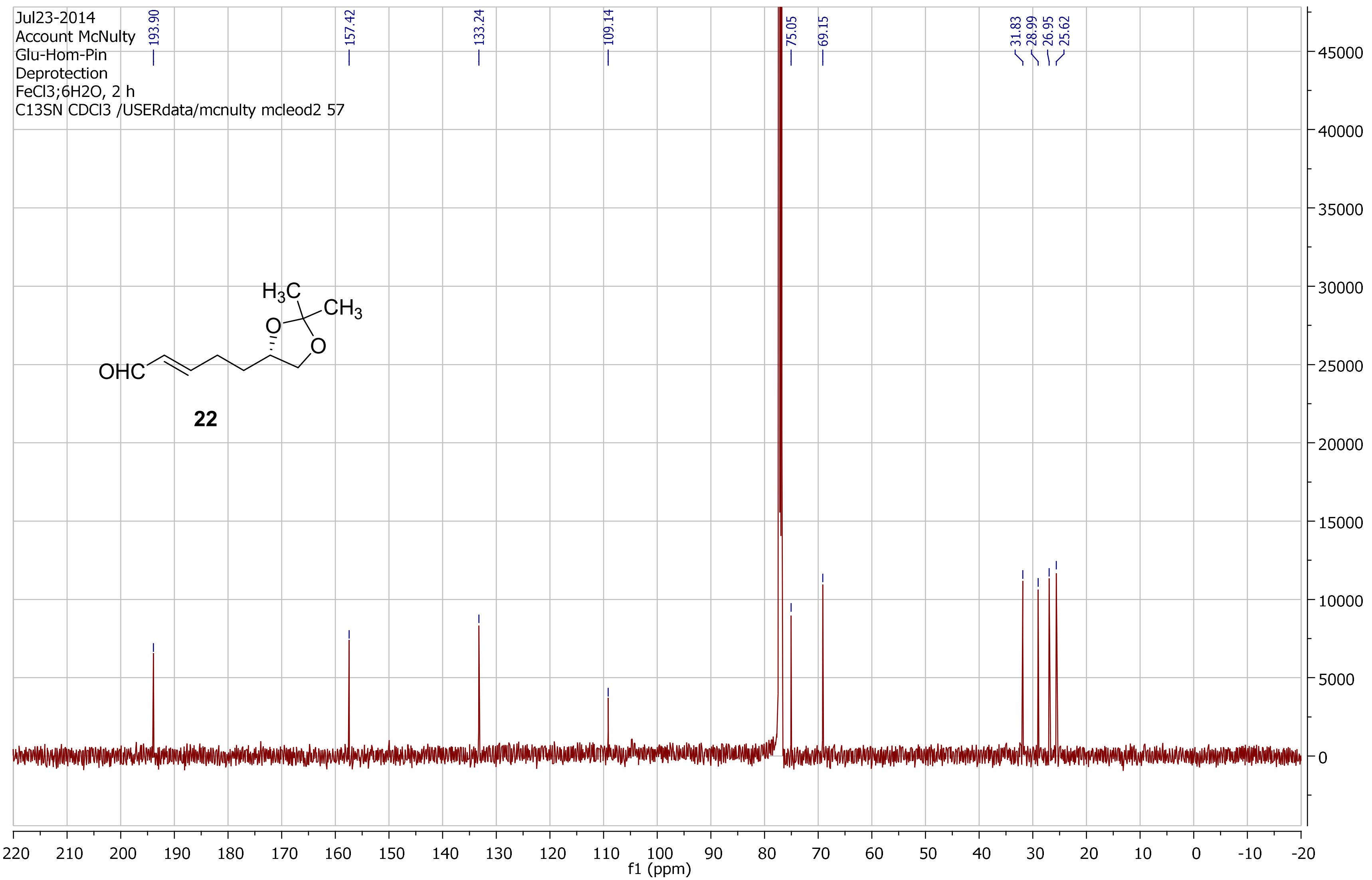
Jul23-2014
Account McNulty
Glu-Hom-Pin
Deprotection
FeCl3;6H2O, 2 h
C13SN CDCl3 /USERdata/mcnulty mcleod2 57
193.90
157.42
133.24
109.14
75.05
69.15
31.83
28.99
26.95
25.62
OHC
O
O
H3C
CH3
22
f1 (ppm)

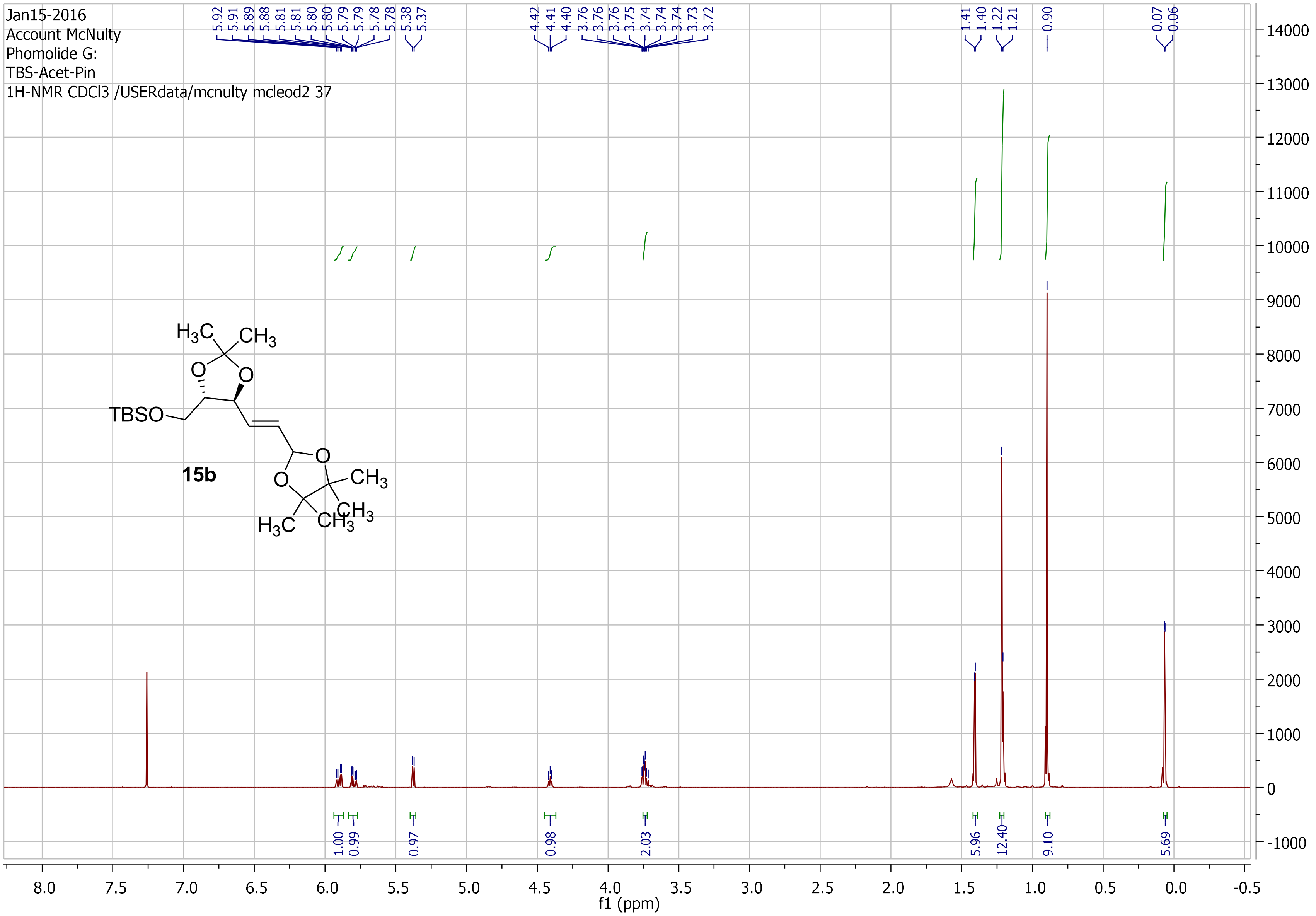
Jan15-2016
Account McNulty
Phomolide G:
TBS-Acet-Pin
1H-NMR CDCl3 /USERdata/mcnulty mcleod2 37
5.92 5.91 5.89 5.88 5.81 5.81 5.80 5.80 5.79 5.79 5.78 5.78 5.38 5.37
4.42 4.41 4.40 3.76 3.76 3.76 3.75 3.74 3.74 3.74 3.73 3.72
1.41 1.40 1.22 1.21 0.90 0.07 0.06
TBSO
15b
1.00 0.99 0.97 0.98 2.03 5.96 12.40 9.10 5.69
f1 (ppm)

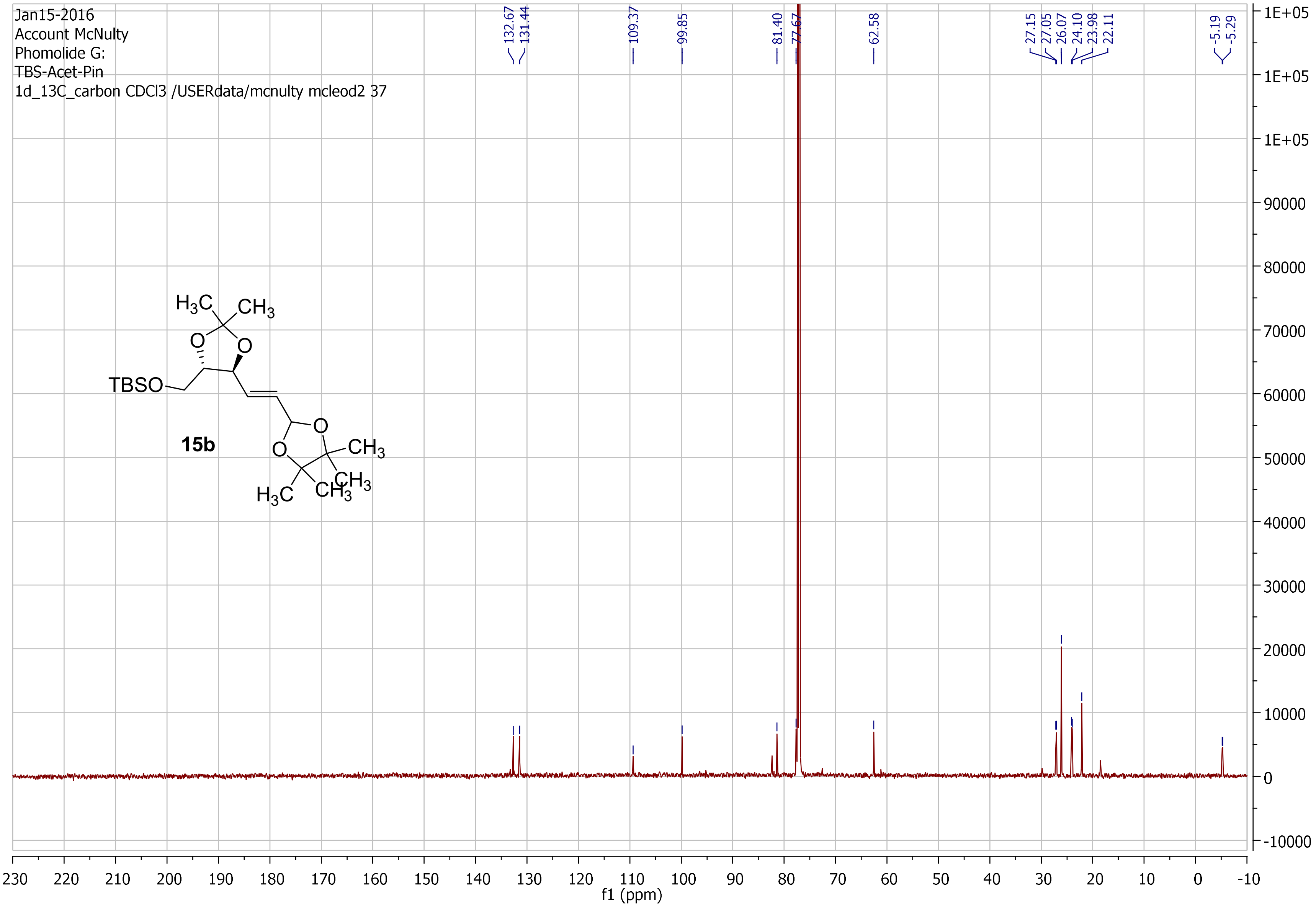
Jan15-2016
Account McNulty
Phomolide G:
TBS-Acet-Pin
1d_13C_carbon CDCl3 /USERdata/mcnulty mcleod2 37
132.67
131.44
109.37
99.85
81.40
77.67
62.58
27.15
27.05
26.07
24.10
23.98
22.11
-5.19
-5.29
TBSO
15b
f1 (ppm)

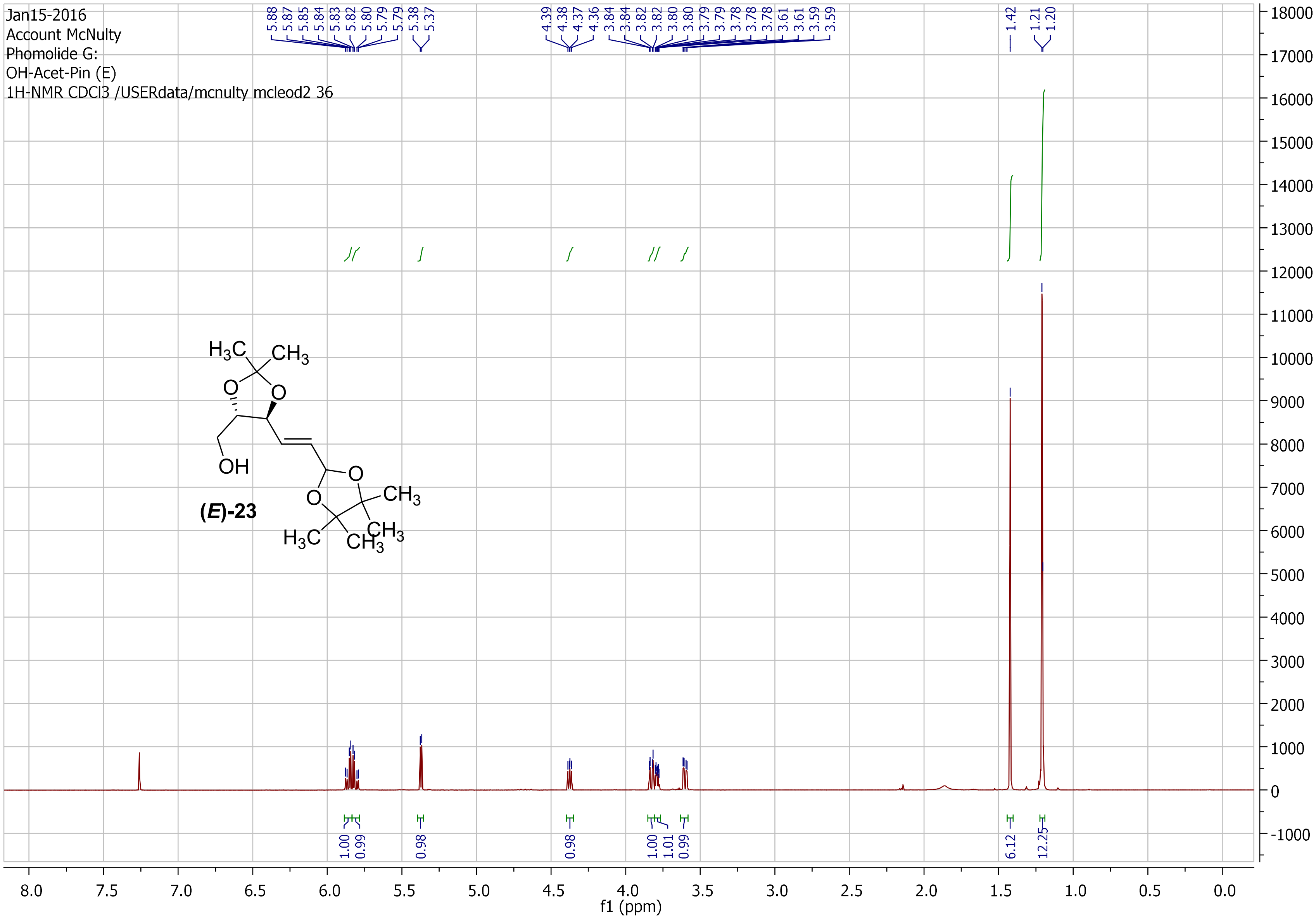
Jan15-2016
Account McNulty
Phomolide G:
OH-Acet-Pin (E)
1H-NMR CDCl3 /USERdata/mcnulty mcleod2 36
(E)-23
f1 (ppm)

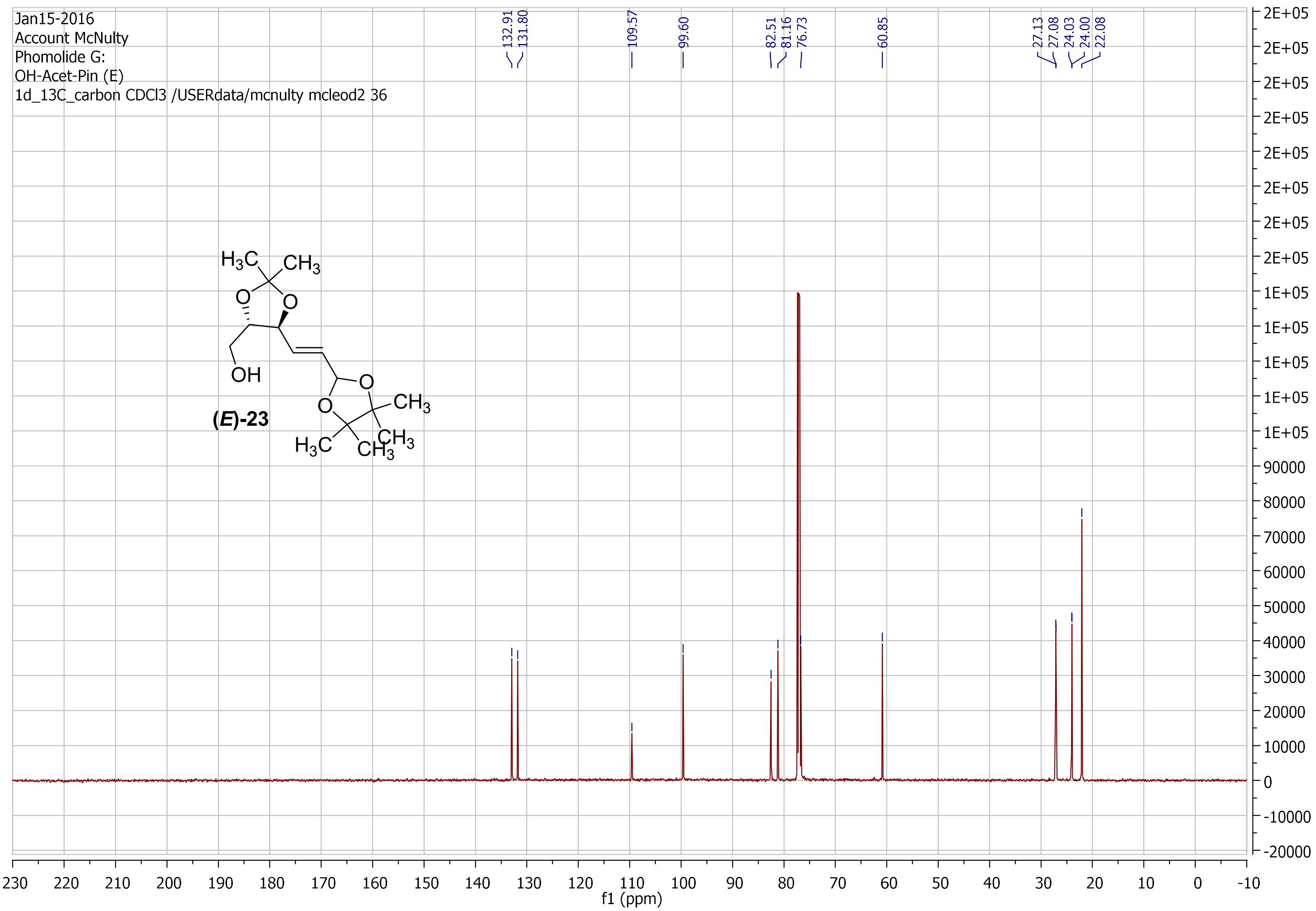
Jan15-2016
Account McNulty
Phomolide G:
OH-Acet-Pin (E)
1d_13C_carbon CDCl3 /USERdata/mcnulty mcleod2 36
132.91
131.80
109.57
99.60
82.51
81.16
76.73
60.85
27.13
27.08
24.03
24.00
22.08
(E)-23
f1 (ppm)

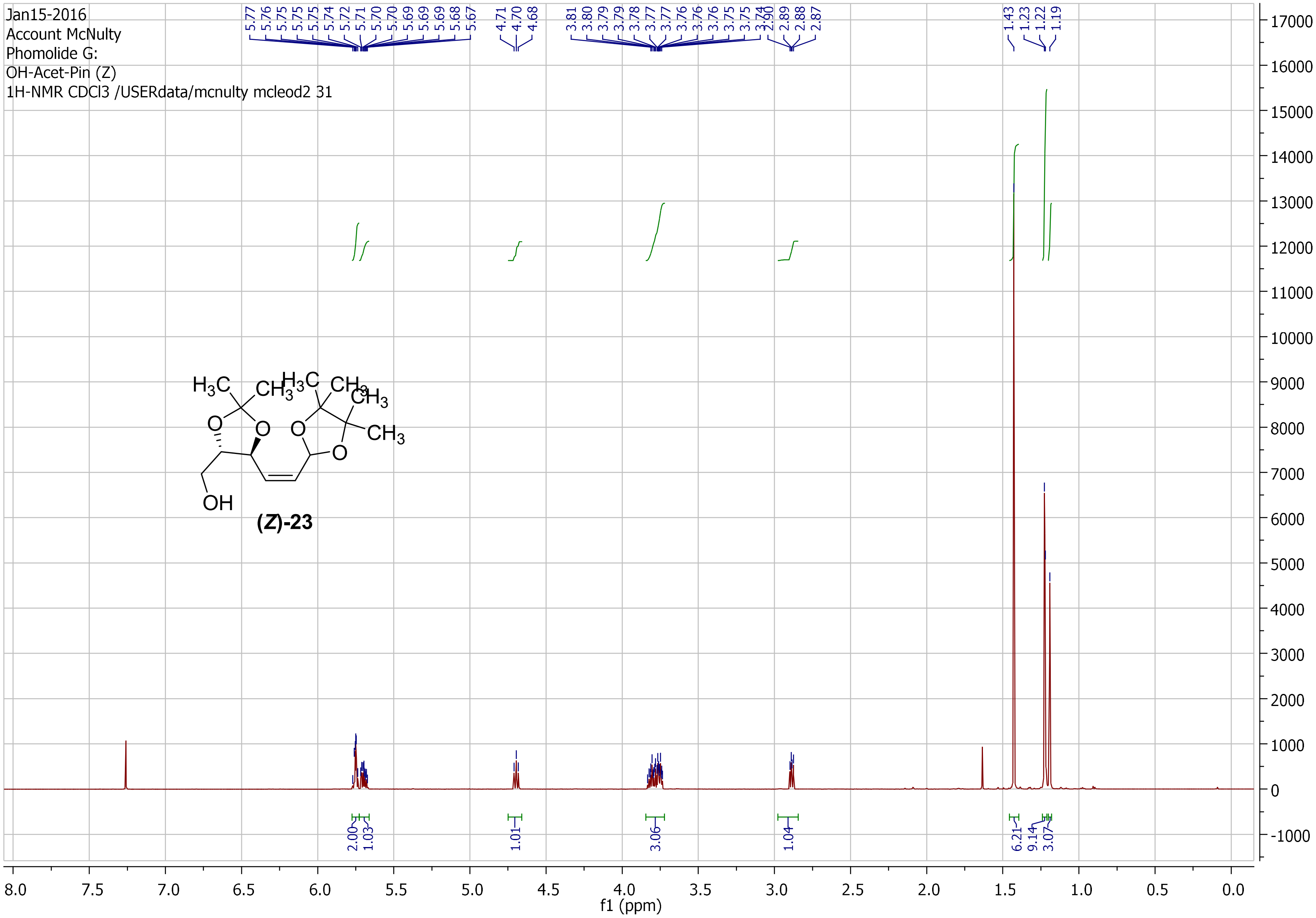
Jan15-2016
Account McNulty
Phomolide G:
OH-Acet-Pin (Z)
1H-NMR CDCl3 /USERdata/mcnulty mcleod2 31
5.77 5.76 5.75 5.75 5.75 5.74 5.72 5.71 5.70 5.70 5.69 5.69 5.69 5.68 5.67
4.71 4.70 4.68
3.81 3.80 3.79 3.79 3.78 3.77 3.77 3.76 3.76 3.75 3.75 3.74
2.90 2.89 2.88 2.87
1.43 1.23 1.22 1.19
2.00 1.03 1.01 3.06 1.04 6.21 9.14 3.07
(Z)-23
f1 (ppm)

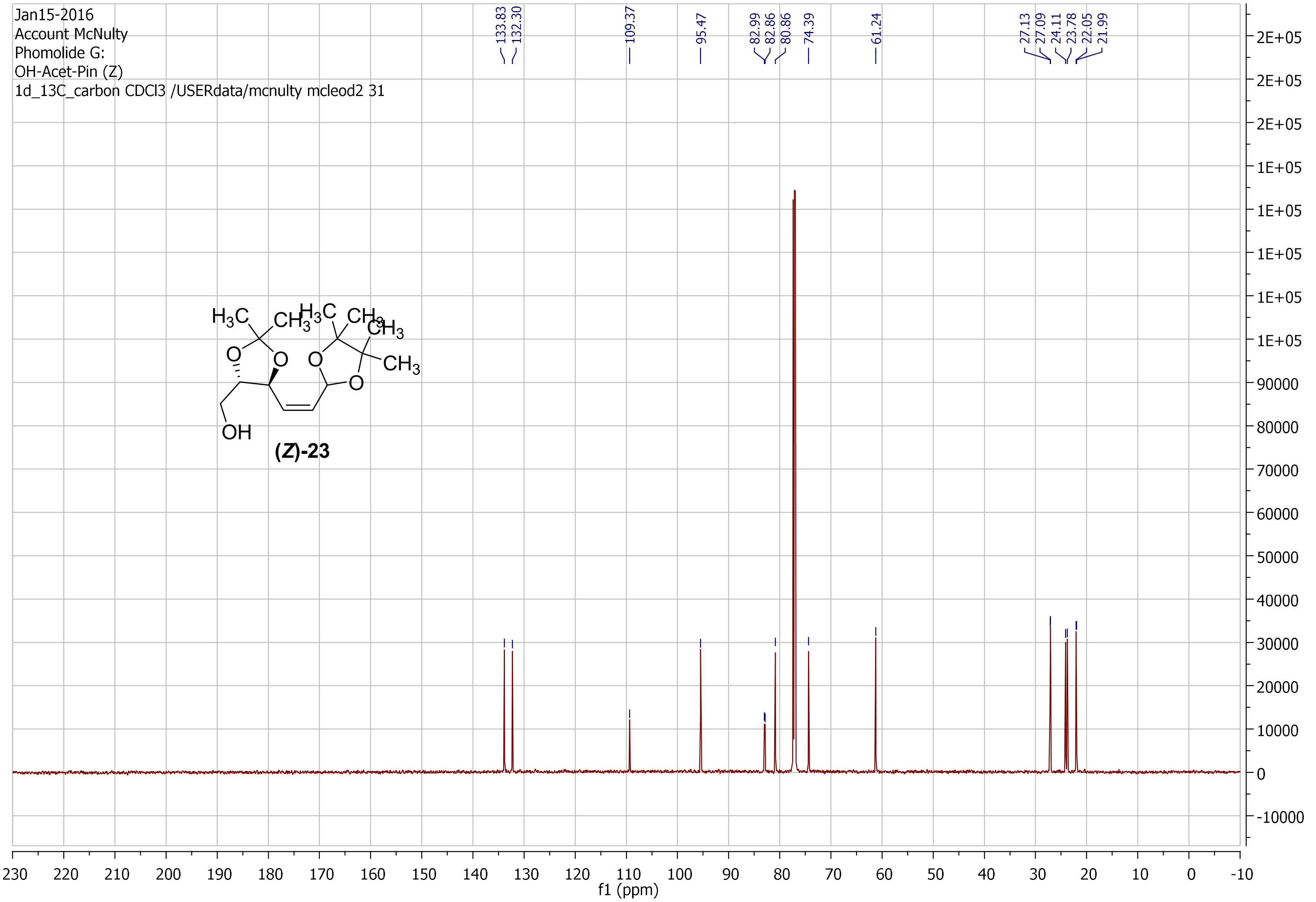
Jan15-2016
Account McNulty
Phomolide G:
OH-Acet-Pin (Z)
1d_13C_carbon CDCl3 /USERdata/mcnulty mcleod2 31
133.83
132.30
109.37
95.47
82.99
82.86
80.86
74.39
61.24
27.13
27.09
24.11
23.78
22.05
21.99
(Z)-23
f1 (ppm)

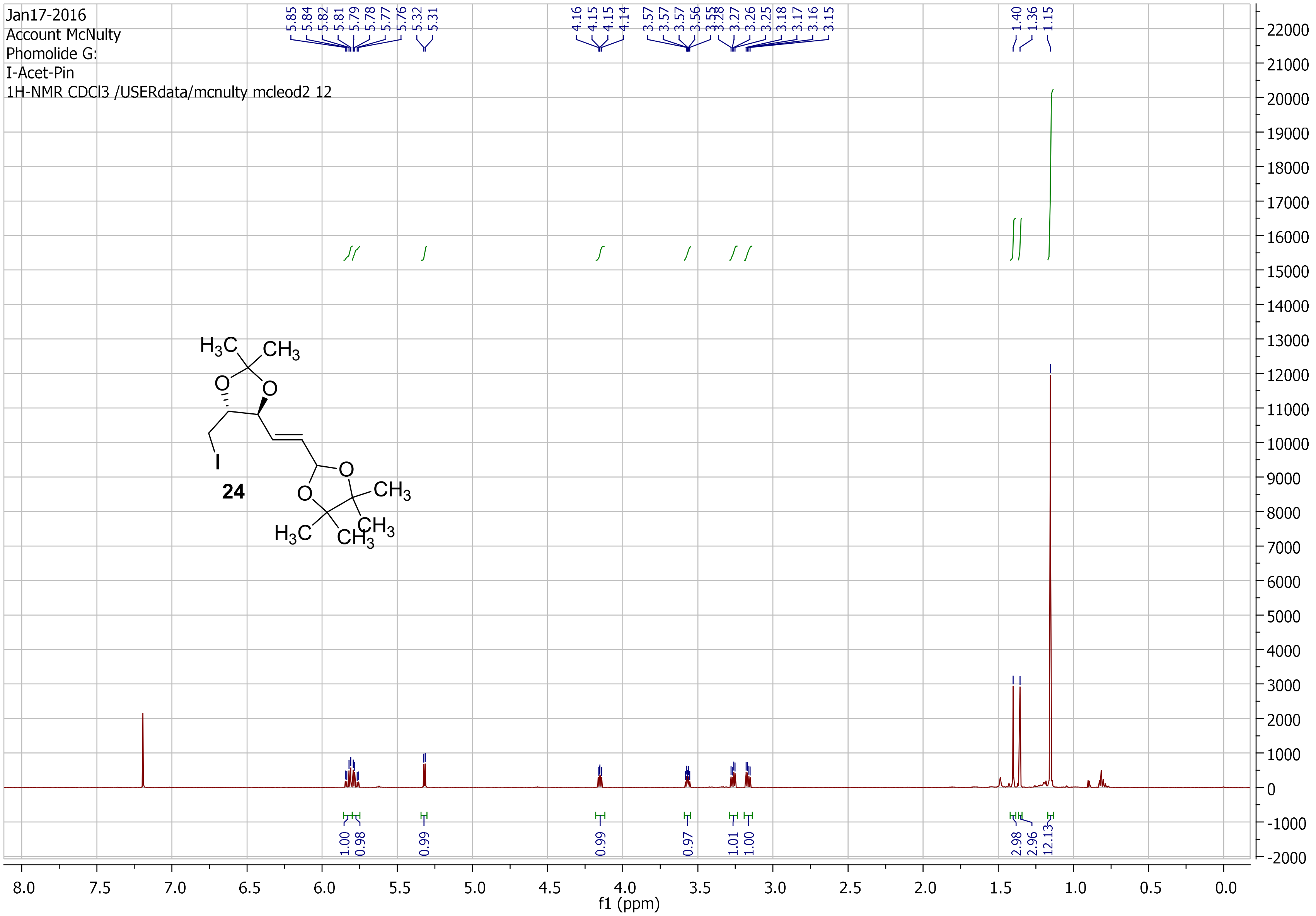
Jan17-2016
Account McNulty
Phomolide G:
I-Acet-Pin
1H-NMR CDCl3 /USERdata/mcnulty mcleod2 12
24
f1 (ppm)

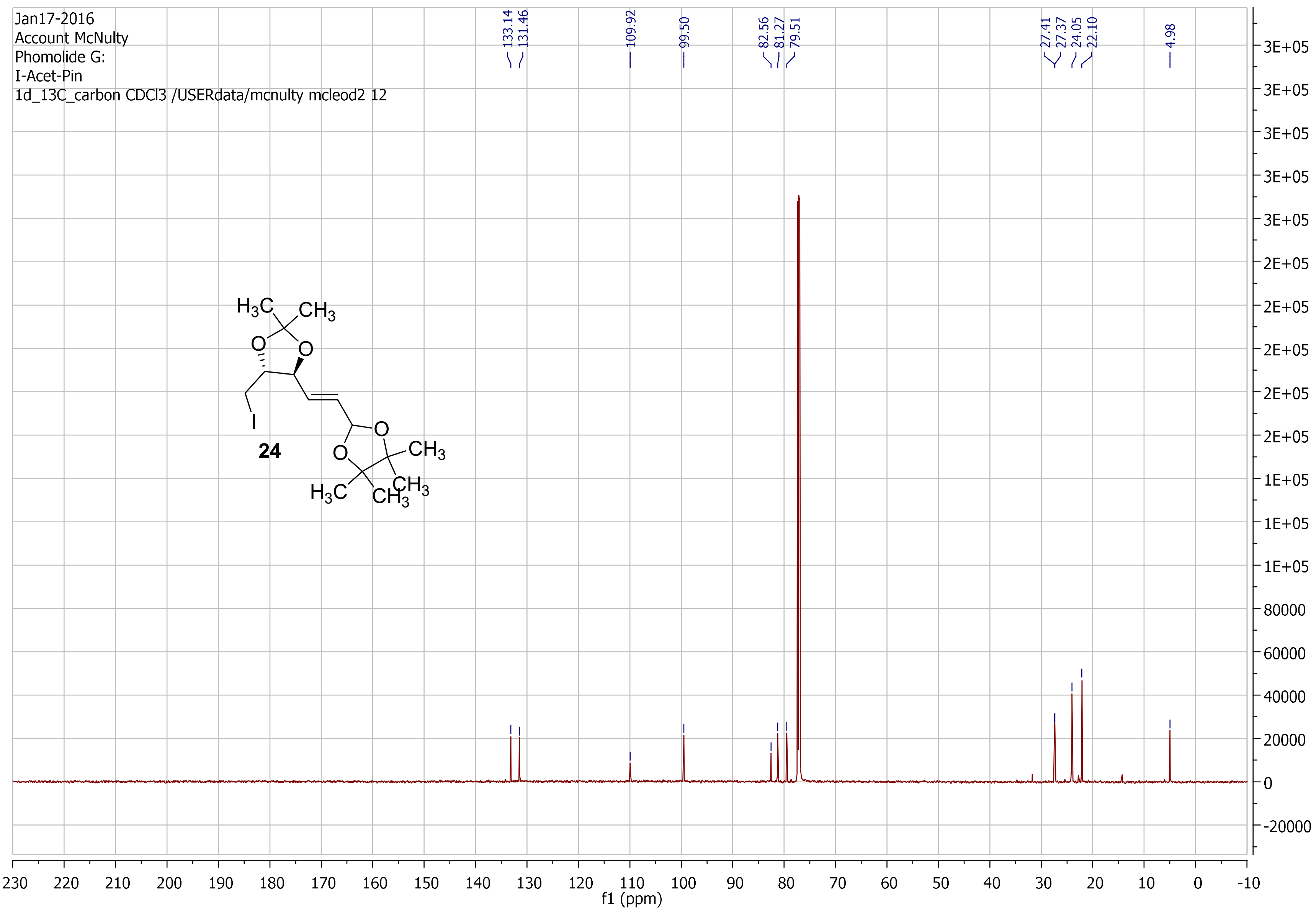
Jan17-2016
Account McNulty
Phomolide G:
I-Acet-Pin
1d_13C_carbon CDCl3 /USERdata/mcnulty mcleod2 12
133.14
131.46
109.92
99.50
82.56
81.27
79.51
27.41
27.37
24.05
22.10
4.98
24
f1 (ppm)

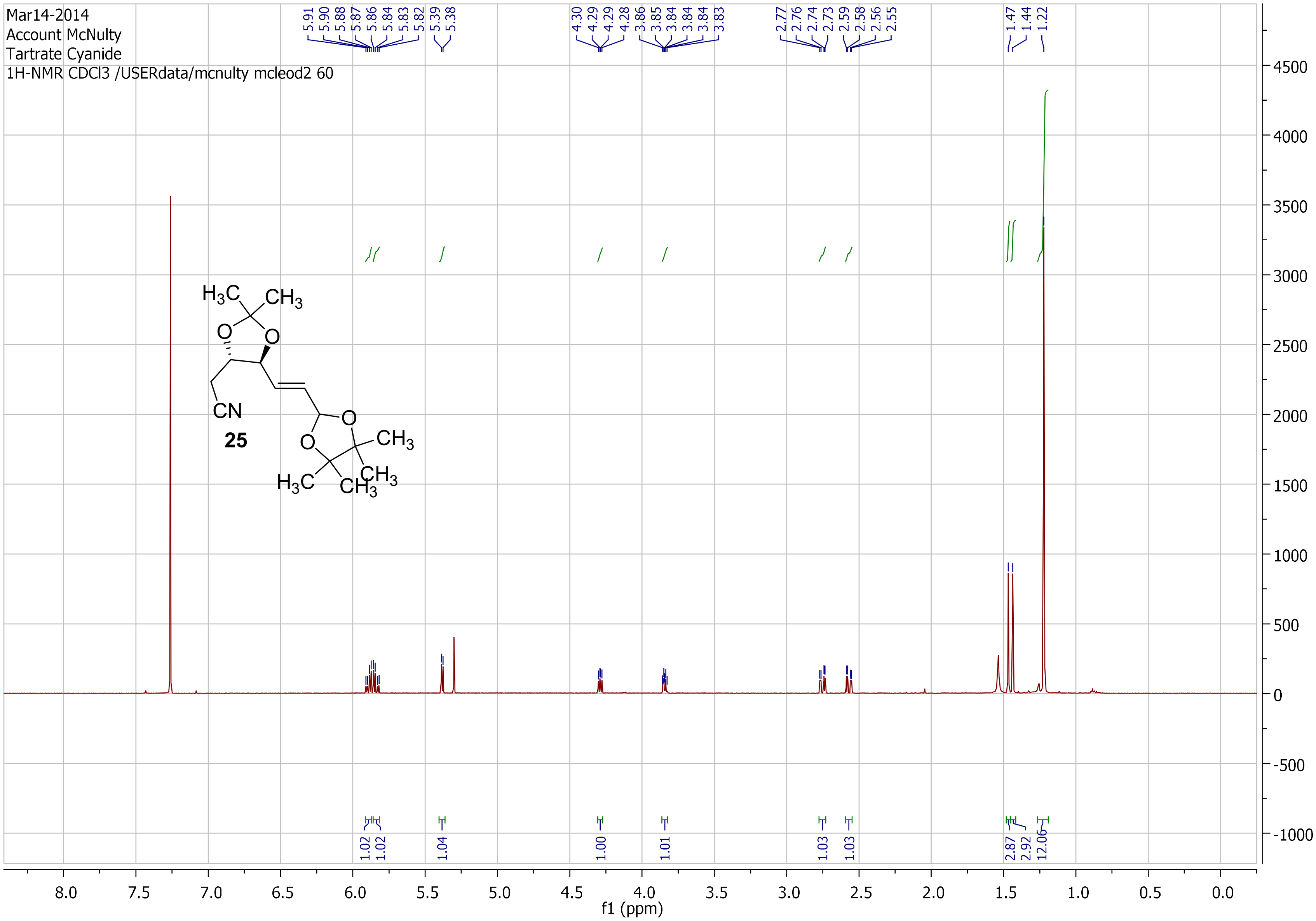
Mar14-2014
Account McNulty
Tartrate Cyanide
1H-NMR CDCl3 /USERdata/mcnulty mcleod2 60
5.91 5.90 5.88 5.87 5.86 5.84 5.83 5.82 5.39 5.38
4.30 4.29 4.29 4.28 3.86 3.85 3.84 3.84 3.84 3.83
2.77 2.76 2.74 2.73 2.59 2.58 2.56 2.55
1.47 1.44 1.22
H_3C
CH_3
O
O
CN
25
O
O
CH_3
CH_3
H_3C
CH_3
1.02 1.02 1.04 1.00 1.01 1.03 1.03 2.87 2.92 12.06
f1 (ppm)

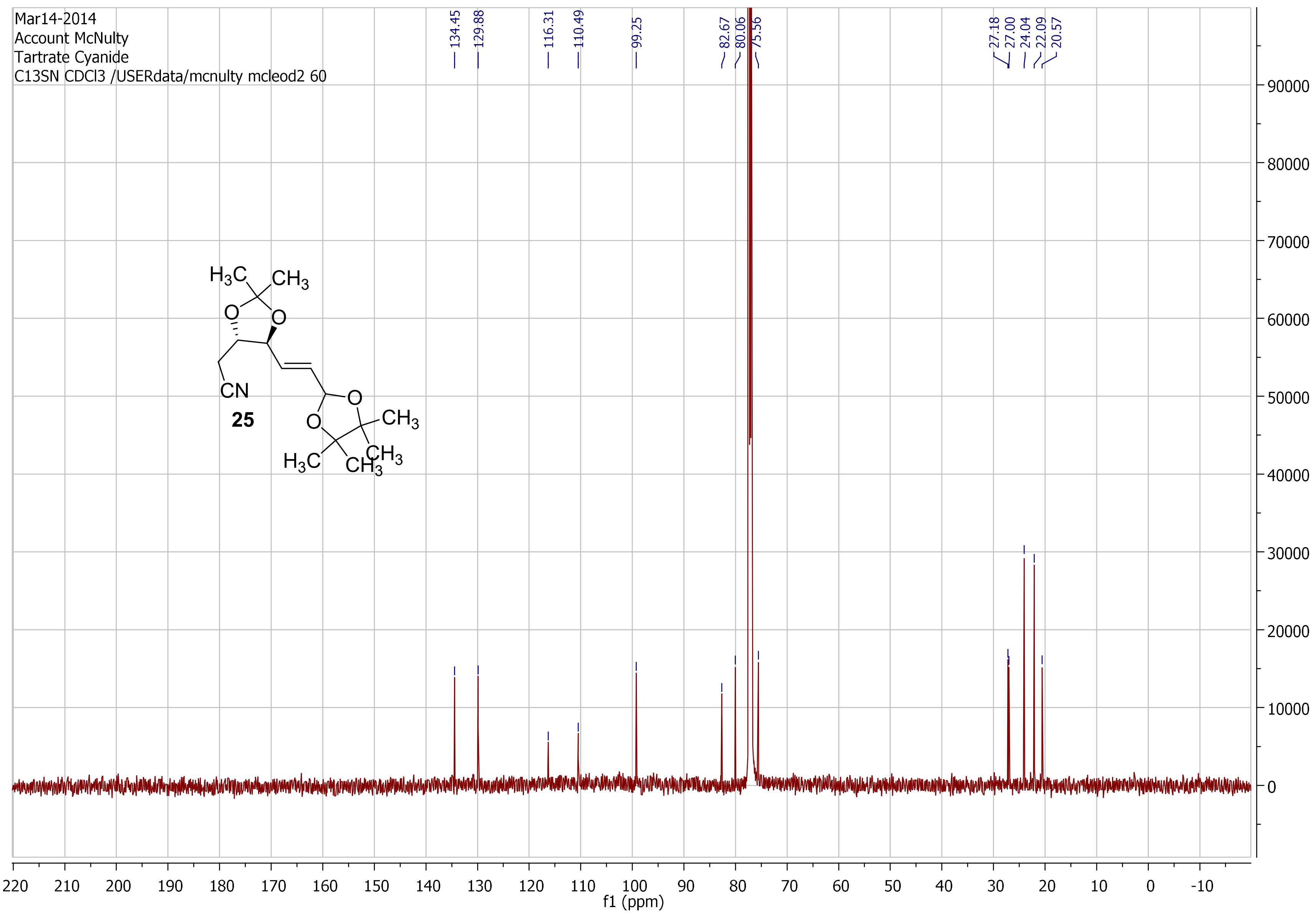
Mar14-2014
Account McNulty
Tartrate Cyanide
C13SN CDCl3 /USERdata/mcnulty mcleod2 60
134.45
129.88
116.31
110.49
99.25
82.67
80.06
75.56
27.18
27.00
24.04
22.09
20.57
25
f1 (ppm)

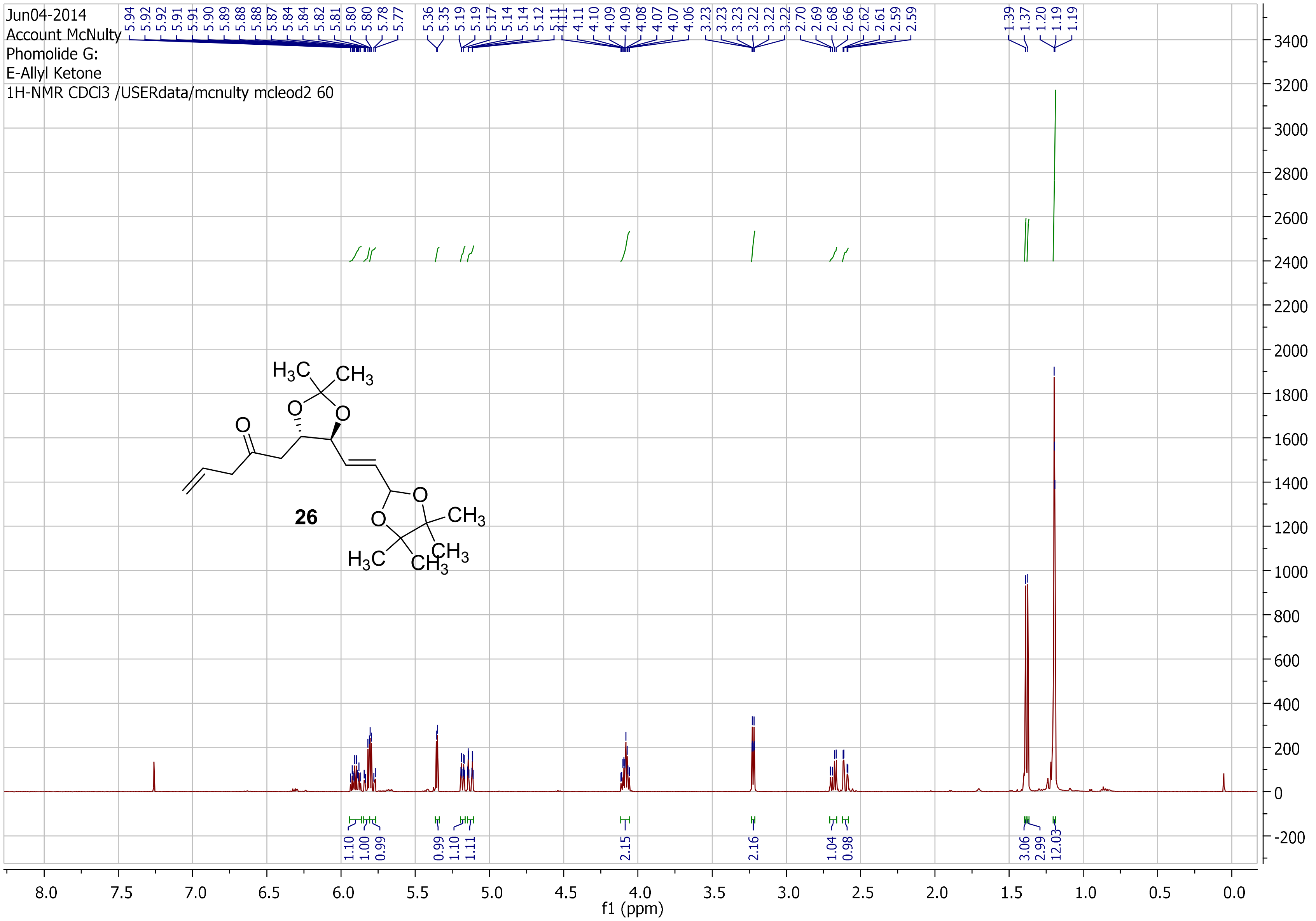
Jun04-2014
Account McNulty
Phomolide G:
E-Allyl Ketone
1H-NMR CDCl3 /USERdata/mcnulty mcleod2 60
5.94 5.92 5.92 5.91 5.91 5.90 5.89 5.88 5.88 5.87 5.84 5.84 5.82 5.81 5.80 5.80 5.78 5.77 5.36 5.35 5.19 5.19 5.17 5.14 5.14 5.12 5.11 4.11 4.10 4.09 4.09 4.08 4.07 4.07 4.06 3.23 3.23 3.23 3.22 3.22 3.22 2.70 2.69 2.68 2.66 2.62 2.61 2.59 2.59 1.39 1.37 1.20 1.19 1.19
1.10 1.00 0.99 0.99 1.10 1.11 2.15 2.16 1.04 0.98 3.06 2.99 12.03
26
f1 (ppm)

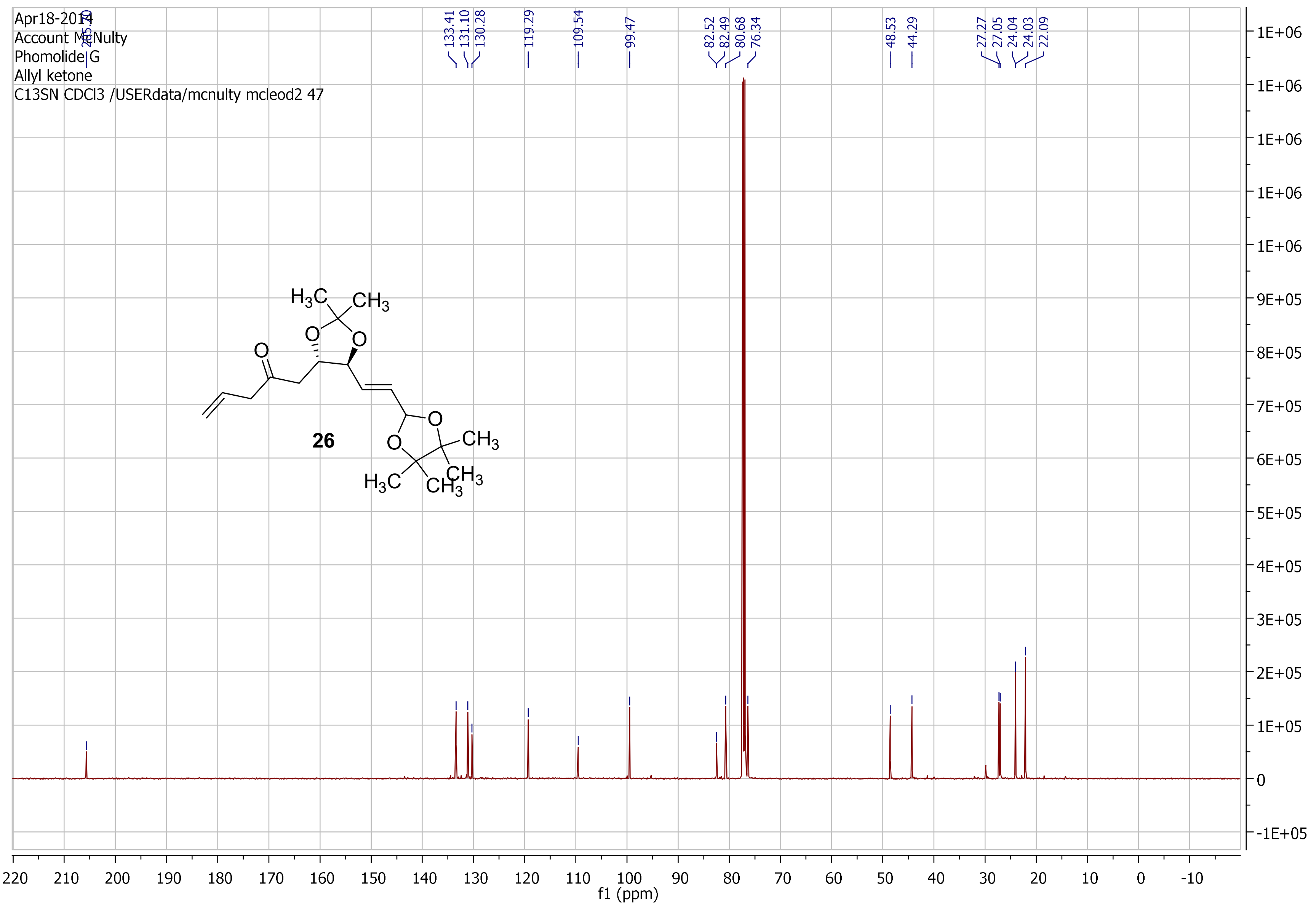

Apr18-2014
Account McNulty
Phomolide G
Allyl ketone
C13SN CDCl3 /USERdata/mcnulty mcleod2 47
205.70
133.41
131.10
130.28
119.29
109.54
99.47
82.52
82.49
80.68
76.34
48.53
44.29
27.27
27.05
24.04
24.03
22.09
H3C
CH3
O
O
O
O
O
CH3
CH3
H3C
CH3
26
1E+06
1E+06
1E+06
1E+06
1E+06
9E+05
8E+05
7E+05
6E+05
5E+05
4E+05
3E+05
2E+05
1E+05
0
-1E+05
220
210
200
190
180
170
160
150
140
130
120
110
100
90
80
70
60
50
40
30
20
10
0
-10
f1 (ppm)

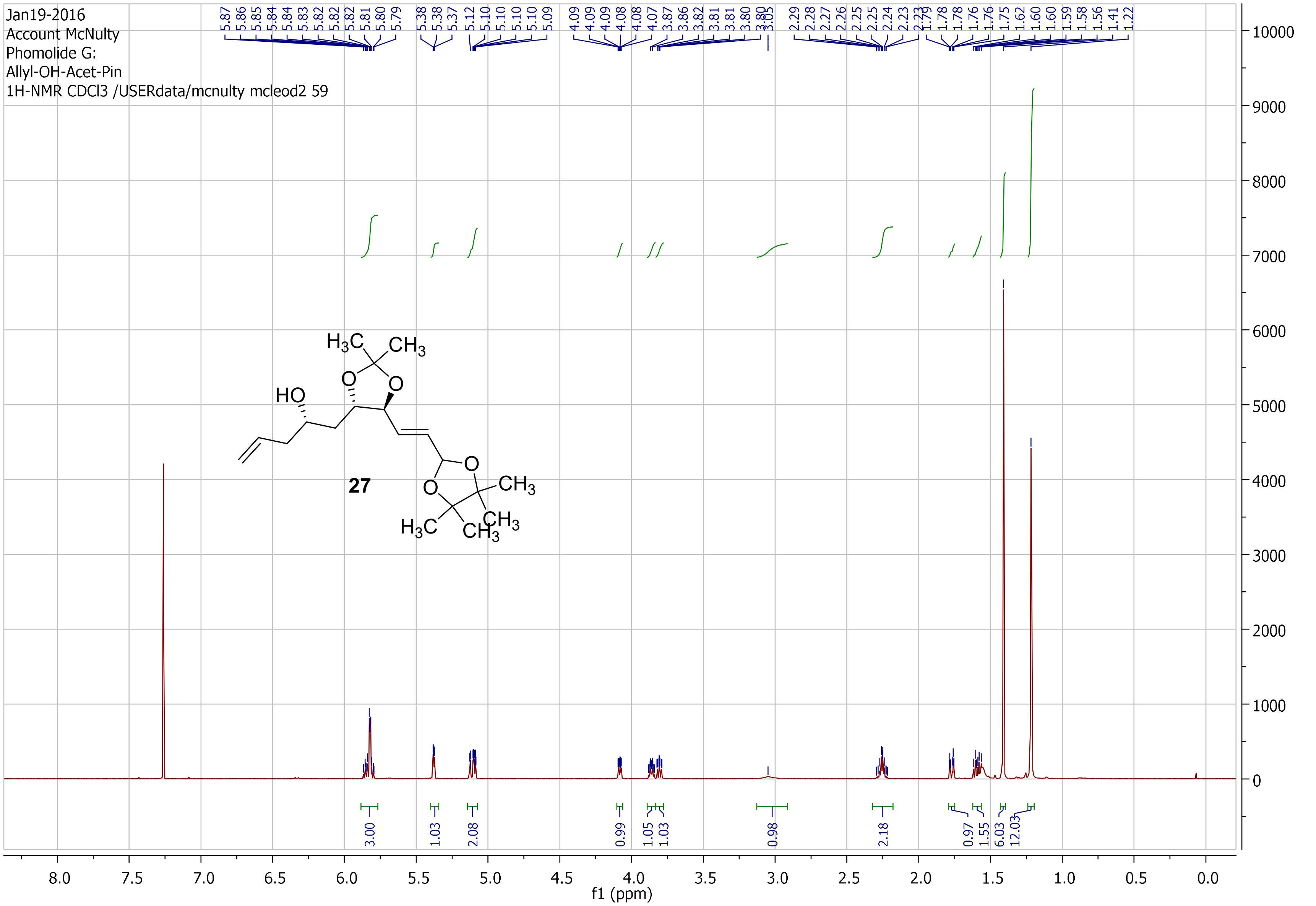

Jan19-2016
Account McNulty
Phomolide G:
Allyl-OH-Acet-Pin
1H-NMR CDCl3 /USERdata/mcnulty mcleod2 59
27
f1 (ppm)

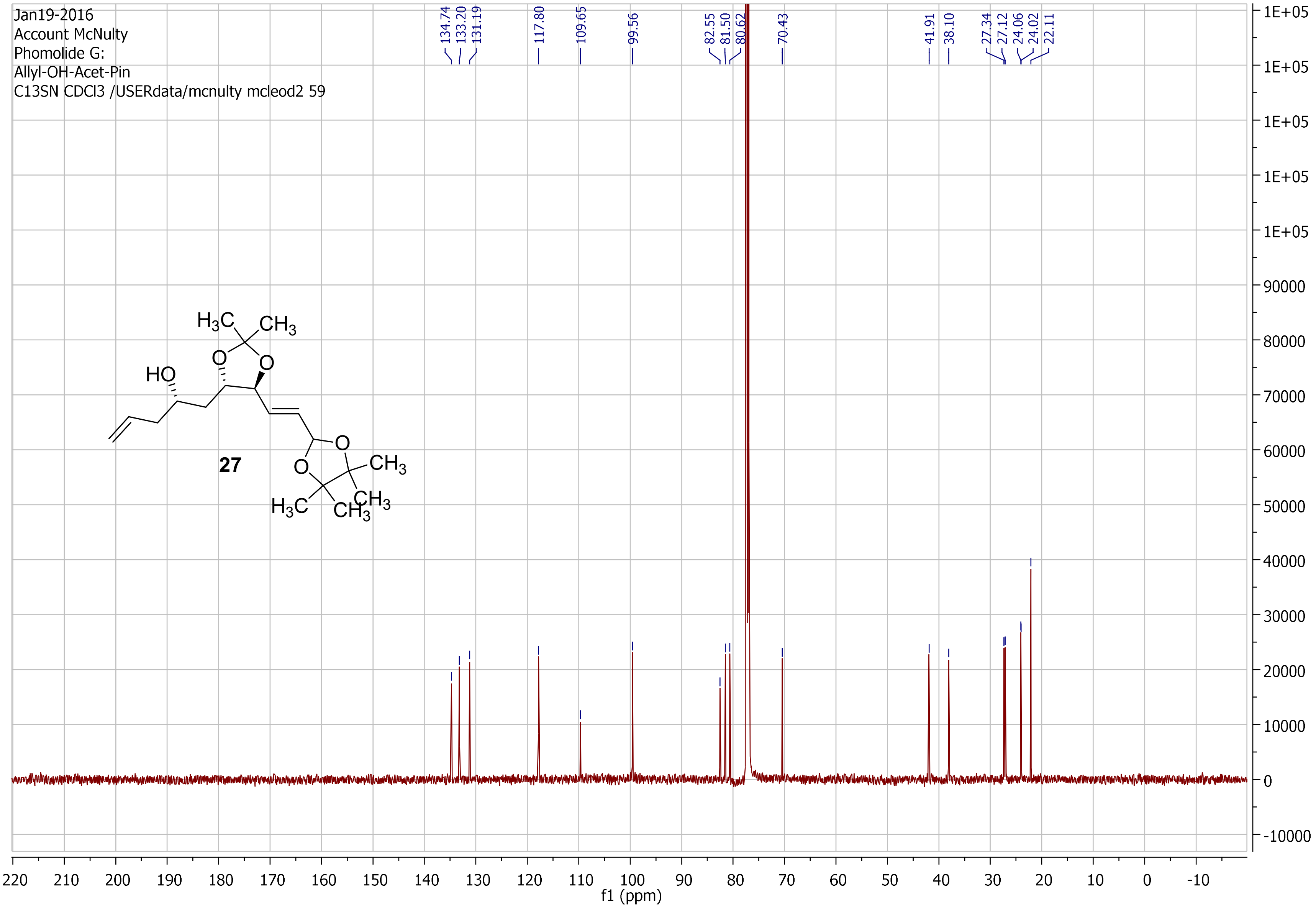
Jan19-2016
Account McNulty
Phomolide G:
Allyl-OH-Acet-Pin
C13SN CDCl3 /USERdata/mcnulty mcleod2 59
134.74
133.20
131.19
117.80
109.65
99.56
82.55
81.50
80.62
70.43
41.91
38.10
27.34
27.12
24.06
24.02
22.11
HO
O
O
H3C
CH3
CH3
CH3
CH3
H3C
27
f1 (ppm)

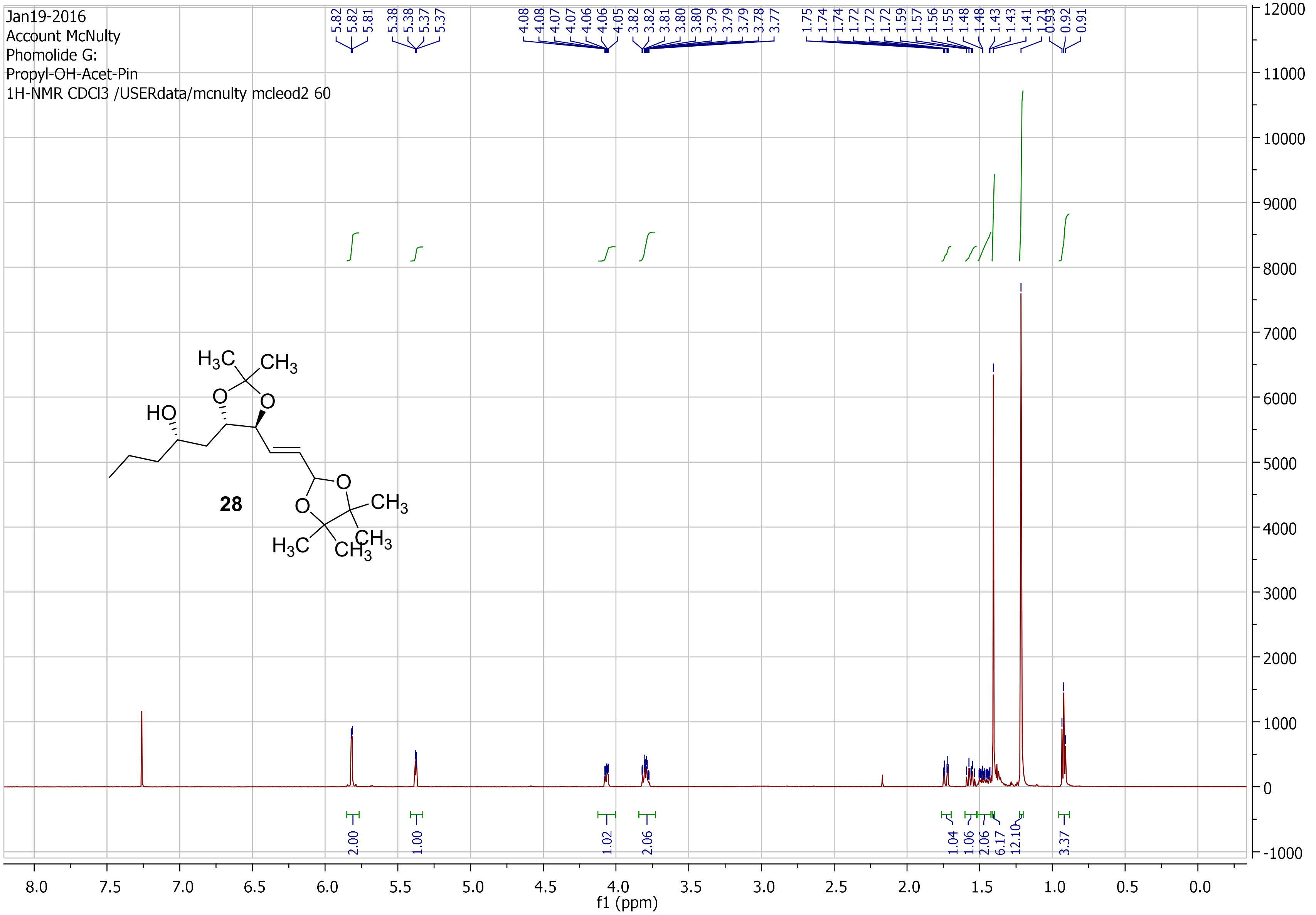

Jan19-2016
Account McNulty
Phomolide G:
Propyl-OH-Acet-Pin
1H-NMR CDCl3 /USERdata/mcnulty mcleod2 60
28
f1 (ppm)

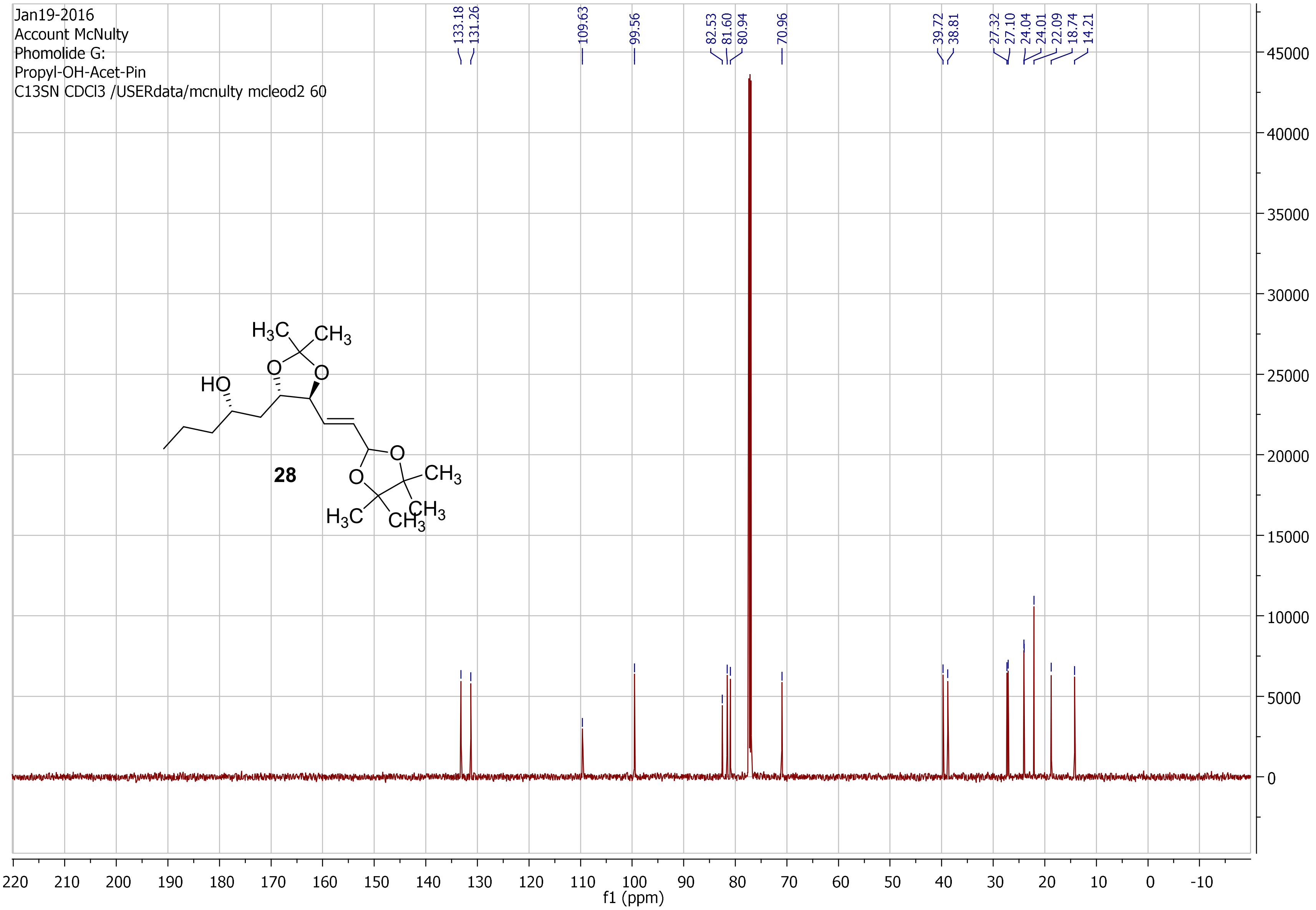

Jan19-2016
Account McNulty
Phomolide G:
Propyl-OH-Acet-Pin
C13SN CDCl3 /USERdata/mcnulty mcleod2 60
133.18
131.26
109.63
99.56
82.53
81.60
80.94
70.96
39.72
38.81
27.32
27.10
24.04
24.01
22.09
18.74
14.21
HO
O
O
28
f1 (ppm)

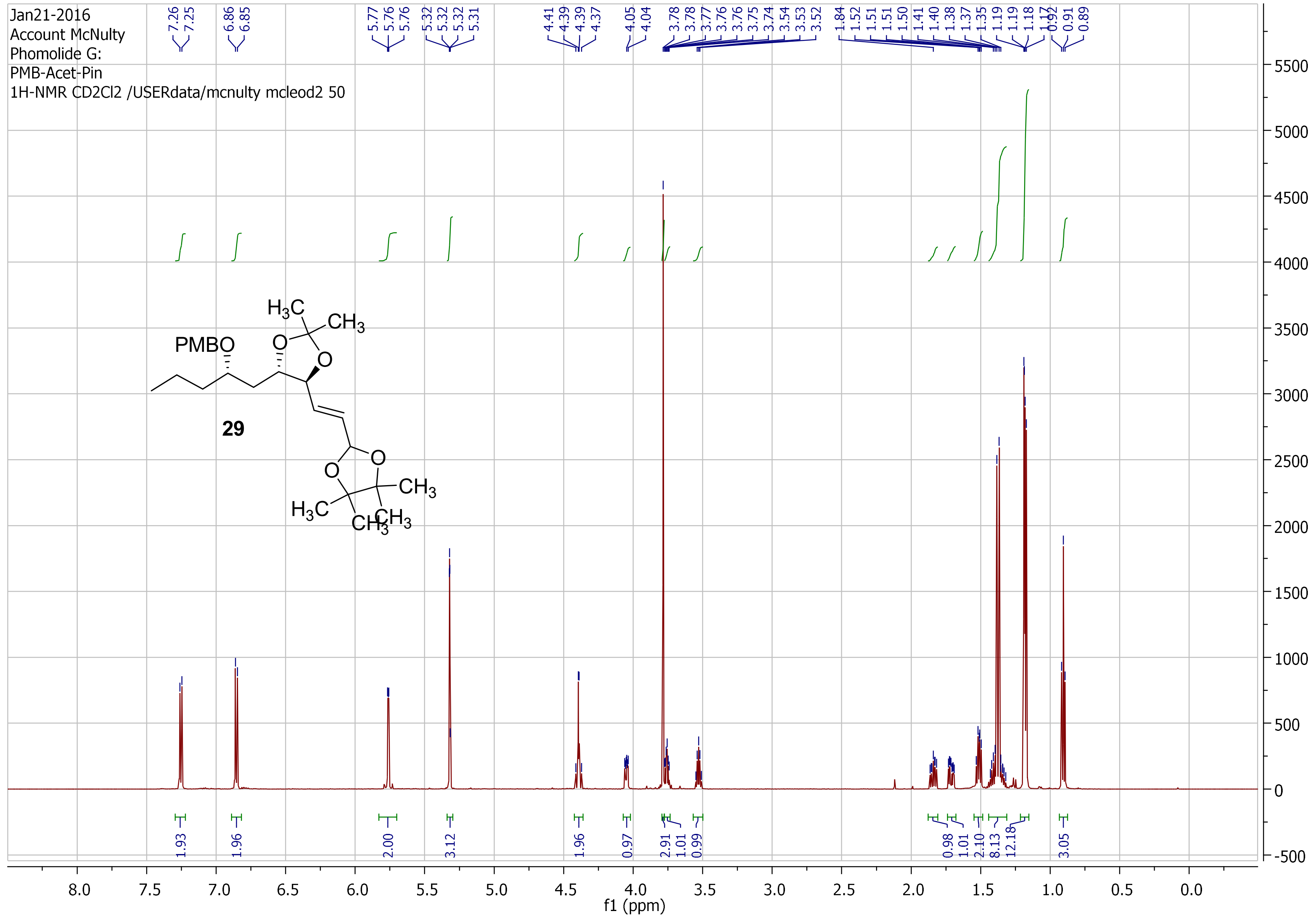
Jan21-2016
Account McNulty
Phomolide G:
PMB-Acet-Pin
1H-NMR CD2Cl2 /USERdata/mcnulty mcleod2 50
PMBO
29
f1 (ppm)

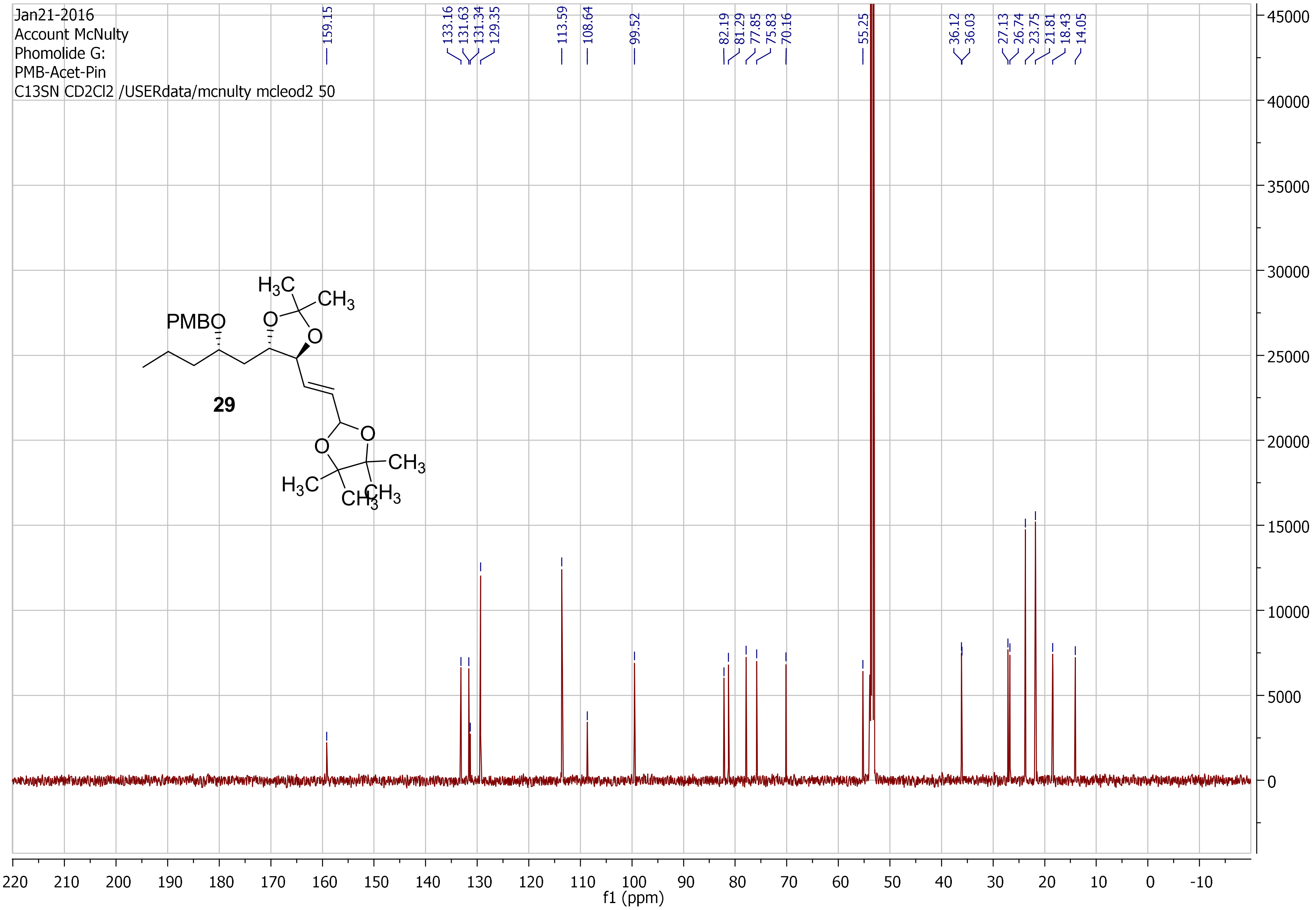
Jan21-2016
Account McNulty
Phomolide G:
PMB-Acet-Pin
C13SN CD2Cl2 /USERdata/mcnulty mcleod2 50
159.15
133.16
131.63
131.34
129.35
113.59
108.64
99.52
82.19
81.29
77.85
75.83
70.16
55.25
36.12
36.03
27.13
26.74
23.75
21.81
18.43
14.05
PMBO
H3C
CH3
O
O
O
O
CH3
H3C
CH3
CH3
29
f1 (ppm)

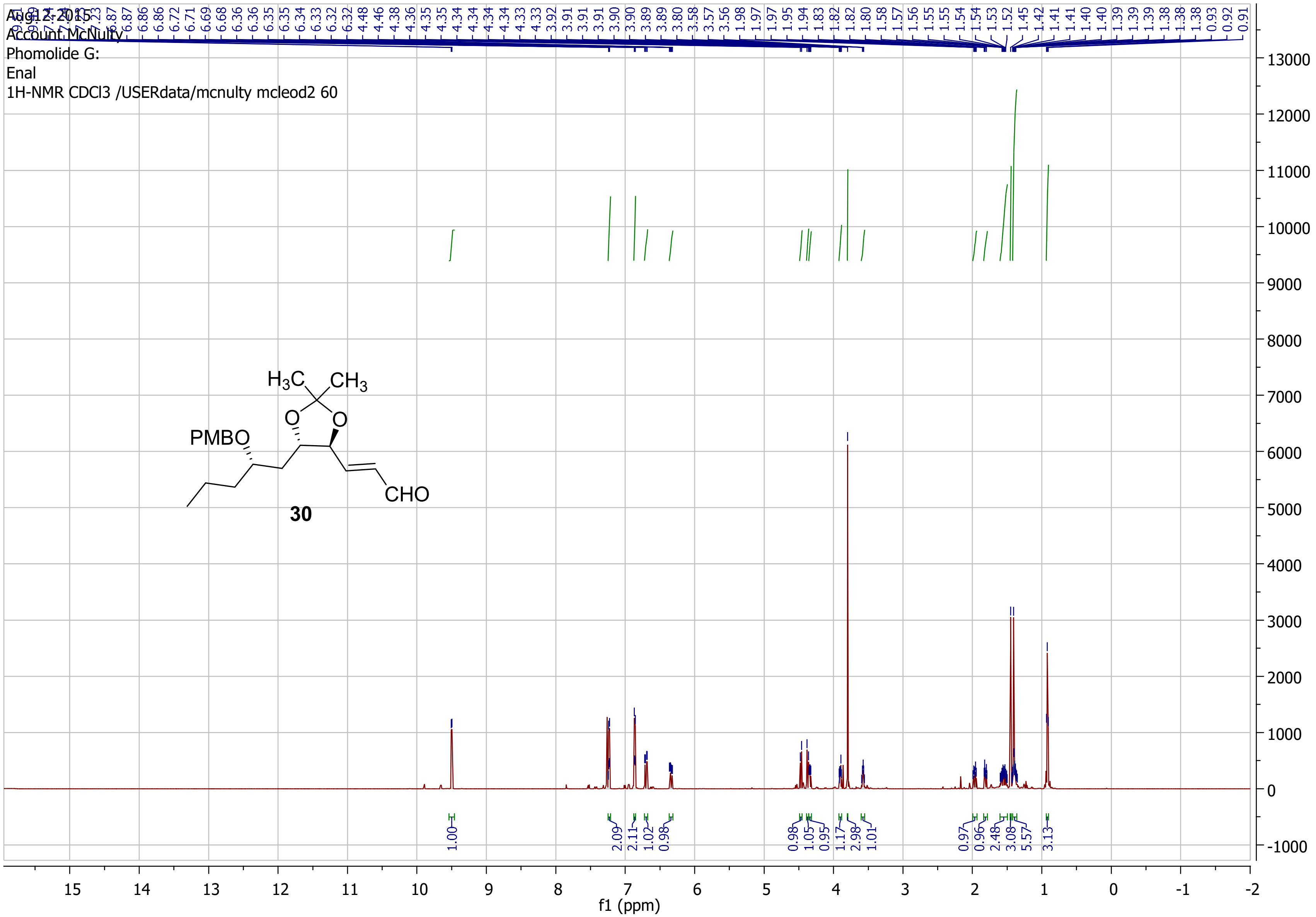
Aug12-2015
Account:McNulty
Phomolide G:
Enal
1H-NMR CDCl3 /USERdata/mcnulty mcleod2 60
H3C
CH3
O
O
PMBO
CHO
30
f1 (ppm)

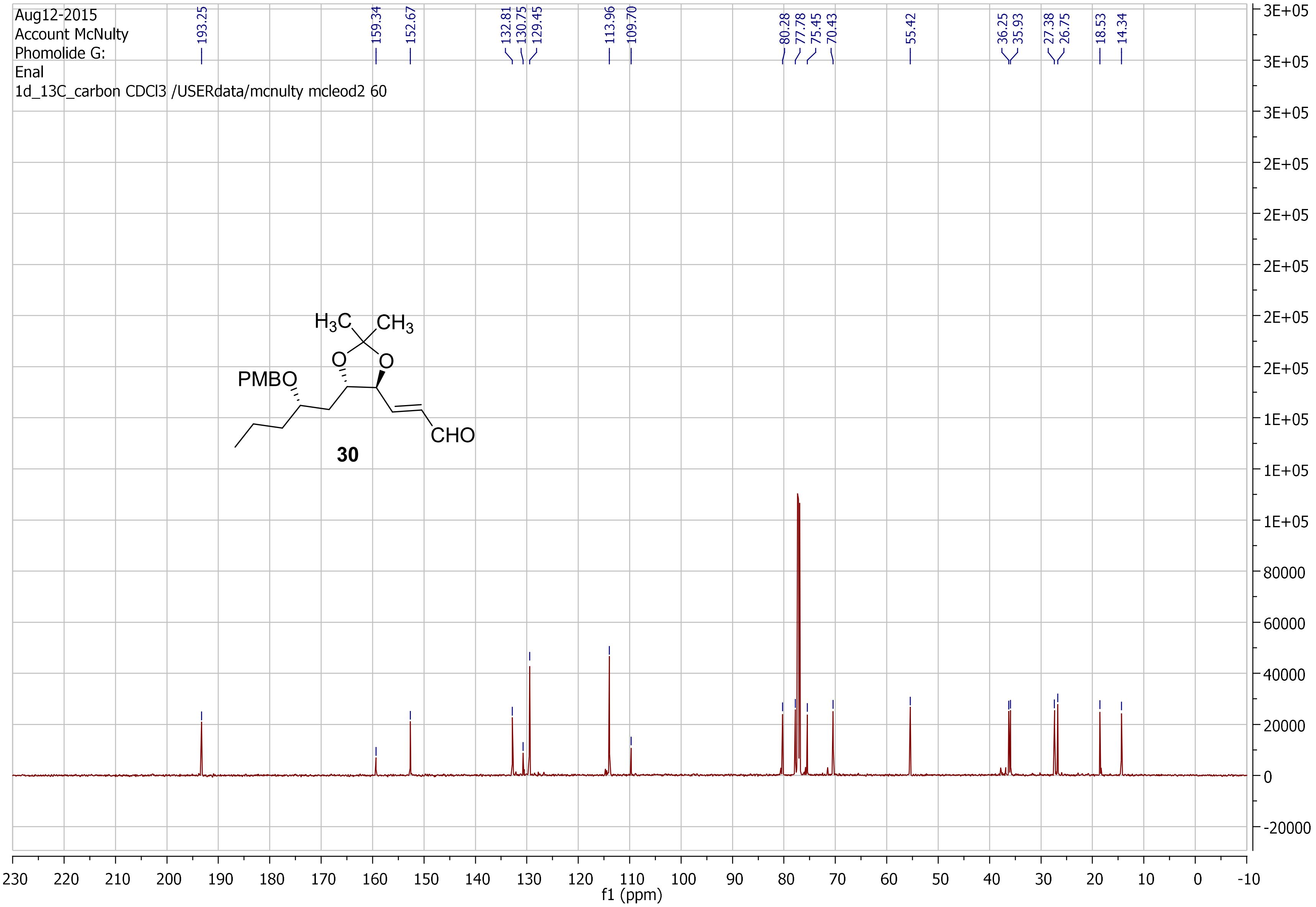
Aug12-2015
Account McNulty
Phomolide G:
Enal
1d_13C_carbon CDCl3 /USERdata/mcnulty mcleod2 60
193.25
159.34
152.67
132.81
130.75
129.45
113.96
109.70
80.28
77.78
75.45
70.43
55.42
36.25
35.93
27.38
26.75
18.53
14.34
H_3C
CH_3
O
O
PMBO
CHO
30
f1 (ppm)

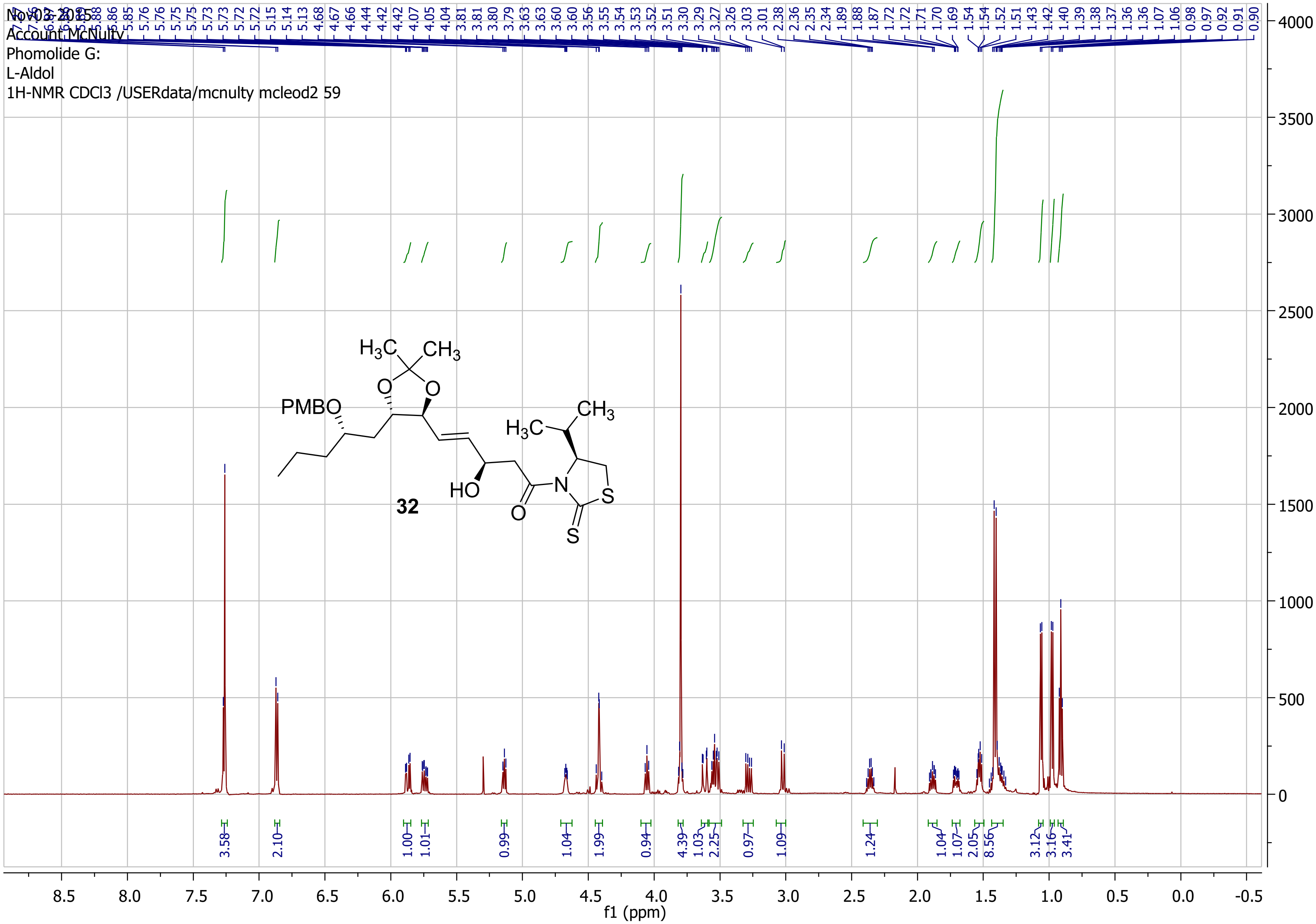
Nov03-2015
Account:McNulty
Phomolide G:
L-Aldol
1H-NMR CDCl3 /USERdata/mcnulty mcleod2 59
PMBO
H3C
CH3
O
O
HO
O
N
S
S
CH3
H3C
32
f1 (ppm)

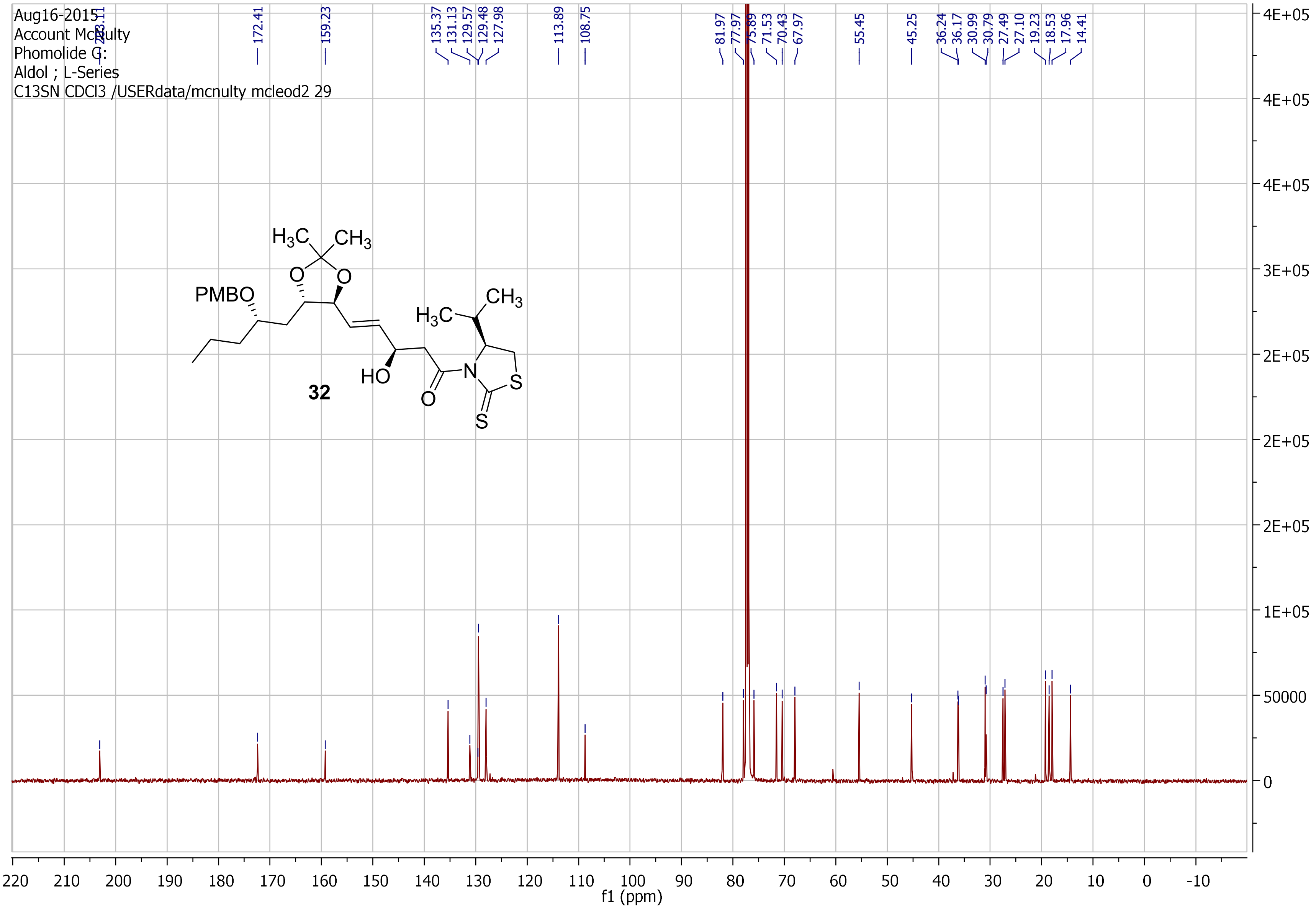

Aug16-2015
Account McNulty
Phomolide G:
Aldol ; L-Series
C13SN CDCl3 /USERdata/mcnulty mcleod2 29
172.41
159.23
135.37
131.13
129.57
129.48
127.98
113.89
108.75
81.97
77.97
75.89
71.53
70.43
67.97
55.45
45.25
36.24
36.17
30.99
30.79
27.49
27.10
19.23
18.53
17.96
14.41
PMBO
H_3C
CH_3
O
O
H_3C
CH_3
HO
N
S
O
S
32
f1 (ppm)
4E+05
3E+05
2E+05
1E+05
50000
0

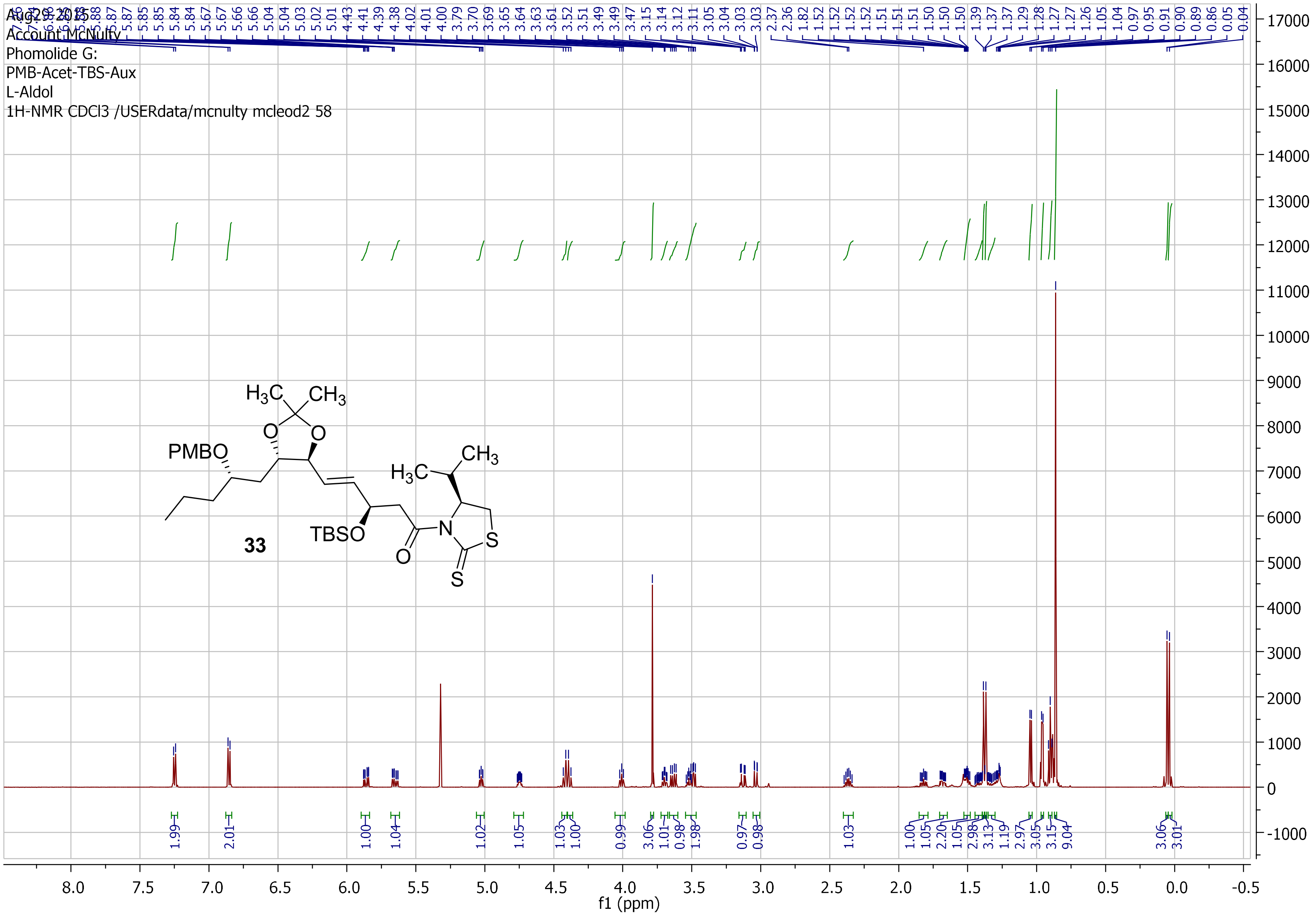
Aug29-2015
Account:McNulty
Phomolide G:
PMB-Acet-TBS-Aux
L-Aldol
1H-NMR CDCl3 /USERdata/mcnulty mcleod2 58
33
f1 (ppm)

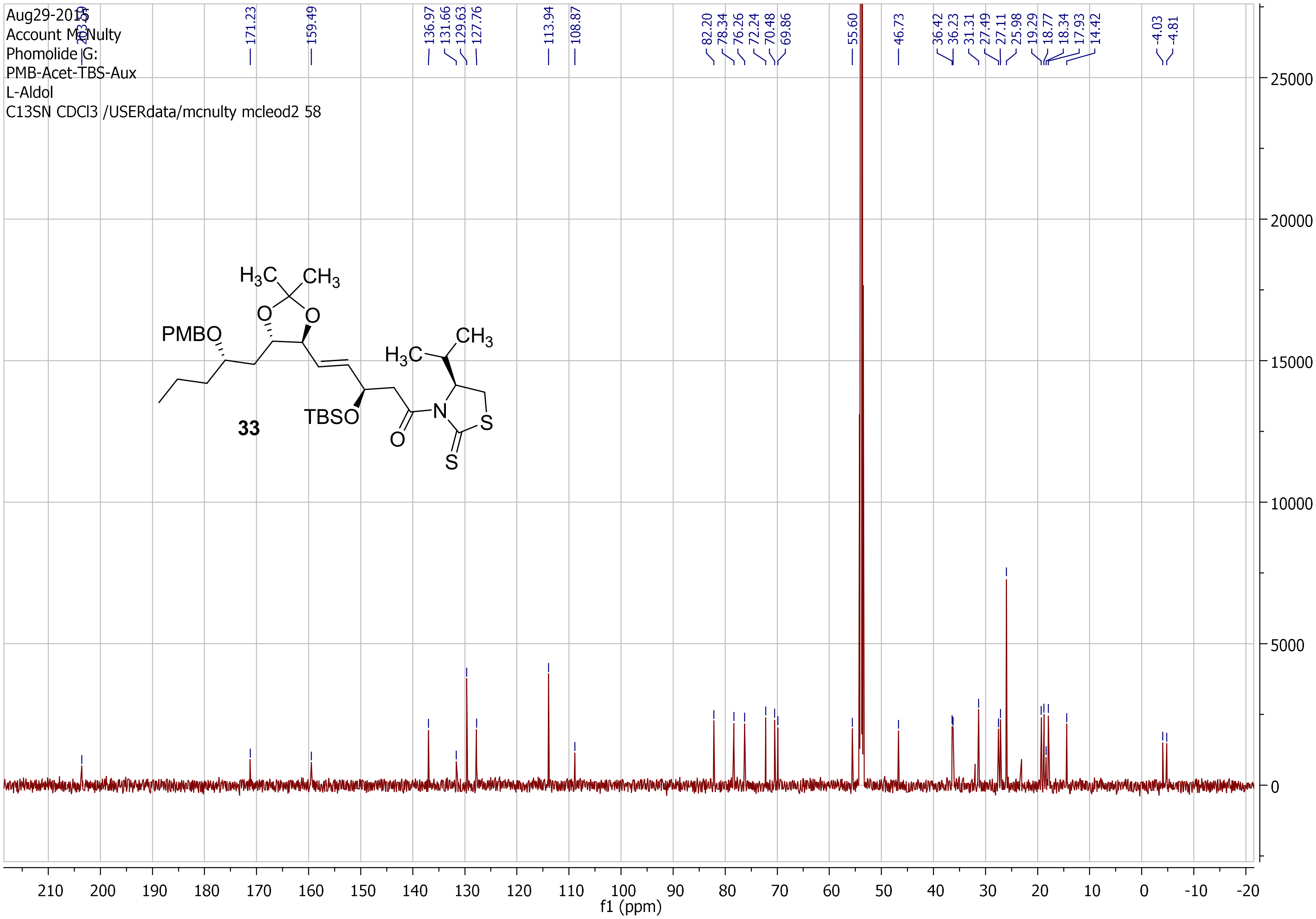
Aug29-2015
Account McNulty
Phomolide G:
PMB-Acet-TBS-Aux
L-Aldol
C13SN CDCl3 /USERdata/mcnulty mcleod2 58
171.23
159.49
136.97
131.66
129.63
127.76
113.94
108.87
82.20
78.34
76.26
72.24
70.48
69.86
55.60
46.73
36.42
36.23
31.31
27.49
27.11
25.98
19.29
18.77
18.34
17.93
14.42
-4.03
-4.81
PMBO
TBSO
33
f1 (ppm)

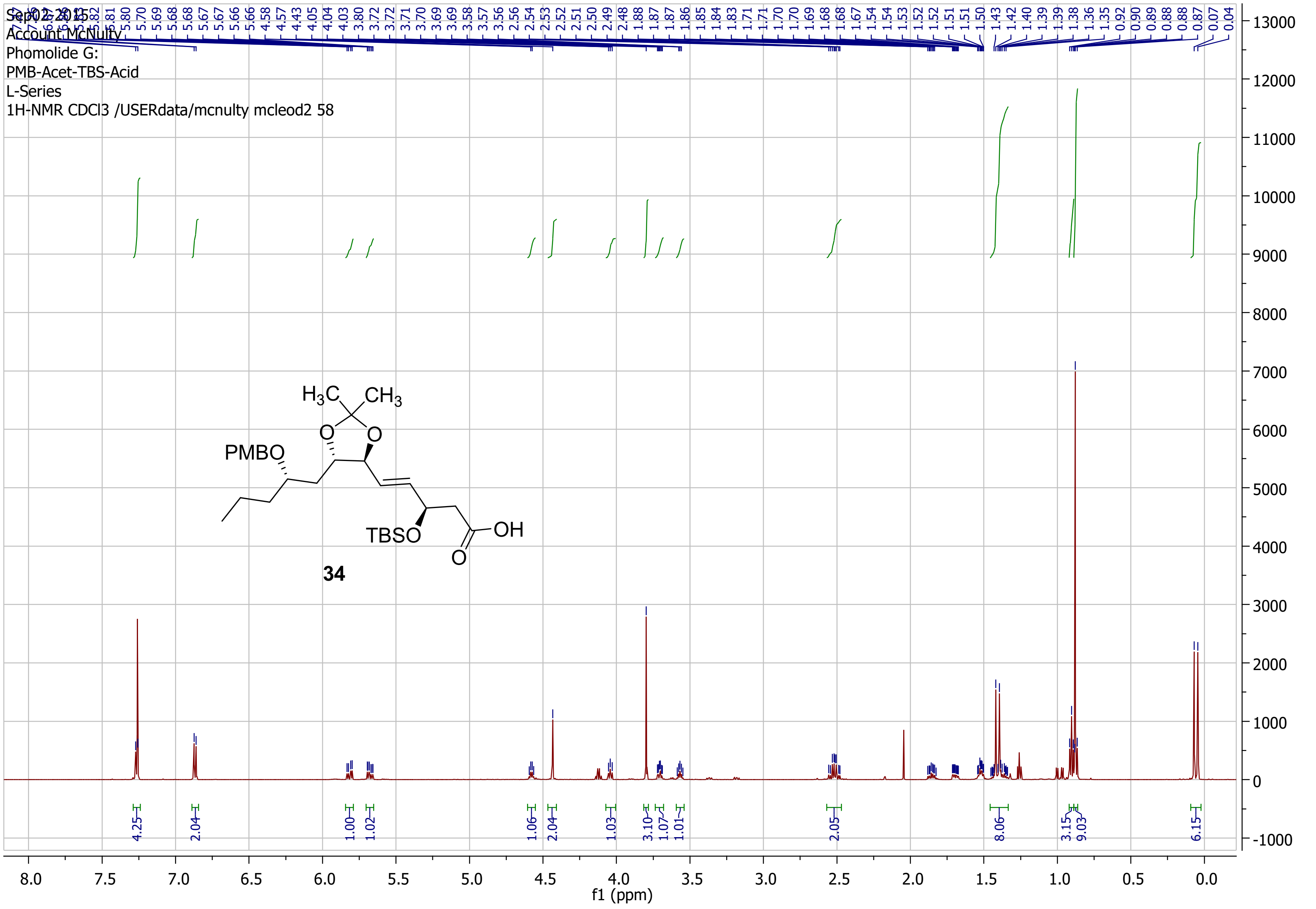
Sep02-2015
Account:McNulty
Phomolide G:
PMB-Acet-TBS-Acid
L-Series
1H-NMR CDCl3 /USERdata/mcnulty mcleod2 58
H3C
CH3
O
O
PMBO
TBSO
OH
O
34
f1 (ppm)

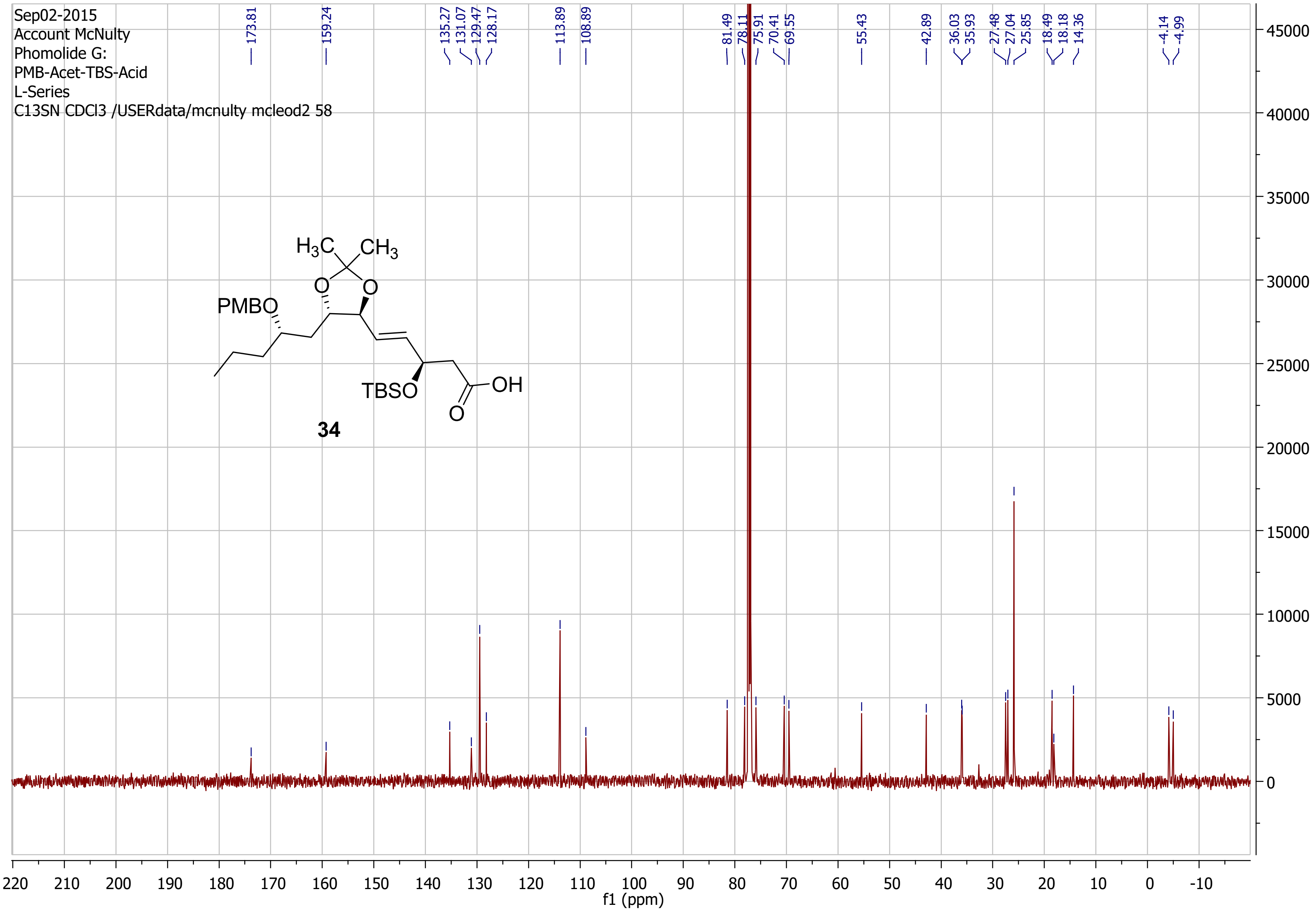
Sep02-2015
Account McNulty
Phomolide G:
PMB-Acet-TBS-Acid
L-Series
C13SN CDCl3 /USERdata/mcnulty mcleod2 58
173.81
159.24
135.27
131.07
129.47
128.17
113.89
108.89
81.49
78.11
75.91
70.41
69.55
55.43
42.89
36.03
35.93
27.48
27.04
25.85
18.49
18.18
14.36
-4.14
-4.99
H3C
CH3
O
O
PMBO
TBSO
OH
O
34
f1 (ppm)

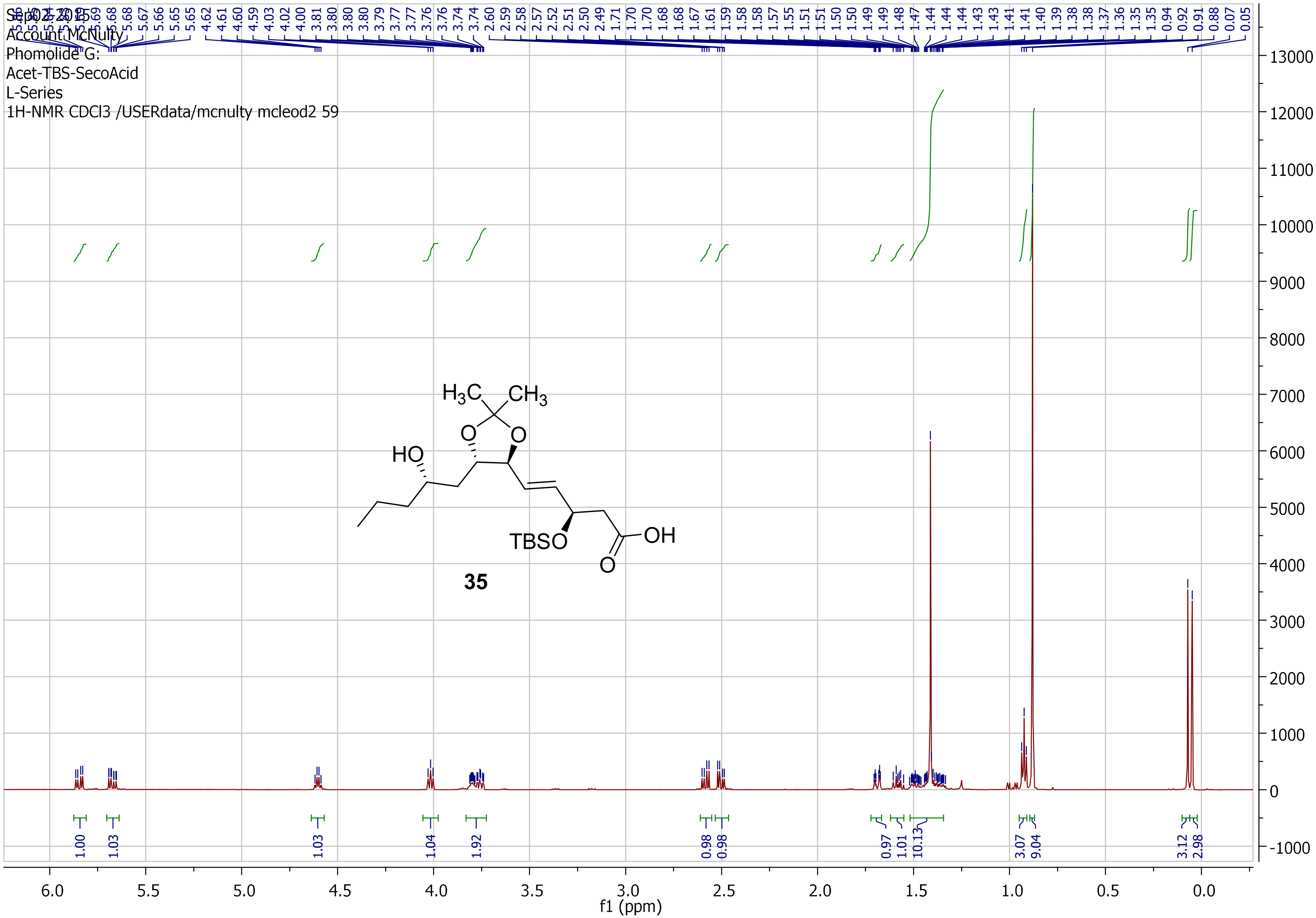
Sep02-2015
Account:McNulty
Phomolide G:
Acet-TBS-SecoAcid
L-Series
1H-NMR CDCl3 /USERdata/mcnulty mcleod2 59
H3C
CH3
O
O
HO
TBSO
OH
O
35
f1 (ppm)

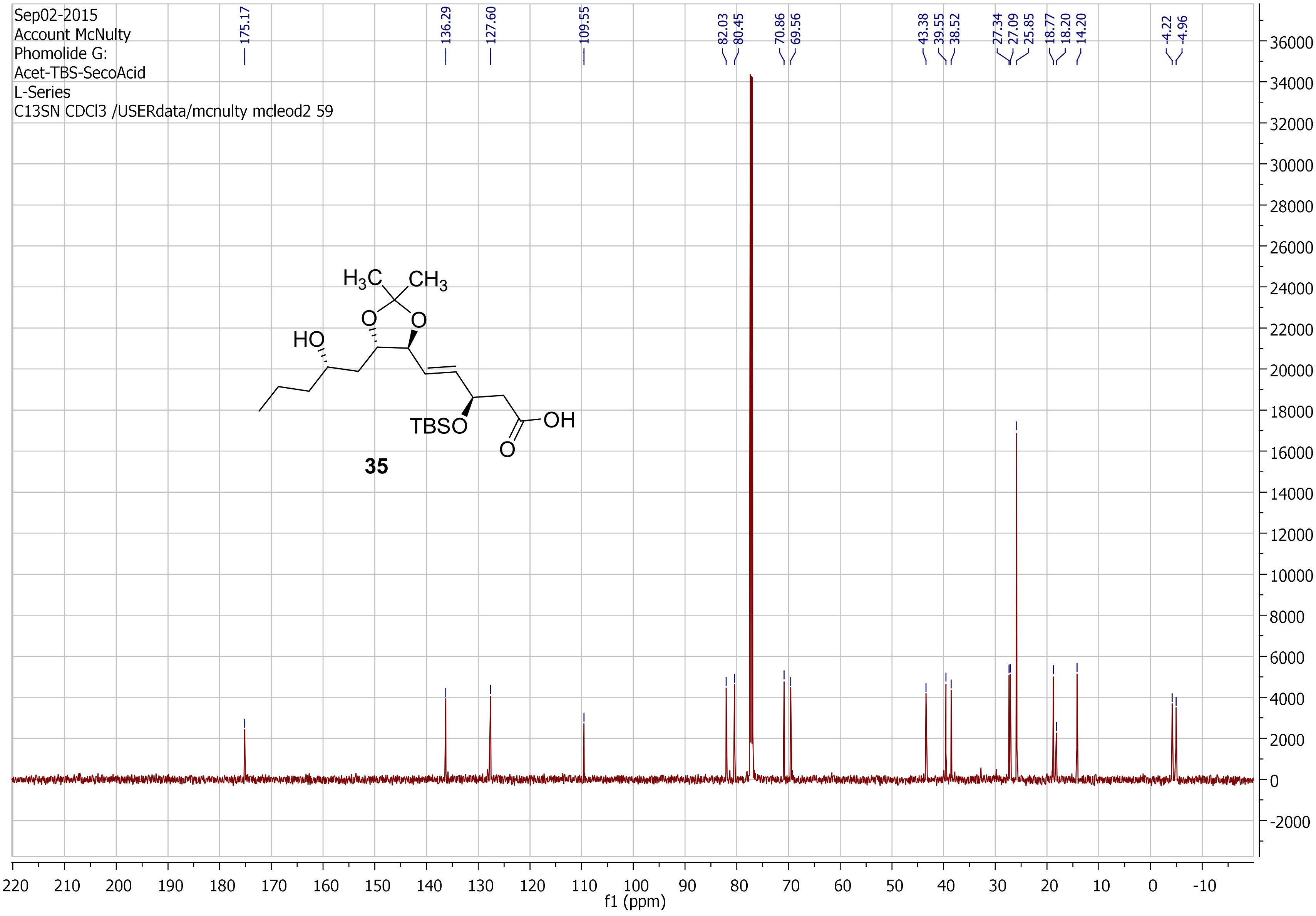
Sep02-2015
Account McNulty
Phomolide G:
Acet-TBS-SecoAcid
L-Series
C13SN CDCl3 /USERdata/mcnulty mcleod2 59
175.17
136.29
127.60
109.55
82.03
80.45
70.86
69.56
43.38
39.55
38.52
27.34
27.09
25.85
18.77
18.20
14.20
-4.22
-4.96
H3C
CH3
O
O
HO
TBSO
OH
O
35
f1 (ppm)

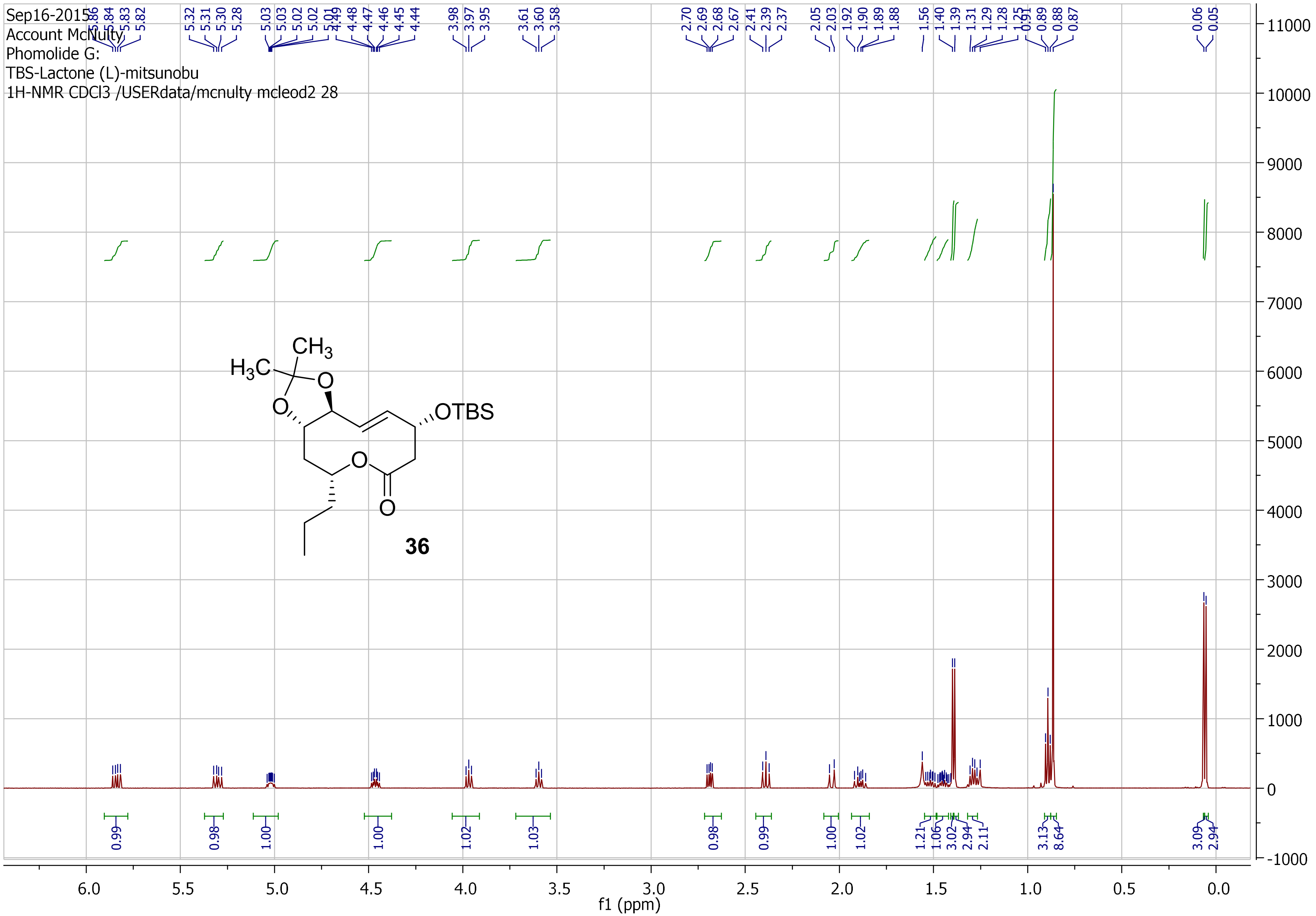

Sep16-2015
Account McNulty
Phomolide G:
TBS-Lactone (L)-mitsunobu
1H-NMR CDCl3 /USERdata/mcnulty mcleod2 28
CH3
H3C
O
O
O
OTBS
O
36
f1 (ppm)

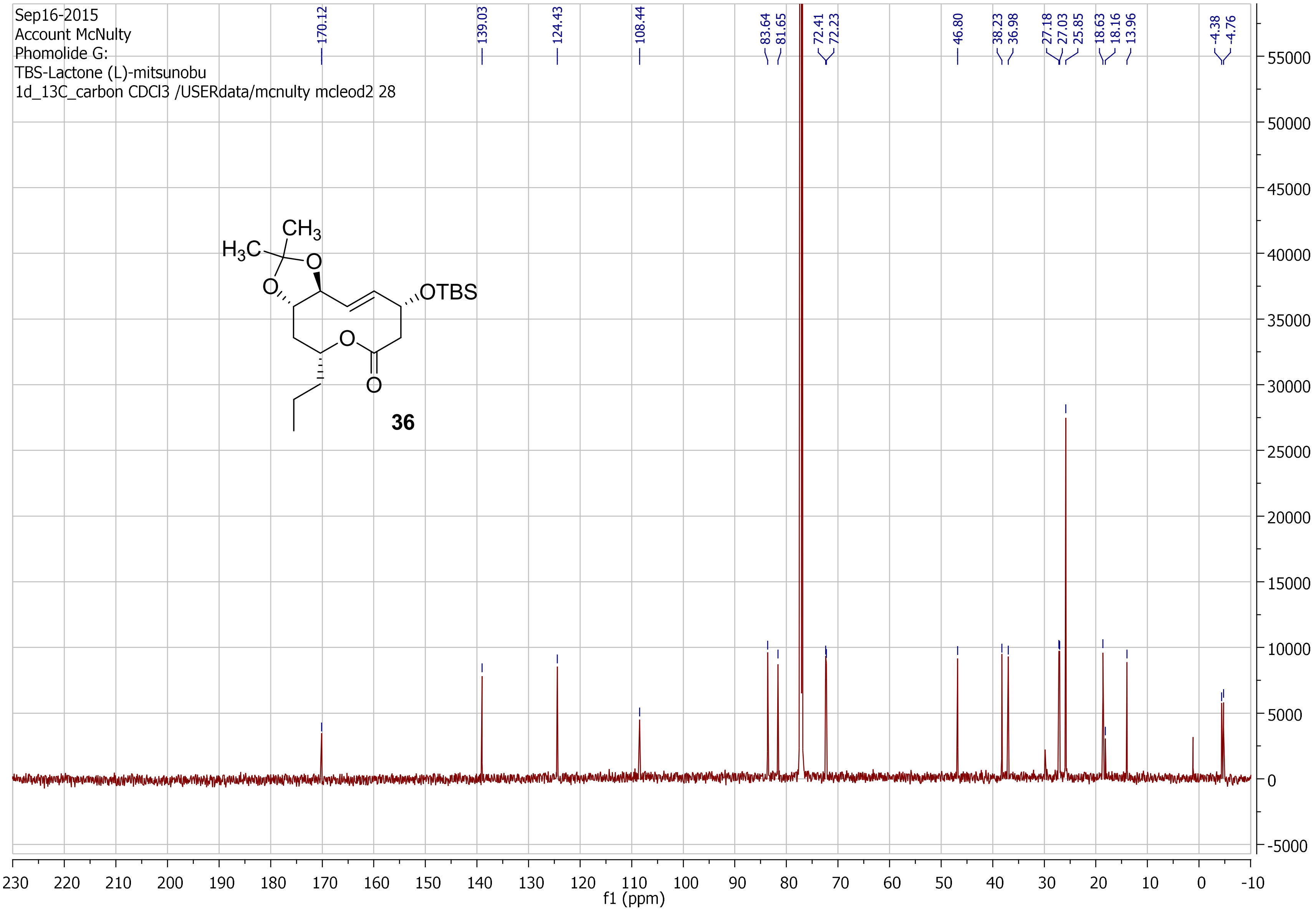
Sep16-2015
Account McNulty
Phomolide G:
TBS-Lactone (L)-mitsunobu
1d_13C_carbon CDCl3 /USERdata/mcnulty mcleod2 28
170.12
139.03
124.43
108.44
83.64
81.65
72.41
72.23
46.80
38.23
36.98
27.18
27.03
25.85
18.63
18.16
13.96
-4.38
-4.76
CH3
H3C
O
O
OTBS
O
O
36
f1 (ppm)

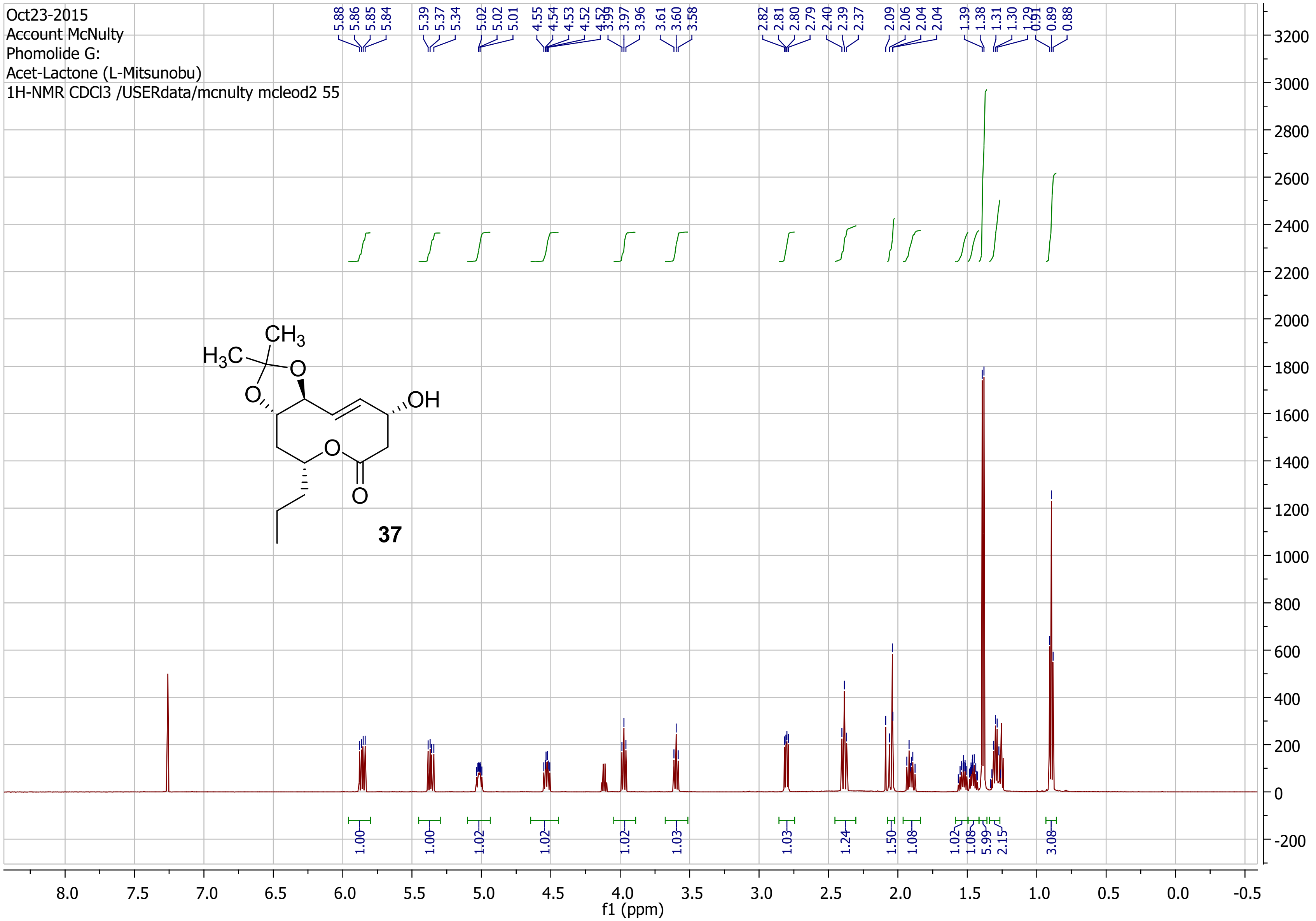
Oct23-2015
Account McNulty
Phomolide G:
Acet-Lactone (L-Mitsunobu)
1H-NMR CDCl3 /USERdata/mcnulty mcleod2 55
5.88 5.86 5.85 5.84 5.39 5.37 5.34 5.02 5.02 5.01 4.55 4.54 4.53 4.52 4.52 3.99 3.97 3.96 3.61 3.60 3.58 2.82 2.81 2.80 2.79 2.40 2.39 2.37 2.09 2.06 2.04 2.04 1.39 1.38 1.31 1.30 1.29 0.91 0.89 0.88
CH3
H3C
O
O
OH
O
O
37
1.00 1.00 1.02 1.02 1.02 1.03 1.03 1.24 1.50 1.08 1.02 1.08 5.99 2.15 3.08
f1 (ppm)

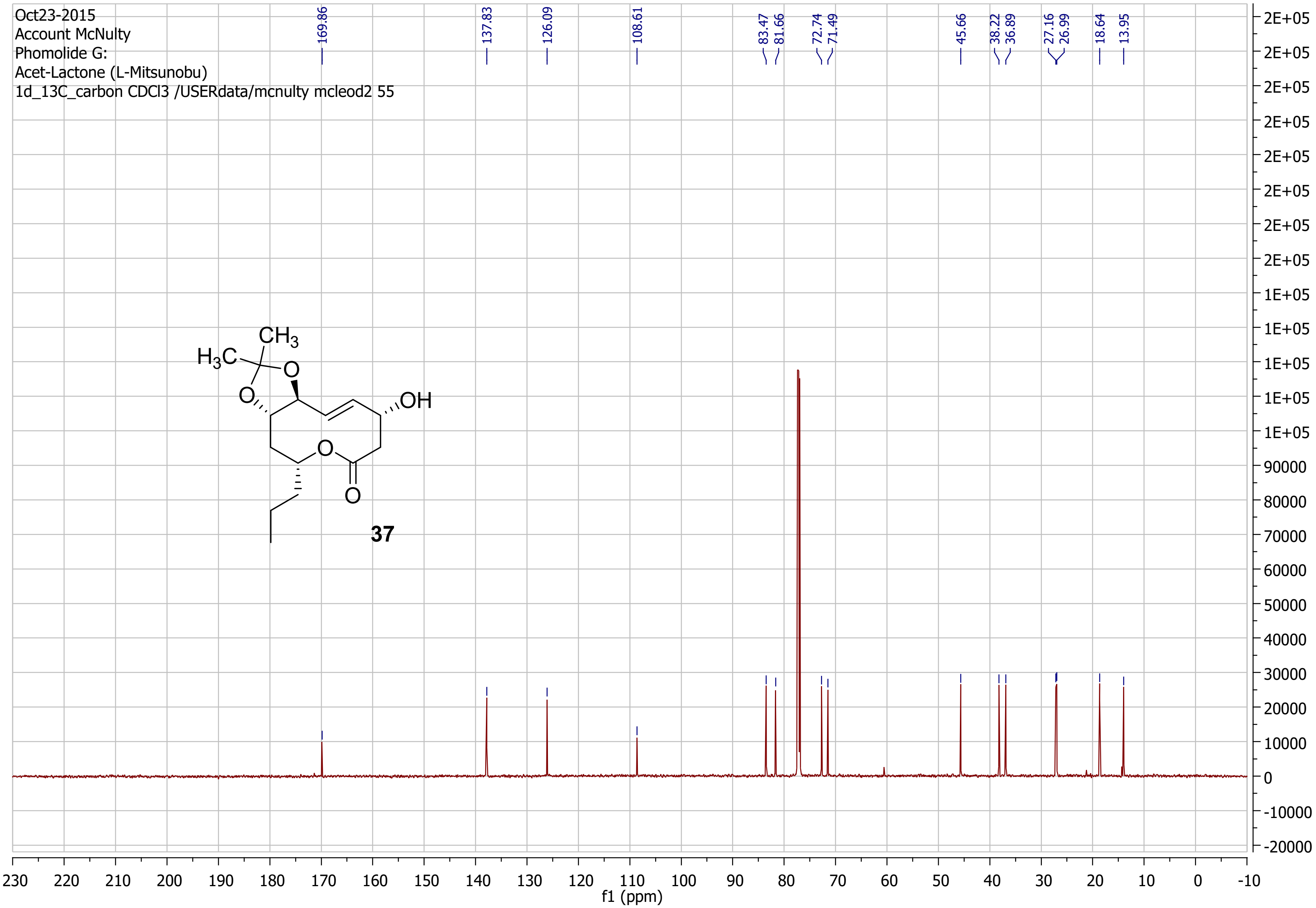

Oct23-2015
Account McNulty
Phomolide G:
Acet-Lactone (L-Mitsunobu)
1d_13C_carbon CDCl3 /USERdata/mcnulty mcleod2 55
169.86
137.83
126.09
108.61
83.47
81.66
72.74
71.49
45.66
38.22
36.89
27.16
26.99
18.64
13.95
37
f1 (ppm)

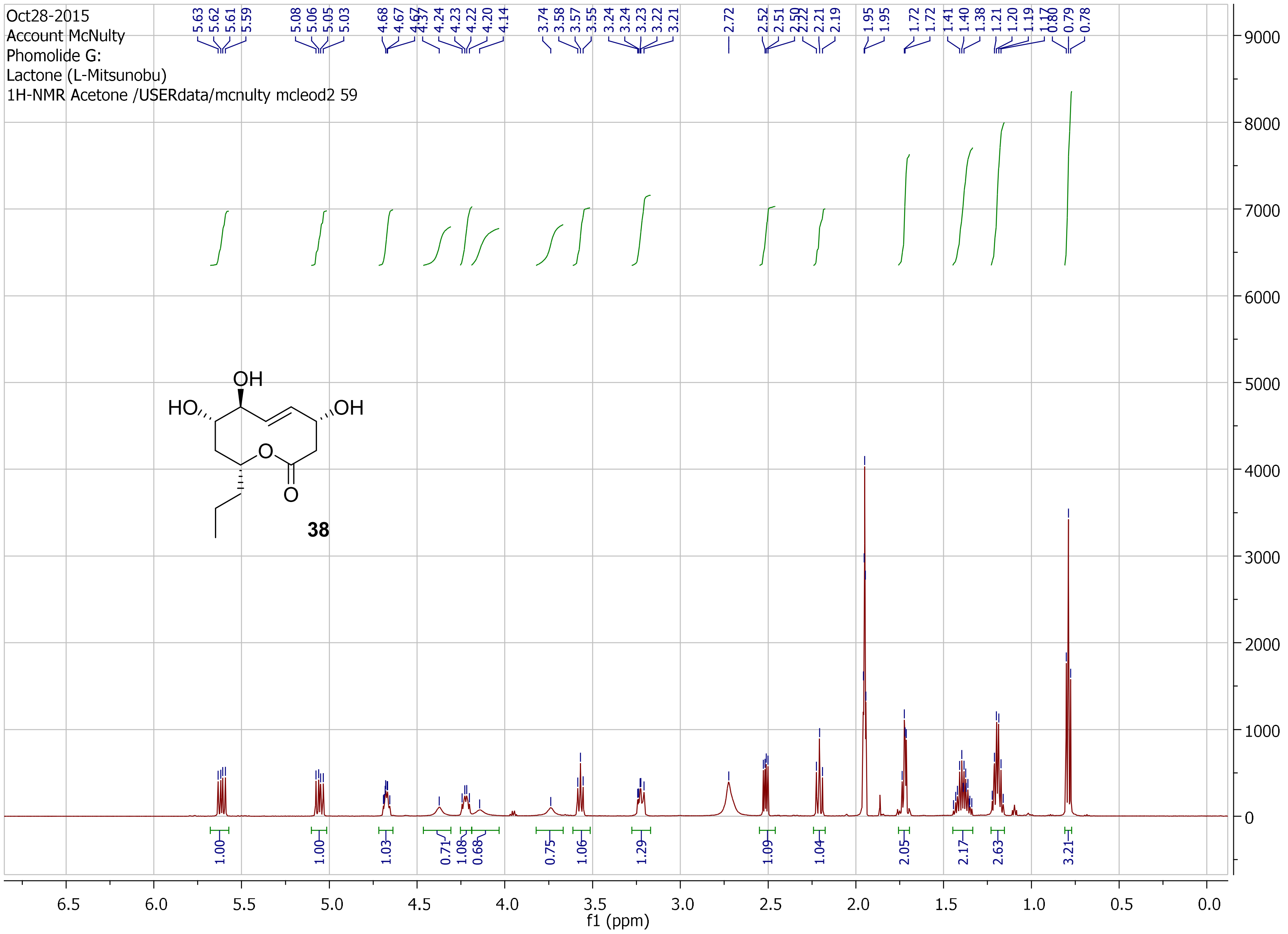
Oct28-2015
Account McNulty
Phomolide G:
Lactone (L-Mitsunobu)
1H-NMR Acetone /USERdata/mcnulty mcleod2 59
HO
OH
OH
O
O
38
f1 (ppm)

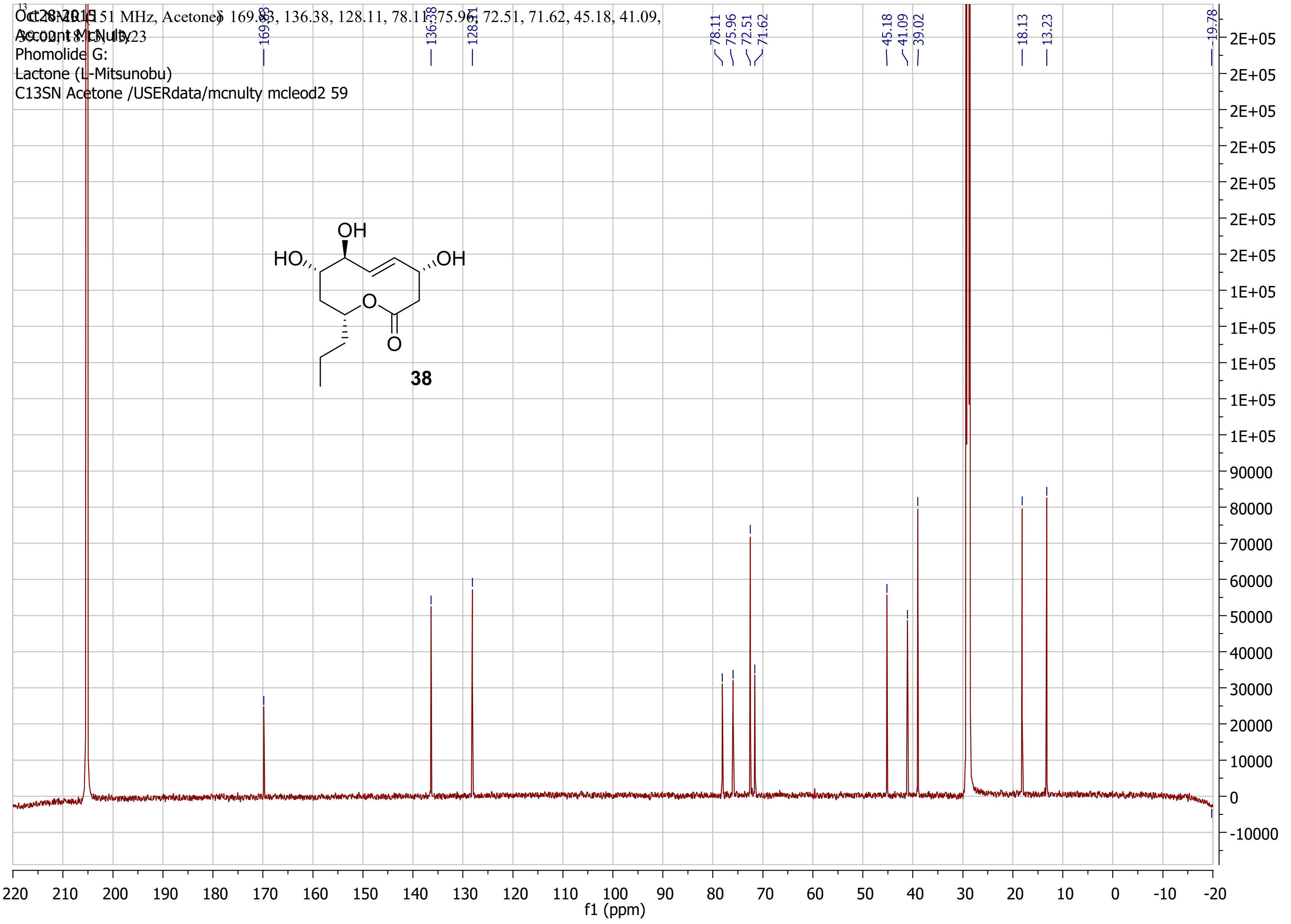
Oct28-2015
Account McNulty
Phomolide G:
Lactone (L-Mitsunobu)
C13SN Acetone /USERdata/mcnulty mcleod2 59
72.51, 71.62, 45.18, 41.09,
169.83
136.38
128.11
78.11
75.96
72.51
71.62
45.18
41.09
39.02
18.13
13.23
-19.78
HO
OH
OH
O
O
38
f1 (ppm)

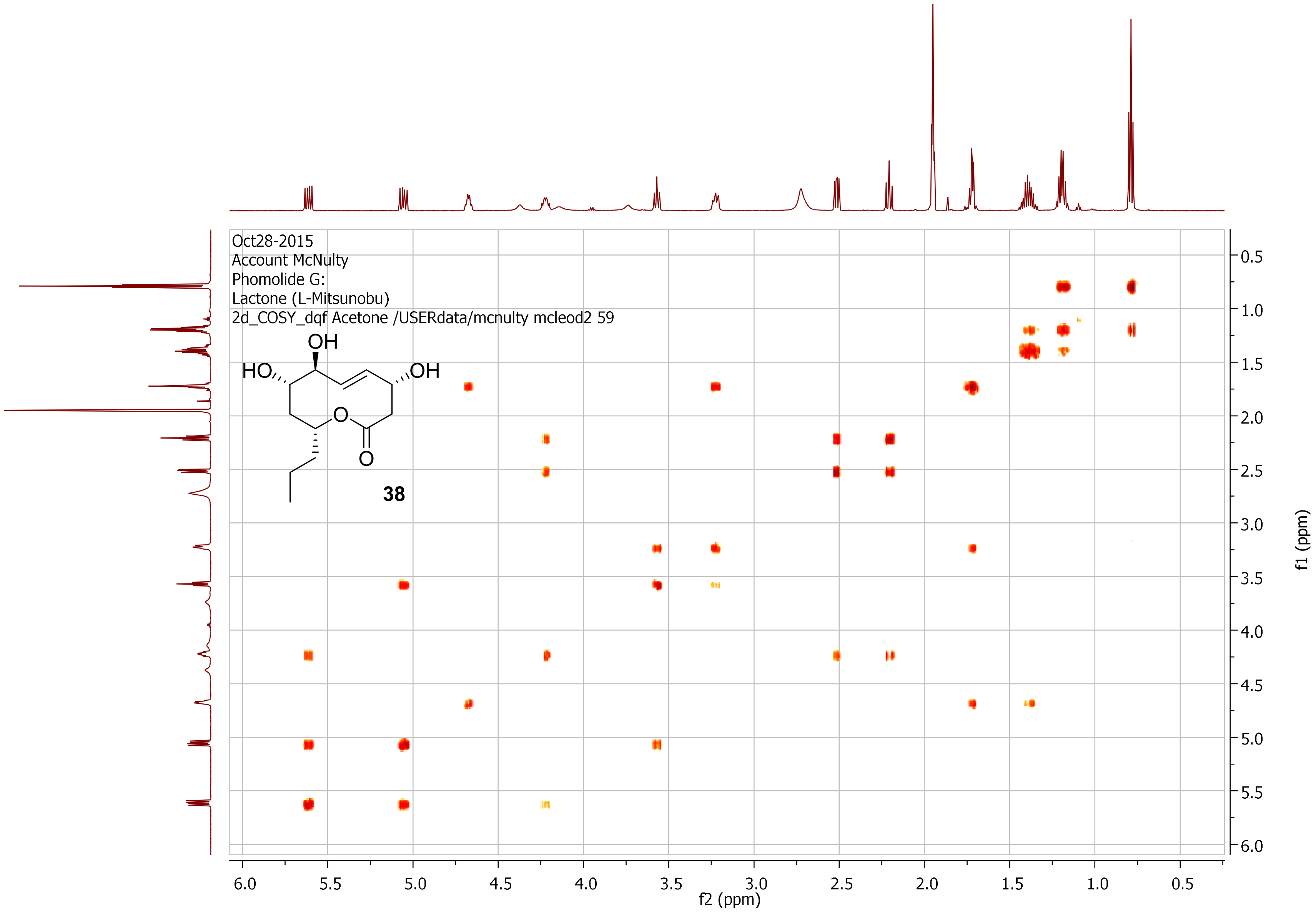
Oct28-2015
Account McNulty
Phomolide G:
Lactone (L-Mitsunobu)
2d_COSY_dqf Acetone /USERdata/mcnulty mcleod2 59
OH
HO
OH
O
O
38
f2 (ppm)
f1 (ppm)

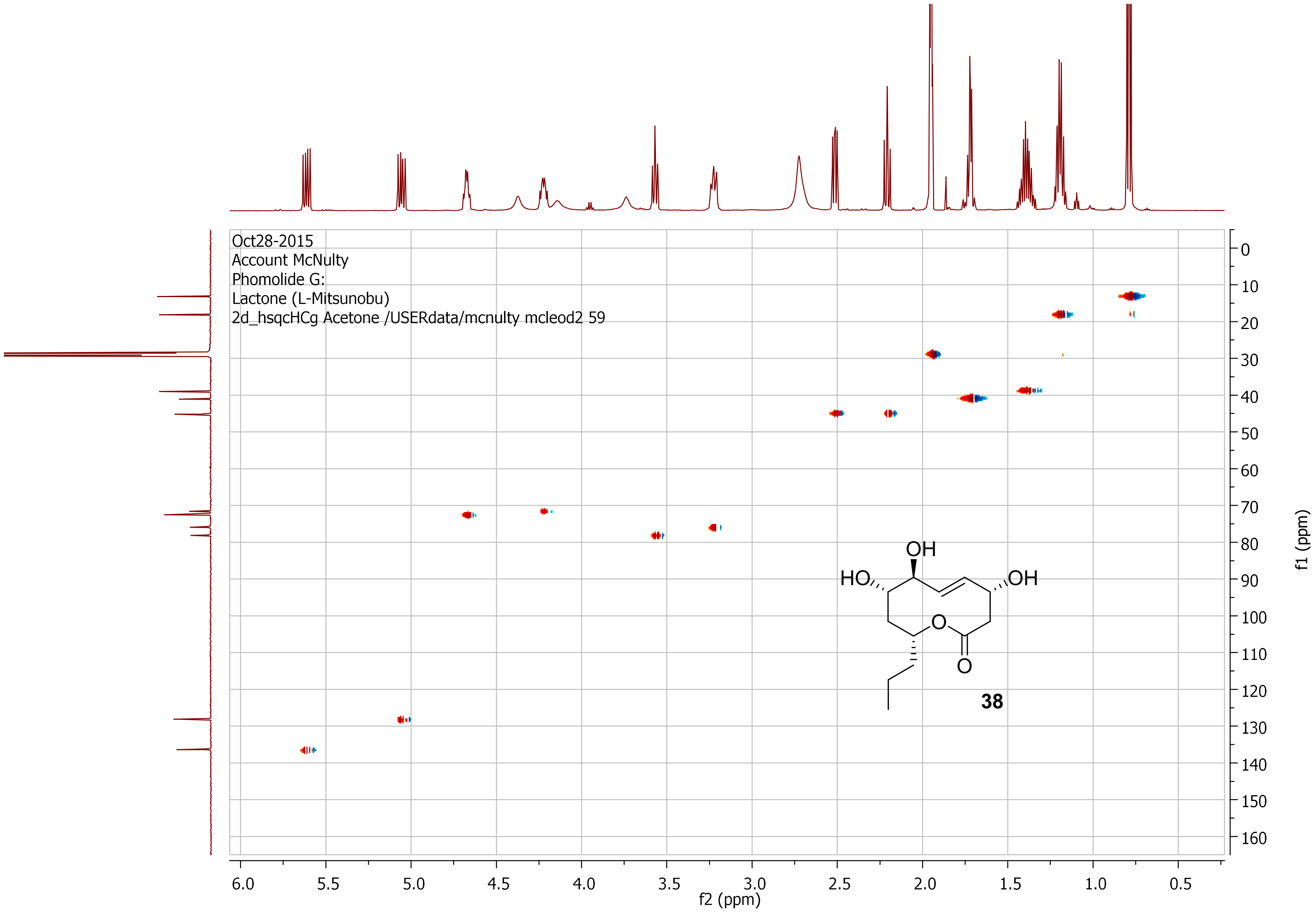

Oct28-2015
Account McNulty
Phomolide G:
Lactone (L-Mitsunobu)
2d_hsqcHCg Acetone /USERdata/mcnulty mcleod2 59
OH
HO
OH
O
O
38
f2 (ppm)
f1 (ppm)
